# Disclosing the effects of pea-derived proteins on the human gut microbiota

**DOI:** 10.20517/mrr.2025.77

**Published:** 2025-11-25

**Authors:** Leonardo Mancabelli, Christian Milani, Giulia Longhi, Gabriele Andrea Lugli, Chiara Tarracchini, Francesca Turroni, Marco Ventura

**Affiliations:** ^1^Department of Medicine and Surgery, University of Parma, Parma 43125, Italy.; ^2^Interdepartmental Research Centre “Microbiome Research Hub”, University of Parma, Parma 43124, Italy.; ^3^Laboratory of Probiogenomics, Department of Chemistry, Life Sciences and Environmental Sustainability, University of Parma, Parma 43124, Italy.

**Keywords:** Microbiota, yellow pea, pea protein, monoculture

## Abstract

**Aim:** Plant-derived proteins have emerged as promising alternatives to animal-based proteins, offering not only environmental and nutritional benefits to the human host but also potential effects on the gut microbiota. Yellow pea (*Pisum sativum*) represents an attractive source due to its balanced amino acid composition and suitability for food applications. This preliminary study was designed to evaluate the effects of two commercial pea-derived protein preparations - a wet-extracted protein isolate (PPI) and a dry-fractionated protein concentrate (PPC) - on the human gut microbiota using a dual *in vitro* approach.

**Methods:** We combined monoculture assays on selected representative intestinal bacterial strains with *in vitro* cultivation models of stabilized microbial communities derived from human fecal samples.

**Results:** Monoculture experiments revealed selective growth responses in certain taxa, such as *Bacteroides thetaiotaomicron* and *Bifidobacterium* spp. Moreover, *in silico* genomic predictions of amino acid biosynthesis and proteolytic capabilities further supported these findings, highlighting functional differences among the tested strains. Furthermore, analysis based on stabilized microbial communities revealed moderate shifts in microbial richness and composition. Notably, PPC was associated with greater variation in taxonomic profiles across samples. Both protein ingredients exhibited similar directional effects on specific taxa, including increases in the load of *Bifidobacterium longum* and *Faecalibacterium duncaniae*, and decreases in members of *Bacteroides*, *Parabacteroides*, and *Phocaeicola*.

**Conclusion:** These findings indicate that pea-derived proteins, especially when used as concentrates, exert selective pressure on gut microbial communities.

## INTRODUCTION

In the context of growing global demand for sustainable and nutritious protein sources, legumes have garnered considerable interest as alternative raw materials for developing plant-based protein products^[1]^. Chickpeas, lentils, and peas are among the most widely investigated pulses for this purpose, due to their valuable protein content, favorable amino acid profile, and adaptability to diverse agricultural conditions^[2]^. Beyond their nutritional value, legumes offer advantages related to sustainability, including lower environmental impact and potential for local cultivation in various regions of the world^[3]^.

Among legumes, yellow pea (*Pisum sativum*) has emerged as a particularly promising source for the production of texturized vegetable proteins (TVPs) and other functional protein ingredients^[4]^. Compared to soybean, which is traditionally the primary source of plant protein isolates (PPIs), pea proteins carry a lower allergenic potential, are less frequently genetically modified, and allow the development of a European-based supply chain^[5]^.

The industrial processing of pea proteins yields two primary products with distinct features: PPIs and protein concentrates (PPCs)^[6]^. PPIs are typically produced through wet fractionation methods, such as alkaline solubilization followed by isoelectric precipitation, achieving high protein purity levels (~ 80%-90%). However, these processes often involve significant use of chemicals and harsh conditions that can partially denature proteins, potentially impacting their techno-functional properties and nutritional quality^[6]^.

In contrast, protein amounts are often produced by dry fractionation techniques, such as micronization and air classification. These processes yield products with moderate protein content (~ 50%-60%) while preserving a higher proportion of native protein structures and soluble fractions, such as albumins. Additionally, dry fractionation avoids the use of chemicals and requires less water and energy, making it a more sustainable and environmentally friendly option. Notably, concentrates may retain functional compounds that could enhance techno-functional performance and possibly exert positive effects on human health^[6]^.

Beyond their role as protein sources, legumes and their derivatives are increasingly studied for their potential to influence the composition of the human gut microbiota, i.e., the complex community of microorganisms that plays a crucial role in digestion, immune function, and overall health^[7-9]^. Diet is one of the most significant factors shaping these microbial communities, and different plant-derived ingredients may promote the growth of beneficial bacteria or modulate metabolic activities in the gut^[10]^.

While the effects of legume consumption on the gut microbiota have been explored to some extent^[8,11]^, there is still limited knowledge on how specific pea protein ingredients, particularly concentrates and isolates, might differently impact microbial composition or activity. Given that these products differ not only in protein content but also in processing methods and the presence of other bioactive compounds, it is important to investigate whether they exert distinct effects on gut bacteria.

This preliminary study was therefore designed to evaluate the influence of PPC and PPI on bacterial growth and the composition of the human gut microbiota under *in vitro* conditions, considering that differences in processing and composition may influence the interaction between pea-derived proteins and the human gut microbiota.

## METHODS

### Plant-based protein preparations

Two protein-rich preparations from yellow pea (*Pisum sativum*) were included in this study, both provided by MartinoRossi SpA. The products differed markedly in origin and processing methods. The first, referred to as PPI, was a commercially available ingredient obtained through conventional wet fractionation, involving solubilization of proteins under alkaline conditions followed by isoelectric precipitation^[6]^. In contrast, the second, referred to as PPC, was developed in-house by MartinoRossi SpA using a dry fractionation method, based on micronization and air classification of pea flour^[6]^. The macronutritional composition of the two products, expressed per 100 g, is summarized in Table 1, with values provided by the respective manufacturers (MartinoRossi SpA for PPC and the commercial supplier for PPI).

**Table 1:** Macronutritional composition of the two pea-derived protein preparations (g/100 g)

**Component**	**PPC**	**PPI**
Fat	4	7
Carbohydrates	12.4	5
(of which sugars)	1.6	1
Fiber	10.9	7
Protein	59.3	70
Salt	0	2.2

PPC: Pea protein concentrate; PPI: pea protein isolate.

### Bacterial strains and *in vitro* growth assays on plant-based protein preparations

Seven bacterial species were selected as representative members of the human intestinal microbiota, covering key taxonomic and functional groups commonly found in the colon ecosystem [Table 2]. The panel included *Bacteroides thetaiotaomicron* (*B. thetaiotaomicron*) and *Segatella copri* (*S. copri*), taxa frequently associated with distinct human enterotypes, as well as *Faecalibacterium prausnitzii* (*F. prausnitzii*), a major producer of short-chain fatty acids, and four widely studied bifidobacterial species with known probiotic potential. All strains were revitalized from glycerol stocks stored at -80 °C and cultivated overnight at 37 °C in a human gut-simulating culture medium (IGSM)^[12]^ under anaerobic conditions. Strict anaerobes were grown in Hungate tubes, while the remaining strains were incubated in anaerobic polypropylene tubes. Strains were obtained from in-house culture collections.

**Table 2:** Cultivation conditions of the selected bacterial strains

**Strains**	**Temperature**	**Cultivation conditions**
*Bacteroides thetaiotaomicron* 41F	37 °C	Anaerobiosis, polypropylene tubes
*Bifidobacterium adolescentis* ATCC 15703	37 °C	Anaerobiosis, polypropylene tubes
*Bifidobacterium bifidum* LMG 11041	37 °C	Anaerobiosis, polypropylene tubes
*Bifidobacterium breve* LMG 13208	37 °C	Anaerobiosis, polypropylene tubes
*Bifidobacterium longum* subsp. *longum* LMG 13197	37 °C	Anaerobiosis, polypropylene tubes
*Faecalibacterium prausnitzii* DSM 17677	37 °C	Anaerobiosis, Hungate tubes
*Segatella copri* DSM 18205	37 °C	Anaerobiosis, Hungate tubes

ATCC: American Type Culture Collection; LMG: Laboratorium voor Microbiologie Gent Culture Collection; DSM: Deutsche Sammlung von Mikroorganismen und Zellkulturen.

To assess the potential effects of the two plant-based protein preparations, i.e., PPI and PPC, on microbial growth, 100 µL of each revitalized bacterial culture were inoculated into 10 mL of IGSM supplemented with PPI or PPC at final concentrations of 0.5%, 1%, and 2% (w/v). Control cultures containing no added protein preparation were included for each strain. Prior to inoculation, the desired amount of PPI or PPC was mixed with the culture medium, and the entire mixture was sterilized by autoclaving at 121 °C for 15 min.

After inoculation, all cultures were incubated anaerobically at 37 °C for 24 h. Bacterial growth was then quantified by total cell count using flow cytometry. Each experimental condition was tested in triplicate.

### Evaluation of bacterial cell density by flow cytometry

Each culture was diluted in physiological solutions [phosphate-buffered saline (PBS)] to determine the total cell count. Subsequently, 1 mL of the obtained bacterial cell suspension was stained with 1 μL of SYBR® Green I (ThermoFisher Scientific, USA) (1: 100 dilution in dimethyl sulfoxide; Sigma, Germany), vortex-mixed, and incubated in the dark for at least 15 min before measurement. All cells count experiments were performed using the Attune NxT Flow Cytometry (ThermoFisher Scientific, USA), equipped with a blue laser set at 50 mW and tuned to an excitation wavelength of 488 nm. Multiparametric analyses were performed on both scattering signals, i.e., forward scatter (FSC) and side scatter (SSC), while SYBR® Green I fluorescence was detected on the BL1 (Blue Laser) 530/30 nm optical detector. Cell debris was excluded from acquisition analyses by setting a BL1 threshold. Furthermore, the gated fluorescence events were evaluated on the forward-sideways density plot to exclude remaining background events and to obtain an accurate microbial cell count, as previously described^[13]^. All data were statistically analyzed using Attune NxT flow cytometry software.

### *In silico* prediction of proteolytic capacity and protein secretion

The proteolytic potential of the strains used in monoculture assays was assessed via Basic Local Alignment Search Tool-protein (BLASTp) analysis against the MEROPS database of peptidases and inhibitors^[14]^. Protein sequences from each strain were retrieved from the National Center for Biotechnology Information Reference Sequence (NCBI RefSeq) database and queried locally against a custom-formatted MEROPS protein database stored in FASTA format using BLASTp with an e-value cutoff of 1e-30. Hits meeting or exceeding this threshold were retained and considered putative peptidases. Subsequently, identified protein sequences were subjected to secretion prediction using SignalP v6.0^[15]^, which allows discrimination of signal peptides secreted via the Sec/SPI (general secretory pathway, SP), Sec/SPII (lipoprotein signal peptide, LIPO), Tat, and other bacterial pathways. This allowed the identification of extracellular enzymes potentially involved in environmental protein degradation.

### Cultivation of the gut microbiota under *in vitro* conditions

Six complex bacterial communities were obtained from fecal samples collected from healthy adult individuals participating in a broader research initiative, the Parma Microbiota project^[16]^. Donors had not received probiotics, prebiotics, or antibiotics for at least three months prior to collection. Once in the laboratory, fecal samples were transferred to an anaerobic workstation at 37 °C, where the tubes were briefly uncapped to allow gas exchange and remove oxygen. Subsequently, stool samples were homogenized, gauze-filtered, and immediately inoculated in the culture medium at a final inoculum concentration of 2% (v/v), as previously described^[17]^. For each complex bacterial community, three growth conditions were tested: IGSM alone, IGSM supplemented with 2% (w/v) PPI, and IGSM supplemented with 2% (w/v) PPC. The percentage of the plant-based preparation added to the culture medium was selected based on the results obtained in the growth assay analysis. Cultivations were conducted in 1 mL of each selected growth condition, following the MiPro model^[17]^, which involved a 96-deep well plate covered with a silicone gel mat provided with a vent hole in each well, created using a syringe needle. The plate was incubated under anaerobic conditions at 37 °C and shaken at 500 rpm. After 24 h, cultivations were collected and preserved at -20 °C until processing. The experiments were performed in triplicate for each tested condition and fecal sample.

### DNA extraction and shallow shotgun sequencing

Each cultivation replicate was subjected to DNA extraction using the QIAamp® DNA Stool Mini Kit following the manufacturer's instructions (Qiagen, Germany). The extracted DNA was then prepared using the Illumina Nextera XT DNA Library Preparation Kit following the Illumina Nextera XT protocol. Briefly, DNA samples were enzymatically fragmented, barcoded, and purified employing magnetic beads. Subsequently, samples were quantified using a fluorometric Qubit quantification system (Life Technologies, USA), loaded onto a 2200 Tape Station Instrument (Agilent Technologies, USA), and normalized to 4 nM. Paired-end sequencing was performed using an Illumina NextSeq 2000 sequencer with a NextSeq reagent Kit (Illumina Inc., San Diego, USA) together with a deliberate spike-in of 1% PhiX control library.

### Analysis of shallow shotgun metagenomic datasets

The raw data in FASTQ format were filtered to remove reads with a quality score < 25 and sequences mapping to the human genome (*Homo sapiens*). Reads longer than 149 bp were retained for further analysis. Quality-filtered reads were then used for taxonomic profile reconstruction by using the METAnnotatorX2 bioinformatic platform^[18]^. Retained sequences were used as input to perform a MegaBLAST local alignment of reads to a pre-processed database, following the METAnnotatorX2 protocol^[18]^. Reads with > 94% identity to a reference genome were assigned at the species level, while reads below this threshold were classified at the genus level as undefined species.

### Statistical analysis

Statistical Package for the Social Sciences (SPSS) software was used to perform statistical analysis on the different culture cell counts using the Kruskal-Wallis test.

Biodiversity within a given sample (alpha-diversity) was calculated using a species richness index and evaluated using the Kruskal-Wallis test. Similarities between samples (beta-diversity) were calculated using the Bray-Curtis dissimilarity index. Principal coordinate analysis (PCoA) representations of beta-diversity were performed using QIIME2 (https://qiime2.org/)^[19,20]^, and differences in community composition were tested using permutational multivariate analysis of variance (PERMANOVA) through pseudo-F statistic. Moreover, non-parametric Spearman rank correlations were computed between the relative abundance of each taxon and the plant-based protein ingredients (coded as a binary variable). Correlation analyses were performed in R v4.3.1, and *P*-values < 0.05 were considered significant. All correlation *P*-values were adjusted for multiple comparisons using the Benjamini-Hochberg method.

## RESULTS

### Growth response of selected intestinal bacterial taxa to pea-based protein preparations

To evaluate the potential impact of plant-based protein ingredients obtained through different processing technologies on several key members of the human intestinal microbiota, we assessed the growth performance of 7 representative bacterial strains exposed to 2 commercially available protein-rich preparations derived from yellow peas. The two tested ingredients were a PPI and a PPC, produced respectively by wet extraction and dry fractionation. The selected taxa were chosen for their ecological relevance within the gut ecosystem. These included *B. thetaiotaomicron* and *S. copri* (previously classified as *Prevotella copri*), two highly prevalent and abundant commensals in the human gut microbiota, often representing dominant community members in different individuals^[21,22]^. Their selection is consistent with the concept of enterotype-like stratification observed across populations^[23]^. The panel also included *F. prausnitzii*, a well-known butyrate producer^[24,25]^, along with four species of *Bifidobacterium* that have been documented to have potential health-beneficial features^[26]^.

**Figure 1:**
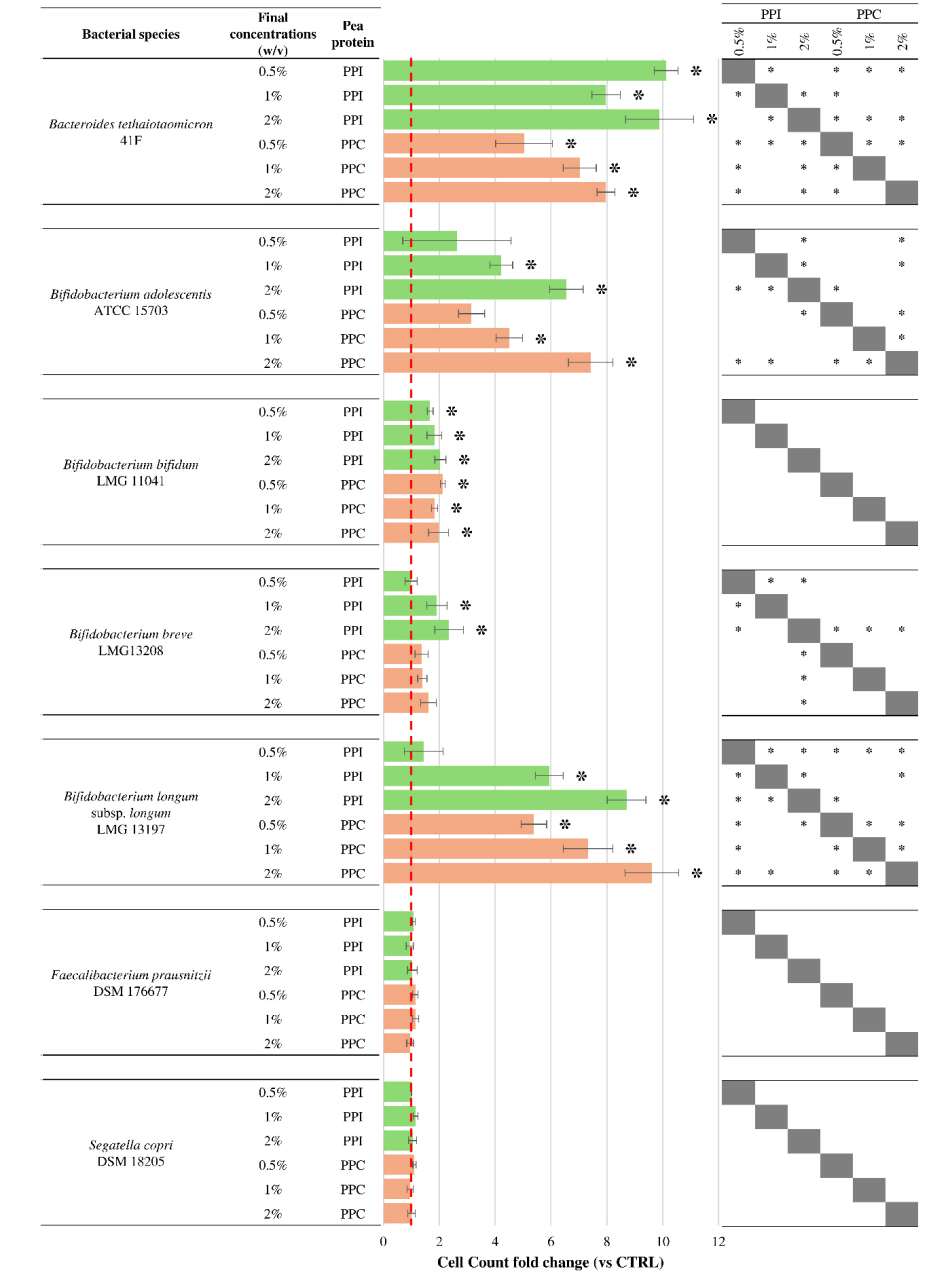
Growth response of selected intestinal bacterial strains to pea protein preparations. The bar plots display the fold change in cell count after 24 h of anaerobic incubation with increasing concentrations (0.5%, 1%, and 2% w/v) of PPI (green bars) and PPC (orange bars), relative to the untreated control. The dashed red line represents the fold change value of 1, corresponding to control levels. Error bars represent propagated standard deviations across biological replicates. Asterisks (*) indicate statistically significant differences compared to the control (Kruskal-Wallis test, *P* < 0.05). The table on the right summarizes the results of pairwise comparisons between PPI and PPC at the same concentrations. Asterisks (*) indicate statistically significant differences between the two protein preparations (Kruskal-Wallis test, *P* < 0.05). PPC: Pea protein concentrate; PPI: pea protein isolate; ATCC: American Type Culture Collection; LMG: Laboratorium voor Microbiologie Gent Culture Collection; DSM: Deutsche Sammlung von Mikroorganismen und Zellkulturen; CTRL: control.

Each strain was cultured under anaerobic conditions in the presence of increasing concentrations of PPI or PPC, specifically 0.5%, 1%, and 2% w/v. After 24 h of incubation, bacterial growth was measured using flow cytometry [Supplementary Table 1] and the results were expressed as fold change compared to control conditions without supplementation [Figure 1].

A general stimulation of bacterial growth was observed for most of the tested species, with responses varying based on the strain and the concentration of the protein preparation.

Among the most responsive taxa, *B. thetaiotaomicron*, *Bifidobacterium longum* (*B. longum*), and *Bifidobacterium bifidum* displayed a consistent growth enhancement across all tested concentrations and with both PPI and PPC (Kruskal-Wallis test *P*-value < 0.05). The response of *B. thetaiotaomicron* was particularly marked and dose-dependent, in line with its known ability to metabolize a broad range of dietary proteins and peptides^[27]^. *B. longum* showed a similar trend, though with slightly more moderate increases, while *B. bifidum* exhibited lower responses, with fold change values generally ranging between 1 and 2. This behavior may reflect more limited metabolic flexibility or slower growth dynamics under the tested conditions.

Conversely, *Bifidobacterium adolescentis* and *B. breve* did not exhibit any significant growth change at the lowest concentration tested (Kruskal-Wallis test *P*-value > 0.05). A noticeable increase was observed only at concentrations of 1% and 2% w/v (Kruskal-Wallis test *P*-value < 0.05), suggesting that a threshold concentration is required to trigger a metabolic response in these strains. Moreover, the growth increase of *B. breve* was statistically significant only in the presence of PPI, suggesting a preference for the type of soluble nitrogen compounds and peptides preserved through wet fractionation. These differences are consistent with the known variability among *Bifidobacterium* species in their ability to utilize different nitrogen sources^[28,29]^.

In contrast, *F. prausnitzii* and *S. copri* did not show any significant growth change for any of the tested conditions. Their specific nutritional requirements may explain this lack of response. In fact, *F. prausnitzii* is a strict anaerobe that thrives on fermentable carbohydrates and cross-fed metabolites such as acetate and lactate^[30,31]^. Similarly, *S. copri* is typically associated with carbohydrate-rich diets and specializes in the degradation of complex polysaccharides, rather than proteins^[32,33]^. The absence of a detectable growth response in these species reinforces the hypothesis that pea-derived protein preparations are more likely to support microbes that are metabolically adapted to nitrogen-rich substrates^[34]^.

These findings suggest that PPI and PPC may selectively influence gut microbial communities, promoting the growth of proteolytic bacteria such as *Bacteroides* and specific *Bifidobacterium* species, while exerting minimal effects on fiber-dependent taxa. This targeted modulation, based on metabolic specialization, highlights potential opportunities for the dietary use of legume-derived proteins as microbiota-shaping ingredients in functional food applications.

### *In silico* prediction of amino acid biosynthesis and proteolytic potential of the used strains

To explore the genetic features related to the amino acid biosynthesis and proteolytic potential of the selected bacterial strains, *in silico* analyses were assessed to predict the capacity to degrade proteins and synthesize amino acids *de novo*, focusing on mechanisms potentially relevant to the utilization of legume-derived protein substrates.

Biosynthetic potential was investigated using GapMind web-tool^[35]^, which evaluates the completeness of amino acid biosynthesis pathways based on genome annotation and sequence similarity [Supplementary Table 2]. The GapMind web tool evaluates pathways for 17 amino acids and chorismite - excluding alanine, aspartate, and glutamate - and assigns confidence levels to each pathway based on the presence of key enzymes and transporters^[35]^. Proteolytic capacity, instead, was examined by searching for peptidase-encoding genes using BLASTp against the MEROPS database^[14]^, followed by SignalP^[15]^ prediction to identify enzymes likely secreted via classical pathways [Supplementary Table 3].

Among the seven strains, *B. thetaiotaomicron* was unique in possessing complete biosynthetic pathways for all tested amino acids [Supplementary Table 2] and a large repertoire of extracellular peptidases [Supplementary Table 3], indicating efficient protein metabolism and supporting the pronounced, dose-dependent growth stimulation observed with both protein preparations. In contrast, *F. prausnitzii* exhibited multiple gaps in its biosynthetic pathways and possessed a limited set of peptidases, none of which were predicted to be secreted [Supplementary Tables 2 and 3]. These findings are consistent with its known auxotrophies and dependency on cross-fed fermentation products, e.g., acetate and amino acids, which may explain its absence of response in protein-based monocultures^[36]^. The *Bifidobacterium* spp. displayed intermediate profiles, with incomplete biosynthetic capacity and limited predicted secretion of proteolytic enzymes [Supplementary Tables 2 and 3]. Nevertheless, some strains exhibited moderate growth stimulation, possibly attributable to the uptake of small peptides or amino acids already present in the preparations, rather than active protein degradation.

Interestingly, *S. copri* exhibited a nearly complete biosynthetic profile and encoded several predicted extracellular peptidases [Supplementary Tables 2 and 3]. Nonetheless, no measurable growth was observed under any of the protein-rich conditions tested. This apparent discrepancy with our *in vitro* results may reflect a distinct ecological adaptation. *S. copri* has been consistently associated with carbohydrate-rich dietary patterns and is known to specialize in the degradation of complex plant polysaccharides rather than protein substrates^[37]^. The presence of genes encoding secreted proteases does not necessarily imply functional deployment under the tested culture conditions. It is therefore plausible that *S. copri* does not induce the expression of these enzymes in environments lacking its preferred carbohydrate sources, limiting its ability to access or metabolize legume-derived proteins in monoculture.

### Investigation of the impact of pea-derived protein ingredients on gut microbiota

To evaluate the effects of pea-derived protein ingredients on complex gut microbial communities, we performed *in vitro* cultivation assays using six stabilized microbial communities, representing key adult human gut microbiota^[16]^. Each stabilized microbial community was inoculated at a final concentration of 2% (v/v) into a IGSM and incubated for 24 h under anaerobic conditions using the MiPro model^[12]^. For each stabilized bacterial population, three experimental conditions were tested in triplicate. These included only the IGSM as a control, IGSM supplemented with 2% (w/v) of PPI, and IGSM supplemented with 2% (w/v) of PPC. The selected concentration was based on preliminary monoculture assays [Figure 1]. Furthermore, this concentration aligns with common *in vitro* cultivation protocols that recommend using substrate amounts representative of typical dietary exposure in the colon^[38]^. In fact, static batch models such as the one employed here generally operate within the range of 1 to 2% (w/v), which corresponds to a plausible proportion of protein ingredients reaching the distal intestine under physiological conditions^[38]^.

After 24 h of anaerobic cultivation, DNA was extracted from each replicate sample and processed using shallow shotgun metagenomic sequencing [Supplementary Table 4]. This approach enabled a detailed investigation of the effects of protein supplementation on microbial diversity and taxonomic composition of the stabilized microbial communities.

**Figure 2:**
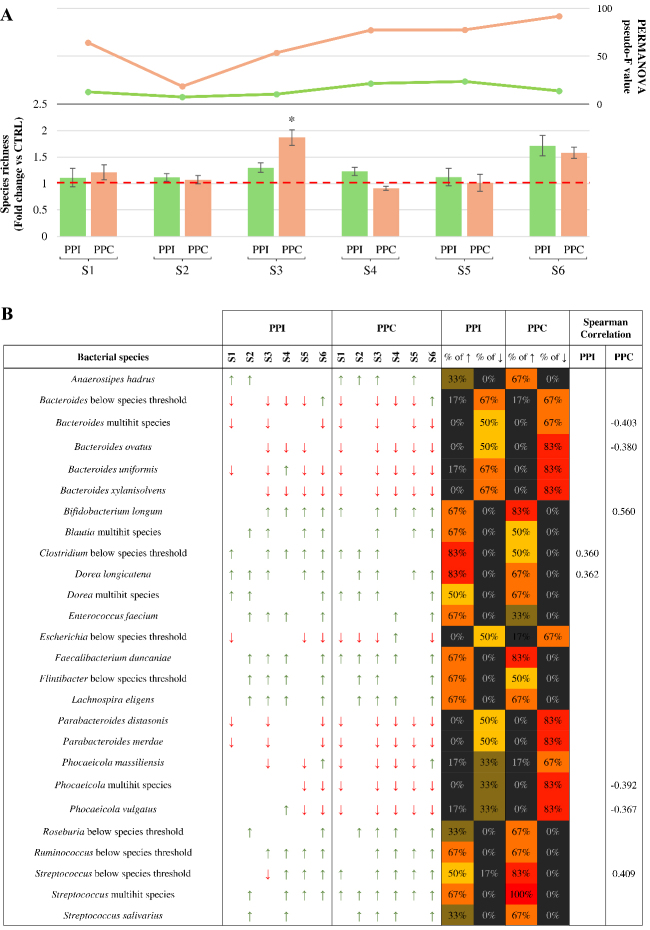
Microbiota-level response to pea-derived protein preparations. (A) Fold change in microbial species richness after 24 h of anaerobic cultivation with PPI (green bars) or PPC (orange bars), compared to the control. The red dashed line marks the fold change value of 1, indicating no variation relative to the control. Error bars represent propagated standard deviations across biological replicates. The two-line graphs above the bars represent the pseudo-F values obtained by PERMANOVA analysis, comparing the overall microbial community structure between each protein preparation and its respective control for each microbiota sample; (B) All bacterial species that showed a relevant change in abundance following treatment. Arrows indicate directionality of variation, with red downward arrows for a fold change below 0.5 and green upward arrows for a fold change above 2. Colored bars on the right indicate the percentage of microbiota samples in which each taxon showed an increase (↑) or decrease (↓) in relative abundance following supplementation to PPI or PPC. Cell colors follow a gradient from black (lowest prevalence) to red (highest prevalence). The final column displays the significant Spearman correlations (*P*-value < 0.05) between taxon abundance and protein supplementation across the six microbiota samples. PPC: Pea protein concentrate; PPI: pea protein isolate; S1-S6: samples 1-6; PERMANOVA: permutational multivariate analysis of variance; peseudo-F: the pseudo-F statistic from PERMANOVA analysis.

Focusing on microbial richness, the analysis revealed limited intra-sample variation across the 6 stabilized microbial communities tested (samples 1-6). In general, both PPI and PPC induced only modest changes in the bacterial richness compared to the control, except for S3 and S6, where supplementation led to a noticeable increase in the microbial load increase in the microbial load [Figure 2A]. These observations suggest that protein supplementation alone does not drastically alter the number of detectable taxa, although certain microbial communities may be more responsive than others.

In contrast, when considering compositional shifts, Bray-Curtis dissimilarity analysis revealed more substantial effects of protein supplementation. The magnitude of these shifts was quantified using the pseudo-F statistic [Figure 2A]. Notably, PPC seemed to induce a more pronounced modification of the microbial community than PPI, resulting in greater dissimilarity from the control in all six cases. This could indicate that the concentrate formulation exerts a broader influence on community composition, possibly by altering cross-feeding dynamics or favoring specific metabolic associations^[39,40]^.

These findings suggest that while pea-derived protein ingredients have a limited effect on richness, they do modulate microbiota structure, particularly when provided as a concentrate. Such changes may reflect selective stimulation of specific taxa and microbial interactions, with potential implications on the functions exerted by the human gut ecosystem and on the host health^[41]^.

### Species-level microbial responses to pea-derived protein ingredients

To assess how individual bacterial taxa responded to the protein-based ingredients, species-level relative abundance profiles across the six microbial communities were analyzed [Supplementary Table 5]. Changes in abundance between treatment and control for each taxon were evaluated for directionality, and only those showing a fold change of at least 2 (increase) or of 0.5 or less (decrease) were retained for further analyses [Figure 2B]. Among these, we further selected microbial taxa that exhibited a consistent trend, either increase or decrease, in at least 50 percent of the tested microbiota samples for either PPI or PPC [Figure 2B]. This dual filtering strategy was adopted to select bacterial species showing both biologically relevant modulation and inter-individual consistency in their response to the protein-based ingredients. Given the limited number of samples and the inherent inter-individual variability of the human-derived microbiota, standard inferential statistics were not applied at the species level. Instead, a descriptive approach was used to identify reproducible microbial shifts potentially associated with protein supplementation. Focusing on species that showed at least one relevant variation in the abundance in response to treatment, either under PPI or PPC exposure compared to control, a total of 26 responsive microbial taxa were identified [Figure 2B]. The analysis of species-level variation revealed that both protein preparations induced broadly similar effects on the gut microbial composition. However, certain taxa displayed differential prevalence of response depending on the protein supplementation. Notably, members of the genera *Bacteroides*, *Parabacteroides,* and *Phocaeicola* showed a consistent trend of decreased abundance across most samples under both PPI and PPC exposure. However, the decrease appeared more pronounced and frequent in the PPC condition [Figure 2B], suggesting a possible selective pressure potentially exerted by the protein concentrate^[42,43]^. Conversely, positive responses were observed for many different species [Figure 2B], including putative beneficial taxa such as *B. longum* and *Faecalibacterium duncaniae* (*F. duncaniae*), particularly following PPC supplementation.

To support these observations, Spearman correlation analysis was performed between protein supplementation and species abundance across the six stabilized microbial communities. In detail, the statistical analysis revealed that PPC was associated with a greater number of significant correlations, four negatives and two positives, compared to PPI, which showed only two positive associations, thus supporting a broader and possibly more selective modulatory potential of the concentrate. Interestingly, several *Bacteroides* species showed strong negative correlations under PPC, confirming the pattern already observed in the prevalence analysis. These results may appear in contrast with monoculture assays, in which a specific *Bacteroides* species, i.e., *B. thetaiotaomicron*, responded positively to protein supplementation. This apparent discrepancy likely reflects the complex ecological interactions occurring within microbial consortia, where the community context and metabolic cross-feeding dynamics can substantially influence individual growth responses^[39]^. Although *Bacteroides* species showed enhanced growth in monoculture assays, and *in silico* predictions highlighted their extensive proteolytic machinery and complete biosynthetic capacity, their relative abundance decreased in complex communities, particularly under PPC supplementation. This shift may be the result of interspecies competition or indirect ecological effects, where other microbes, potentially stimulated by the PPC, limited the expansion of *Bacteroides* through substrate depletion or cross-feeding-driven network changes^[39]^.

### Associations between macronutritional composition and microbial responses

The two preparations differ not only in their protein content but also in other nutritional components that may influence microbial dynamics. In fact, the two pea protein preparations tested in this study showed clear differences in their macronutritional profiles [Table 1]. PPC contained higher amounts of carbohydrates and fiber, whereas PPI was richer in protein, fat, and salt. Such compositional differences may provide a nutritional explanation for the distinct microbial responses observed in batch culture experiments. To investigate this possibility, we performed exploratory correlation analyses between species-level relative abundances and nutrient values [Supplementary Table 6].

This preliminary analysis revealed biologically consistent associations. *B. longum* and *F. duncaniae* were positively associated with carbohydrate and fiber levels, consistent with their more pronounced enrichment under PPC supplementation. Conversely, several bacteria, such as *Clostridium* and *Dorea longicatena*, displayed significant positive correlations with protein levels, supporting their greater increases under PPI. Furthermore, multiple *Bacteroides* and *Phocaeicola* species correlated negatively with carbohydrate and fiber content, mirroring the decreases already observed in PPC- and PPI-supplemented communities [Figure 2B].

Taken together, these results reinforce the notion that the different nutritional compositions of PPI and PPC can contribute to shaping distinct microbial trajectories. Although the correlations should be regarded as exploratory, given that nutrient values are constant within each formulation, they provide complementary evidence supporting both our taxonomic analyses and current knowledge from the literature. From a broader perspective, these findings suggest that even subtle differences in the processing of plant protein ingredients, leading to modest variations in macronutrient content, may influence gut microbiota responses. Further studies will be needed to confirm these trends and to clarify their potential implications for dietary applications.

## DISCUSSION

In this preliminary study, we investigated the effects of two commercially available protein-rich formulations derived from yellow peas, a PPI and a PPC, which were produced through distinct processing technologies, wet extraction and dry fractionation for PPI and PPC, respectively. These technological differences result in distinct macronutrient profiles and potentially different effects on the human gut microbiota. A combined approach employing monoculture assays and complex *in vitro* cultivation models with stabilized human gut microbiota was used to investigate both the selective growth response of individual microbial taxa and the broader community-level impact of the tested ingredients.

Our findings demonstrate that both PPI and PPC can modulate microbial composition, albeit in a taxon- and matrix-specific manner. Monoculture assays combined with *in silico* genome analyses revealed a general stimulation of growth in protein-responsive taxa such as *B. thetaiotaomicron* and *Bifidobacterium* spp., while *F. prausnitzii* and *S. copri* showed no measurable changes. *In vitro* cultivation of complex microbiota showed slightly more shifts in diversity and species abundance, with PPC consistently exerting a stronger impact on community structure and variability than PPI. Interestingly, both preparations exhibited similar directional effects on specific bacterial groups, promoting the growth of potentially beneficial taxa such as *B. longum* and *F. duncaniae*, and reducing the relative abundance of several *Bacteroides*, *Parabacteroides*, and *Phocaeicola* species. These observations suggest a shared core activity, possibly linked to nitrogen metabolism, although this hypothesis warrants further investigation into its mechanistic basis.

Comparable responses have also been reported in studies investigating other dietary proteins and prebiotic substrates. Soybean proteins were shown to increase the abundance of *Bifidobacterium* and butyrate-producing taxa while reducing *Bacteroides* spp.^[43]^. Lentil proteins have been associated with bifidogenic effects and enrichment of short-chain fatty acid producers^[2]^, while lupin-derived proteins have been reported to enhance saccharolytic bacteria due to their combined nitrogen and fiber contributions^[11]^. Beyond proteins, prebiotic carbohydrates, such as inulin, have well-established bifidogenic effects and promote *Faecalibacterium* enrichment^[27,44]^, whereas resistant starch promotes butyrate producers and limits the expansion of *Bacteroides*^[40]^. These convergent patterns suggest that pea proteins, particularly those enriched in fiber and carbohydrates, may exert ecological effects similar to those triggered by other plant proteins or even classical prebiotic substrates, reinforcing their potential as functional dietary ingredients.

An apparent divergence emerged between monoculture and community experiments. While *B. thetaiotaomicron* thrived in protein-rich monocultures, its abundance decreased in complex community cultures. This discrepancy likely reflects cross-feeding relationships and competitive dynamics, whereby proteolytic activity releases peptides and amino acids that are subsequently exploited by other taxa, limiting the expansion of the initial degrader^[34,39]^. Similar mechanisms have been described in minimal *in vitro* microbiota models^[40]^, emphasizing the importance of ecological context in shaping microbial responses to dietary proteins.

The nutritional composition of PPI and PPC provides a plausible explanation for their differential impact. PPC, obtained by dry fractionation, retained higher levels of carbohydrates and fiber, likely supporting both proteolytic and saccharolytic networks and thereby amplifying its ecological effects. In contrast, PPI, with higher protein purity but lower carbohydrate content, showed a more restricted impact on overall community composition. Even modest differences in macronutrient content may thus result in distinct microbial trajectories, highlighting the importance of processing methods when evaluating dietary proteins^[41,42]^.

To date, only a few studies have directly compared the microbiota responses to plant protein ingredients obtained through different processing technologies. The present work, therefore, provides one of the first comparative evaluations of PPI and PPC, offering an initial insight into how technological processing and compositional features may influence gut microbial communities. Moreover, the experimental setup employed, based on stabilized human gut microbiota, provides a relevant and controlled system to evaluate the microbiota-modulating properties of food ingredients under *in vitro* conditions. To our knowledge, this study is among the first to characterize in parallel the responses of both synthetic and complex human gut microbial communities to pea-derived protein preparations, providing novel insights into their potential microbiota-targeted functionality.

Although this preliminary study was specifically designed as an exploratory assessment of the microbiota-modulating potential of pea-derived protein ingredients, some limitations should be acknowledged. The relatively small number of microbiota samples analyzed limits the range of inter-individual variability that can be captured. Additionally, while taxonomic shifts were observed, the functional consequences of these changes remain to be clarified. The 24-h incubation may be considered a limitation, as it does not allow for the assessment of ecological resilience or long-term stability. However, this time frame is widely adopted in batch fermentation models to capture early microbial responses to dietary substrates while minimizing artifacts related to substrate depletion and medium acidification^[38]^. Future work should therefore build on this pilot framework by including a larger panel of donor-derived microbial communities and by exploring the effects of these ingredients under physiological conditions through *in vivo* studies or dietary interventions. Functional consequences remain speculative, as metatranscriptomics, metabolomics, or gnotobiotic validation were not feasible in this setting. Moreover, nutritional characterization was limited to the macronutrient data provided by the manufacturer, without detailed profiling of non-protein bioactive components. These considerations underline the exploratory nature of the present work, which was primarily intended to provide a proof-of-concept and generate hypotheses for future validation.

In summary, our results suggest that pea-derived protein ingredients, especially when processed as concentrates, may act as microbiota-modulating agents with selective effects on specific human gut microbes. This work provides one of the first comparative assessments of PPI and PPC obtained via different processing technologies, representing a preliminary step toward understanding how such variations could inform the rational design of functional foods or personalized nutrition strategies aimed at promoting beneficial microbial functions in the gut ecosystem.

## DECLARATIONS

### Availability of data and materials

Raw shallow shotgun sequencing data have been deposited in the Sequence Read Archive (SRA) under accession number PRJNA1302152.

### Copyright

© The Author(s) 2025.
