# Supplementary material for: Disclosing the effects of pea-derived proteins on the human gut microbiota
Source: Microbiome Res Rep. 2025 Nov 25;4(4):42. doi: 10.20517/mrr.2025.77 (PMC12702649; doi:10.20517/mrr.2025.77)
Supplement: Supplementary file 1 [file mrr-4-4-42-SupplementaryMaterials.pdf]

Supplementary Materials

Disclosing the effects of pea-derived proteins on the human gut microbiota

Leonardo Mancabelli[1,2], Christian Milani[2,3], Giulia Longhi[3], Gabriele Andrea Lugli[2,3], Chiara Tarracchini[3], Francesca Turroni[2,3], Marco Ventura[2,3]

[1]Department of Medicine and Surgery, University of Parma, Parma 43125, Italy.
[2]Interdepartmental Research Centre "Microbiome Research Hub", University of Parma, Parma 43124, Italy.
[3]Laboratory of Probiogenomics, Department of Chemistry, Life Sciences and Environmental Sustainability, University of Parma, Parma 43124, Italy.

Correspondence to: Prof. Leonardo Mancabelli, Department of Medicine and Surgery, University of Parma, Parma 43125, Italy. E-mail: leonardo.mancabelli@unipr.it

ORCID: Leonardo Mancabelli (0000-0002-1744-2214)

Supplementary Table 1. Bacterial growth (cell count) of each strain under different conditions as assessed by flow cytometry

Species	Sample			Counts
Bifidobacterium breve LMG13208	**Control**	**-**	**A**	1.20E+09
	**Control**	**-**	**B**	9.74E+08
	**Control**	**-**	**C**	1.02E+09
	**PPI**	**0.50%**	**A**	1.25E+09
	**PPI**	**0.50%**	**B**	1.04E+09
	**PPI**	**0.50%**	**C**	8.72E+08
	**PPI**	**1%**	**A**	2.35E+09
	**PPI**	**1%**	**B**	2.01E+09
	**PPI**	**1%**	**C**	1.74E+09
	**PPI**	**2%**	**A**	2.85E+09
	**PPI**	**2%**	**B**	1.97E+09
	**PPI**	**2%**	**C**	2.67E+09
	**PPC**	**0.50%**	**A**	1.31E+09
	**PPC**	**0.50%**	**B**	1.66E+09
	**PPC**	**0.50%**	**C**	1.38E+09
	**PPC**	**1%**	**A**	1.54E+09
	**PPC**	**1%**	**B**	1.43E+09
	**PPC**	**1%**	**C**	1.47E+09
	**PPC**	**2%**	**A**	1.64E+09
	**PPC**	**2%**	**B**	1.97E+09

	**PPC**	**2%**	**C**	1.53E+09
***Bifidobacterium bifidum* LMG 11041**	**Control**	**-**	**A**	9.76E+07
	**Control**	**-**	**B**	1.04E+08
	**Control**	**-**	**C**	1.22E+08
	**PPI**	**0.50%**	**A**	1.68E+08
	**PPI**	**0.50%**	**B**	1.90E+08
	**PPI**	**0.50%**	**C**	1.82E+08
	**PPI**	**1%**	**A**	2.08E+08
	**PPI**	**1%**	**B**	2.17E+08
	**PPI**	**1%**	**C**	1.64E+08
	**PPI**	**2%**	**A**	2.28E+08
	**PPI**	**2%**	**B**	2.35E+08
	**PPI**	**2%**	**C**	1.94E+08
	**PPC**	**0.50%**	**A**	2.19E+08
	**PPC**	**0.50%**	**B**	2.33E+08
	**PPC**	**0.50%**	**C**	2.34E+08
	**PPC**	**1%**	**A**	2.02E+08
	**PPC**	**1%**	**B**	2.06E+08
	**PPC**	**1%**	**C**	1.84E+08
	**PPC**	**2%**	**A**	1.71E+08
	**PPC**	**2%**	**B**	2.47E+08
	**PPC**	**2%**	**C**	2.19E+08
***Bifidobacterium adolescentis* ATCC 15703**	**Control**	**-**	**A**	2.98E+08
	**Control**	**-**	**B**	1.17E+08
	**Control**	**-**	**C**	2.21E+08
	**PPI**	**0.50%**	**A**	7.90E+08
	**PPI**	**0.50%**	**B**	7.93E+08
	**PPI**	**0.50%**	**C**	8.53E+07
	**PPI**	**1%**	**A**	8.86E+08
	**PPI**	**1%**	**B**	8.59E+08
	**PPI**	**1%**	**C**	9.35E+08
	**PPI**	**2%**	**A**	1.44E+09
	**PPI**	**2%**	**B**	1.37E+09
	**PPI**	**2%**	**C**	1.35E+09
	**PPC**	**0.50%**	**A**	6.13E+08
	**PPC**	**0.50%**	**B**	6.27E+08
	**PPC**	**0.50%**	**C**	7.62E+08
	**PPC**	**1%**	**A**	8.90E+08
	**PPC**	**1%**	**B**	9.71E+08
	**PPC**	**1%**	**C**	1.00E+09
	**PPC**	**2%**	**A**	1.46E+09
	**PPC**	**2%**	**B**	1.59E+09
	**PPC**	**2%**	**C**	1.66E+09
***Bifidobacterium longum* subsp. *longum* LMG 13197**	**Control**	**-**	**A**	8.12E+07
	**Control**	**-**	**B**	1.89E+08
	**Control**	**-**	**C**	1.79E+08
	**PPI**	**0.50%**	**A**	9.77E+07
	**PPI**	**0.50%**	**B**	2.54E+08
	**PPI**	**0.50%**	**C**	2.94E+08
	**PPI**	**1%**	**A**	8.26E+08
	**PPI**	**1%**	**B**	9.25E+08
	**PPI**	**1%**	**C**	9.16E+08
	**PPI**	**2%**	**A**	1.33E+09
	**PPI**	**2%**	**B**	1.36E+09
	**PPI**	**2%**	**C**	1.22E+09
	**PPC**	**0.50%**	**A**	8.53E+08
	**PPC**	**0.50%**	**B**	8.15E+08
	**PPC**	**0.50%**	**C**	7.53E+08
	**PPC**	**1%**	**A**	9.61E+08

	**PPC**	**1%**	**B**	1.17E+09
	**PPC**	**1%**	**C**	1.16E+09
	**PPC**	**2%**	**A**	1.57E+09
	**PPC**	**2%**	**B**	1.33E+09
	**PPC**	**2%**	**C**	1.42E+09
***Bacteroides tethaiotaomicron* 41F**	**Control**	**-**	**A**	5.46E+08
	**Control**	**-**	**B**	5.55E+08
	**Control**	**-**	**C**	5.93E+08
	**PPI**	**0.50%**	**A**	5.78E+09
	**PPI**	**0.50%**	**B**	5.87E+09
	**PPI**	**0.50%**	**C**	5.49E+09
	**PPI**	**1%**	**A**	4.53E+09
	**PPI**	**1%**	**B**	4.74E+09
	**PPI**	**1%**	**C**	4.22E+09
	**PPI**	**2%**	**A**	4.90E+09
	**PPI**	**2%**	**B**	6.24E+09
	**PPI**	**2%**	**C**	5.60E+09
	**PPC**	**0.50%**	**A**	2.47E+09
	**PPC**	**0.50%**	**B**	2.55E+09
	**PPC**	**0.50%**	**C**	3.50E+09
	**PPC**	**1%**	**A**	4.17E+09
	**PPC**	**1%**	**B**	4.13E+09
	**PPC**	**1%**	**C**	3.59E+09
	**PPC**	**2%**	**A**	4.33E+09
	**PPC**	**2%**	**B**	4.61E+09
	**PPC**	**2%**	**C**	4.55E+09
***Segatella copri* DSM 18205**	**Control**	**-**	**A**	2.63E+07
	**Control**	**-**	**B**	2.58E+07
	**Control**	**-**	**C**	2.30E+07
	**PPI**	**0.50%**	**A**	2.52E+07
	**PPI**	**0.50%**	**B**	2.48E+07
	**PPI**	**0.50%**	**C**	2.54E+07
	**PPI**	**1%**	**A**	2.82E+07
	**PPI**	**1%**	**B**	2.69E+07
	**PPI**	**1%**	**C**	3.11E+07
	**PPI**	**2%**	**A**	2.26E+07
	**PPI**	**2%**	**B**	2.58E+07
	**PPI**	**2%**	**C**	2.98E+07
	**PPC**	**0.50%**	**A**	2.93E+07
	**PPC**	**0.50%**	**B**	2.71E+07
	**PPC**	**0.50%**	**C**	2.66E+07
	**PPC**	**1%**	**A**	2.68E+07
	**PPC**	**1%**	**B**	2.20E+07
	**PPC**	**1%**	**C**	2.25E+07
	**PPC**	**2%**	**A**	2.62E+07
	**PPC**	**2%**	**B**	2.76E+07
	**PPC**	**2%**	**C**	2.15E+07
***Faecalibacterium prausnitzii* DSM 176677**	**Control**	**-**	**A**	3.31E+07
	**Control**	**-**	**B**	2.61E+07
	**Control**	**-**	**C**	2.54E+07
	**PPI**	**0.50%**	**A**	2.92E+07
	**PPI**	**0.50%**	**B**	3.03E+07
	**PPI**	**0.50%**	**C**	3.20E+07
	**PPI**	**1%**	**A**	2.93E+07
	**PPI**	**1%**	**B**	2.77E+07
	**PPI**	**1%**	**C**	2.23E+07
	**PPI**	**2%**	**A**	2.68E+07
	**PPI**	**2%**	**B**	2.56E+07
	**PPI**	**2%**	**C**	3.46E+07

	**PPC**	**0.50%**	**A**	3.44E+07
	**PPC**	**0.50%**	**B**	3.29E+07
	**PPC**	**0.50%**	**C**	2.91E+07
	**PPC**	**1%**	**A**	3.57E+07
	**PPC**	**1%**	**B**	2.92E+07
	**PPC**	**1%**	**C**	3.22E+07
	**PPC**	**2%**	**A**	2.80E+07
	**PPC**	**2%**	**B**	2.96E+07
	**PPC**	**2%**	**C**	2.31E+07

Supplementary Table 2. Predicted amino acid biosynthesis pathways per strain using GapMind. Confidence levels indicate pathway completeness: high, medium, or low

Pathway	*Bacteroides thetaiotaomicron* 41F	*Bifidobacterium adolescentis* ATCC 15703	*Bifidobacterium bifidum* LMG 11041	*Bifidobacterium breve* LMG 13208	*Bifidobacterium longum* subsp. *Longum* LMG 13197	*Faecalibacterium prausnitzii* DSM 17677	*Segatella copri* DSM 18205
**arg**	high confidence	high confidence	high confidence	high confidence	high confidence	high confidence	high confidence
**asn**	high confidence	high confidence	medium confidence	high confidence	high confidence	high confidence	high confidence
**chorismate**	high confidence	medium confidence	medium confidence	medium confidence	medium confidence	medium confidence	high confidence
**cys**	high confidence	high confidence	medium confidence	medium confidence	high confidence	high confidence	high confidence
**gln**	high confidence	high confidence	high confidence	high confidence	high confidence	high confidence	high confidence
**gly**	high confidence	high confidence	high confidence	high confidence	high confidence	high confidence	high confidence
**his**	high confidence	medium confidence	medium confidence	medium confidence	low confidence	low confidence	high confidence
**ile**	high confidence	high confidence	high confidence	low confidence	high confidence	high confidence	high confidence
**leu**	high confidence	high confidence	high confidence	high confidence	high confidence	high confidence	high confidence
**lys**	high confidence	medium confidence	low confidence	low confidence	low confidence	medium confidence	high confidence
**met**	high confidence	high confidence	high confidence	high confidence	high confidence	low confidence	high confidence
**phe**	high confidence	low confidence	low confidence	low confidence	low confidence	medium confidence	high confidence
**pro**	high confidence	high confidence	high confidence	high confidence	high confidence	medium confidence	low confidence
**ser**	high confidence	high confidence	high confidence	high confidence	high confidence	low confidence	medium confidence
**thr**	high confidence	high confidence	high confidence	low confidence	medium confidence	medium confidence	high confidence
**trp**	high confidence	high confidence	medium confidence	high confidence	high confidence	low confidence	high confidence
**tyr**	high confidence	medium confidence	medium confidence	medium confidence	medium confidence	medium confidence	high confidence

val	high confidence	high confidence	high confidence	high confidence	high confidence	high confidence	high confidence

Supplementary Table 3. Predicted secretory peptidases identified via MEROPS BLASTp (E-value < 1e-30) and SignalP v6.0 classification

				Prediction	OTHER	SP(Sec/SPI)	LIPO(Sec/SPII)	TAT(Tat/SPI)	TATLIPO(Tat/SPII)	PILIN(Sec/SPIII)
***Bifidobacterium adolescentis* ATCC 15703**		C26	MER0437468	OTHER	1	0	0	0	0	0
		C26	MER0045886	OTHER	1	0	0	0	0	0
		C26	MER0065588	OTHER	1	0	0	0	0	0
	Possible implication in pea protein degradation	S09B	MER0195666	OTHER	1	0	0	0	0	0
		S01C	MER0004673	OTHER	1	0	0	0	0	0
		C69	MER0002163	OTHER	1	0	0	0	0	0
		C60A	MER0038408	OTHER	0.99988	0.00011	0	0	0	0
		C69	MER0002163	OTHER	1	0	0	0	0	0
	Possible implication in pea protein degradation	C01B	MER0002481	OTHER	1	0	0	0	0	0
	Possible implication in pea protein degradation	S09X	MER0030934	OTHER	1	0	0	0	0	0
		S16	MER0014135	OTHER	1	0	0	0	0	0
		M41	MER0015199	OTHER	1	0	0	0	0	0
	Possible implication in pea protein degradation	S09X	MER0030934	OTHER	0.99999	0	0	0	0	0
	Possible implication in pea protein degradation	S09X	MER0031562	OTHER	1	0	0	0	0	0
	Possible implication in pea protein degradation	M13	MER0004775	OTHER	1	0	0	0	0	0
		M24A	MER0001243	OTHER	1	0	0	0	0	0
		C44	MER0003327	OTHER	1	0	0	0	0	0
		C44	MER0011806	OTHER	1	0	0	0	0	0
		C26	MER0060647	OTHER	1	0	0	0	0	0

		U72	MER0397777	OTHER	1	0	0	0	0	0
		U72	MER0397777	OTHER	1	0	0	0	0	0
		S14	MER0004857	OTHER	1	0	0	0	0	0
		S14	MER0004847	OTHER	1	0	0	0	0	0
		S33	MER0001367	OTHER	1	0	0	0	0	0
		C59	MER0019881	OTHER	1	0	0	0	0	0
		T05	MER0011830	OTHER	1	0	0	0	0	0
		M20F	MER0026494	OTHER	1	0	0	0	0	0
	Possible implication in pea protein degradation	C01B	MER0000728	OTHER	0.99999	0	0	0	0	0
		M20D	MER0002655	OTHER	1	0	0	0	0	0
		C44	MER0034539	OTHER	1	0	0	0	0	0
		M01	MER0001003	OTHER	1	0	0	0	0	0
		M38	MER0066192	OTHER	1	0	0	0	0	0
		S26A	MER0004537	OTHER	1	0	0	0	0	0
		T02	MER0003338	OTHER	1	0	0	0	0	0
		U32	MER0003855	OTHER	1	0	0	0	0	0
		C26	MER0045094	OTHER	1	0	0	0	0	0
		C15	MER0001424	OTHER	1	0	0	0	0	0
		C44	MER0198917	OTHER	1	0	0	0	0	0
		M24B	MER0028601	OTHER	1	0	0	0	0	0
	Possible implication in pea protein degradation	S09X	MER0031562	OTHER	1	0	0	0	0	0
		M48B	MER0006293	OTHER	1	0	0	0	0	0
		C26	MER0066916	OTHER	1	0	0	0	0	0
		S09A	MER0000410	OTHER	1	0	0	0	0	0
		C60A	MER0038408	OTHER	0.99996	0	0	0	0	0
		C60A	MER0038408	OTHER	0.98132	0.01865	7E-06	5E-06	6E-06	5E-06
***Bifidobacterium bifidum* LMG 11041**		C26	MER0045094	OTHER	0.99999	0	0	0	0	0
	Possible implication in pea protein degradation	S09B	MER0195666	OTHER	1	0	0	0	0	0
		C26	MER0065588	OTHER	1	0	0	0	0	0
		T05	MER0011830	OTHER	1	0	0	0	0	0

	C44	MER0011806	OTHER	1	0	0	0	0	0
	C15	MER0001424	OTHER	1	0	0	0	0	0
	M41	MER0015199	OTHER	1	0	0	0	0	0
	M24A	MER0001243	OTHER	1	0	0	0	0	0
	C26	MER0060647	OTHER	1	0	0	0	0	0
	C69	MER0002163	OTHER	1	0	0	0	0	0
	C59	MER0019881	OTHER	0.99999	0	0	0	0	0
	S14	MER0004847	OTHER	1	0	0	0	0	0
	S33	MER0001367	OTHER	1	0	0	0	0	0
	C60A	MER0038408	OTHER	0.99688	0.00311	2E-06	2E-06	1E-06	1E-06
	U32	MER0003855	OTHER	1	0	0	0	0	0
	C69	MER0002163	OTHER	1	0	0	0	0	0
Possible implication in pea protein degradation	C01B	MER0000728	OTHER	1	0	0	0	0	0
	M48B	MER0006293	OTHER	0.99999	0	0	0	0	0
	S16	MER0014135	OTHER	1	0	0	0	0	0
	M20D	MER0002655	OTHER	1	0	0	0	0	0
Possible implication in pea protein degradation	S09X	MER0031562	OTHER	1	0	0	0	0	0
	C15	MER0001424	OTHER	1	0	0	0	0	0
	C60A	MER0038408	OTHER	0.99997	0	0	0	0	0
	C26	MER0066916	OTHER	1	0	0	0	0	0
	S09A	MER0000410	OTHER	1	0	0	0	0	0
	M01	MER0001003	OTHER	1	0	0	0	0	0
Possible implication in pea protein degradation	M13	MER0004775	OTHER	0.99999	0	0	0	0	0
	M24B	MER0028601	OTHER	1	0	0	0	0	0
	U72	MER0397777	OTHER	1	0	0	0	0	0
Possible implication in pea protein degradation	C01B	MER0002481	OTHER	1	0	0	0	0	0
	C26	MER0045886	OTHER	1	0	0	0	0	0
	S01C	MER0004673	OTHER	1	0	0	0	0	0
	T02	MER0003338	OTHER	1	0	0	0	0	0
	S14	MER0004857	OTHER	1	0	0	0	0	0
	C26	MER0437468	OTHER	1	0	0	0	0	0

		M20F	MER0026494	OTHER	1	0	0	0	0	0
		U72	MER0397777	OTHER	1	0	0	0	0	0
		C44	MER0198917	OTHER	1	0	0	0	0	0
		S26A	MER0004537	OTHER	1	0	0	0	0	0
		C60A	MER0038408	OTHER	1	0	0	0	0	0
	Possible implication in pea protein degradation	S08A	MER0002057	OTHER	1	0	0	0	0	0
***Bifidobacterium breve* LMG 13208**		C26	MER0045094	OTHER	1	0	0	0	0	0
	Possible implication in pea protein degradation	S09B	MER0195666	OTHER	1	0	0	0	0	0
		M48B	MER0006293	OTHER	0.99999	0	0	0	0	0
		C60A	MER0038408	OTHER	0.99395	0.00608	2E-06	2E-06	2E-06	1E-06
		C60A	MER0038408	OTHER	1	0	0	0	0	0
		C69	MER0002163	OTHER	1	0	0	0	0	0
		C69	MER0002163	OTHER	1	0	0	0	0	0
		M01	MER0001003	OTHER	1	0	0	0	0	0
		C44	MER0003327	OTHER	1	0	0	0	0	0
		M20F	MER0026494	OTHER	1	0	0	0	0	0
		T05	MER0011830	OTHER	0.99999	0	0	0	0	0
		C26	MER0045886	OTHER	1	0	0	0	0	0
		M20D	MER0002655	OTHER	1	0	0	0	0	0
		S14	MER0004847	OTHER	1	0	0	0	0	0
		M38	MER0066192	OTHER	1	0	0	0	0	0
		C26	MER0437468	OTHER	1	0	0	0	0	0
		C15	MER0001424	OTHER	1	0	0	0	0	0
	Possible implication in pea protein degradation	C01B	MER0002481	OTHER	1	0	0	0	0	0
		M41	MER0015199	OTHER	1	0	0	0	0	0
	Possible implication in pea protein degradation	M13	MER0004775	OTHER	1	0	0	0	0	0
		U72	MER0397777	OTHER	1	0	0	0	0	0
		I04	MER0021383	OTHER	1	0	0	0	0	0
		C26	MER0065588	OTHER	1	0	0	0	0	0
		M38	MER0066192	OTHER	1	0	0	0	0	0

		S14	MER0004857	OTHER	1	0	0	0	0	0
		M24B	MER0028601	OTHER	1	0	0	0	0	0
		C59	MER0019881	OTHER	1	0	0	0	0	0
		C26	MER0060647	OTHER	1	0	0	0	0	0
		C26	MER0066916	OTHER	1	0	0	0	0	0
		U32	MER0003855	OTHER	1	0	0	0	0	0
		S01C	MER0004673	OTHER	1	0	0	0	0	0
		C44	MER0011806	OTHER	1	0	0	0	0	0
		S16	MER0014135	OTHER	1	0	0	0	0	0
		U72	MER0397777	OTHER	1	0	0	0	0	0
		S09A	MER0000410	OTHER	1	0	0	0	0	0
		S33	MER0001367	OTHER	1	0	0	0	0	0
		S26A	MER0004537	OTHER	0.99998	0	0	0	0	0
***Faecalibacterium prausnitzii* DSM 17677**		S01C	MER0004673	OTHER	0.99999	0	0	0	0	0
		M20A	MER0001361	OTHER	1	0	0	0	0	0
		M38	MER0066192	OTHER	1	0	0	0	0	0
		T05	MER0011829	OTHER	1	0	0	0	0	0
		M18	MER0015303	OTHER	1	0	0	0	0	0
		C26	MER0060647	OTHER	1	0	0	0	0	0
		M38	MER0005767	OTHER	1	0	0	0	0	0
		M20A	MER0001361	OTHER	1	0	0	0	0	0
		C26	MER0065588	OTHER	1	0	0	0	0	0
		M19	MER0013425	OTHER	1	0	0	0	0	0
		C40	MER0001322	OTHER	1	0	0	0	0	0
		C26	MER0045886	OTHER	1	0	0	0	0	0
		C40	MER0003807	OTHER	1	0	0	0	0	0
		M41	MER0005466	OTHER	1	0	0	0	0	0
	Possible implication in pea protein degradation	S09X	MER0030934	OTHER	1	0	0	0	0	0
		M41	MER0002602	OTHER	0.99999	0	0	0	0	0
	Possible implication in pea protein degradation	S09X	MER0030934	OTHER	1	0	0	0	0	0

	U32	MER0013876	OTHER	1	0	0	0	0	0
Possible implication in pea protein degradation	C01B	MER0003535	OTHER	1	0	0	0	0	0
	M18	MER0005460	OTHER	1	0	0	0	0	0
	S16	MER0000485	OTHER	1	0	0	0	0	0
	M38	MER0066192	OTHER	1	0	0	0	0	0
	M20B	MER0001421	OTHER	1	0	0	0	0	0
	M29	MER0001287	OTHER	1	0	0	0	0	0
Possible implication in pea protein degradation	M16B	MER0004423	OTHER	1	0	0	0	0	0
	M24A	MER0001243	OTHER	1	0	0	0	0	0
	S14	MER0011947	OTHER	1	0	0	0	0	0
	M32	MER0002064	OTHER	1	0	0	0	0	0
	C40	MER0003807	OTHER	1	0	0	0	0	0
	C59	MER0019881	OTHER	1	0	0	0	0	0
	A25	MER0001292	OTHER	1	0	0	0	0	0
	M16C	MER0014056	OTHER	0.99998	0	0	0	0	0
	S55	MER0003459	OTHER	1	0	0	0	0	0
	M20D	MER0002655	OTHER	1	0	0	0	0	0
	M03C	MER0078856	OTHER	1	0	0	0	0	0
	U32	MER0003855	OTHER	1	0	0	0	0	0
	C69	MER0002163	OTHER	1	0	0	0	0	0
	M24B	MER0016542	OTHER	1	0	0	0	0	0
	S16	MER0014135	OTHER	1	0	0	0	0	0
	C44	MER0011806	OTHER	1	0	0	0	0	0
	C26	MER0437468	OTHER	1	0	0	0	0	0
	S14	MER0000474	OTHER	1	0	0	0	0	0
	M50B	MER0004468	OTHER	1	0	0	0	0	0
	S11	MER0000454	OTHER	0.99999	0	0	0	0	0
	C44	MER1054487	OTHER	1	0	0	0	0	0
	C40	MER0003807	OTHER	1	0	0	0	0	0
	C44	MER0003327	OTHER	1	0	0	0	0	0
	C60B	MER0025283	OTHER	0.94849	0.05138	3.3E-05	3.9E-05	2.9E-05	2.4E-05

		S11	MER0000454	SP	0.02145	0.97745	0.00036	0.00027	0.00024	0.00022
		S11	MER0000454	SP	0.00031	0.99882	0.00027	0.00023	0.0002	0.00018
		C69	MER0016508	SP	0.00025	0.99895	0.00024	0.00022	0.00017	0.00018
		C69	MER0016508	SP	0.00025	0.99895	0.00024	0.00022	0.00017	0.00018
***Bifidobacterium longum* subsp. *longum* LMG 13197**		C44	MER0004101	OTHER	1	0	0	0	0	0
		S09A	MER0000410	OTHER	1	0	0	0	0	0
		C15	MER0001424	OTHER	1	0	0	0	0	0
		M20D	MER0002655	OTHER	1	0	0	0	0	0
		S14	MER0004857	OTHER	1	0	0	0	0	0
		C26	MER0437468	OTHER	1	0	0	0	0	0
		C59	MER0019881	OTHER	1	0	0	0	0	0
		M24A	MER0001243	OTHER	1	0	0	0	0	0
		T05	MER0011830	OTHER	0.99999	0	0	0	0	0
		M20F	MER0026494	OTHER	1	0	0	0	0	0
		M01	MER0001003	OTHER	1	0	0	0	0	0
		I04	MER0021383	OTHER	1	0	0	0	0	0
		C60A	MER0038408	OTHER	1	0	0	0	0	0
		C69	MER0002163	OTHER	1	0	0	0	0	0
	Possible implication in pea protein degradation	C01B	MER0002628	OTHER	1	0	0	0	0	0
		S16	MER0014135	OTHER	0.99999	0	0	0	0	0
	Possible implication in pea protein degradation	C01B	MER0002481	OTHER	1	0	0	0	0	0
		C26	MER0045094	OTHER	1	0	0	0	0	0
		M48B	MER0006293	OTHER	0.99999	0	0	0	0	0
		C26	MER0066916	OTHER	1	0	0	0	0	0
		S26A	MER0004537	OTHER	1	0	0	0	0	0
		M38	MER0066192	OTHER	1	0	0	0	0	0
		S33	MER0001367	OTHER	1	0	0	0	0	0
	Possible implication in pea protein degradation	S09B	MER0195666	OTHER	1	0	0	0	0	0
		U32	MER0003855	OTHER	1	0	0	0	0	0

		C44	MER0003327	OTHER	0.99999	0	0	0	0	0
		S14	MER0004847	OTHER	0.99998	0	0	0	0	0
		M20A	MER0001361	OTHER	1	0	0	0	0	0
		C44	MER0198917	OTHER	1	0	0	0	0	0
		S26A	MER0004537	OTHER	1	0	0	0	0	0
		C60A	MER0038408	OTHER	0.99998	0	0	0	0	0
		M38	MER0005900	OTHER	0.99999	0	0	0	0	0
		M24B	MER0028601	OTHER	1	0	0	0	0	0
		U72	MER0397777	OTHER	1	0	0	0	0	0
		U72	MER0397777	OTHER	1	0	0	0	0	0
	Possible implication in pea protein degradation	M13	MER0004775	OTHER	1	0	0	0	0	0
		M41	MER0015199	OTHER	0.99999	0	0	0	0	0
	Possible implication in pea protein degradation	S09X	MER0031562	OTHER	1	0	0	0	0	0
		S01C	MER0004673	OTHER	1	0	0	0	0	0
		C26	MER0045886	OTHER	1	0	0	0	0	0
		C60A	MER0038408	OTHER	0.99688	0.00311	2E-06	2E-06	1E-06	1E-06
		C26	MER0060647	OTHER	1	0	0	0	0	0
***Segatella copri* DSM 18205**		M48C	MER0026552	LIPO	0	0	1	0	0	0
		I04	MER0284643	LIPO	0	0	1	0	0	0
		S12	MER0005925	LIPO	0.48519	1.9E-05	0.51479	0	0	0
	Possible implication in pea protein degradation	S08A	MER0002575	LIPO	0	0	1	0	0	0
	Possible implication in pea protein degradation	C01A	MER0095620	LIPO	0	0	1	0	0	0
		C11X	MER0030326	LIPO	0	0	1	0	0	0
		M03A	MER0001161	OTHER	1	0	0	0	0	0
		S49A	MER0001299	OTHER	0.99999	1E-06	0	0	0	0
		C26	MER0045886	OTHER	1	0	0	0	0	0
		C26	MER0045094	OTHER	1	0	0	0	0	0
		C26	MER0065588	OTHER	1	0	0	0	0	0
		S16	MER0014135	OTHER	1	0	0	0	0	0
		M20C	MER0047723	OTHER	1	0	0	0	0	0

	U32	MER0013876	OTHER	1	0	0	0	0	0
Possible implication in pea protein degradation	M49	MER0028047	OTHER	1	0	0	0	0	0
	C26	MER0437468	OTHER	1	0	0	0	0	0
	S33	MER0000436	OTHER	1	0	0	0	0	0
	M41	MER0005496	OTHER	0.90374	8E-06	0.0963	0	0	0
	M20C	MER0047723	OTHER	1	0	0	0	0	0
	M20B	MER0001421	OTHER	1	0	0	0	0	0
	M24B	MER0001248	OTHER	1	0	0	0	0	0
	C44	MER0012158	OTHER	1	0	0	0	0	0
	S24	MER0000576	OTHER	1	0	0	0	0	0
	C26	MER0066916	OTHER	1	0	0	0	0	0
	M20A	MER0028025	OTHER	1	0	0	0	0	0
	M18	MER0003372	OTHER	1	0	0	0	0	0
	M19	MER0011343	OTHER	1	0	0	0	0	0
	U32	MER0003242	OTHER	1	0	0	0	0	0
	S14	MER0000474	OTHER	1	0	0	0	0	0
	C44	MER0198917	OTHER	1	0	0	0	0	0
	C44	MER0034539	OTHER	1	0	0	0	0	0
	C56	MER0042827	OTHER	1	0	0	0	0	0
	C44	MER0003327	OTHER	1	0	0	0	0	0
	M24B	MER0004321	OTHER	1	0	0	0	0	0
Possible implication in pea protein degradation	M16B	MER0003448	OTHER	0.99999	0	0	0	0	0
	M24A	MER0001243	OTHER	1	0	0	0	0	0
	S41A	MER0001297	OTHER	1	0	0	0	0	0
	C26	MER0078640	OTHER	1	0	0	0	0	0
	S16	MER0000485	OTHER	1	0	0	0	0	0
Possible implication in pea protein degradation	S09C	MER0066184	OTHER	1	0	0	0	0	0
Possible implication in pea protein degradation	S09X	MER0030934	OTHER	1	0	0	0	0	0
Possible implication in pea protein degradation	M13	MER0004775	SP	0.00018	0.99926	0.00016	0.00016	0.00013	0.00013
Possible implication in pea protein degradation	M13	MER0004775	SP	0.00023	0.99904	0.0002	0.0002	0.00017	0.00016

	Possible implication in pea protein degradation	S09C	MER0034615	SP	0.00017	0.99933	0.00014	0.00015	0.00012	0.00013
		M03A	MER0001158	SP	0.00019	0.99923	0.00016	0.00017	0.00013	0.00013
		C10	MER0010975	SP	0.00014	0.66612	0.33348	9.9E-05	8.8E-05	8.5E-05
	Possible implication in pea protein degradation	C01A	MER0095620	SP	0.00019	0.99923	0.00016	0.00017	0.00014	0.00014
	Possible implication in pea protein degradation	S09B	MER0001423	SP	0.00021	0.99916	0.0002	0.00018	0.00015	0.00015
		C25	MER0034582	SP	0.00022	0.99912	0.0002	0.00019	0.00014	0.00014
	Possible implication in pea protein degradation	S09X	MER0037861	SP	0.00025	0.99905	0.00021	0.0002	0.00016	0.00015
	Possible implication in pea protein degradation	M16B	MER0001233	SP	0.00017	0.99929	0.00015	0.00016	0.00013	0.00013
		S41A	MER0015684	SP	0.00022	0.99912	0.00019	0.00017	0.00014	0.00014
		S41A	MER0001425	SP	0.00019	0.99917	0.00018	0.00017	0.00014	0.00014
		S46	MER0014366	SP	0.0002	0.99913	0.00022	0.00018	0.00015	0.00015
	Possible implication in pea protein degradation	C01B	MER0000728	SP	0.0002	0.99917	0.00017	0.00017	0.00014	0.00014
		S01C	MER0013623	SP	0.00025	0.99899	0.00021	0.00021	0.00017	0.00017
	Possible implication in pea protein degradation	S09B	MER0005196	SP	0.00019	0.99923	0.00017	0.00018	0.00014	0.00014
***Bacteroides thetaiotaomicron* 41F**		S41A	MER0195376	LIPO	0	0	1	0	0	0
		M03A	MER0001158	LIPO	0	0	1	0	0	0
		M93	MER0491616	LIPO	0	0	1	0	0	0
		M93	MER0491616	LIPO	0	0	1	0	0	0
	Possible implication in pea protein degradation	M13	MER0004775	LIPO	0	0	1	0	0	0
	Possible implication in pea protein degradation	S09C	MER0066184	LIPO	0	0	1	0	0	0
		I39	MER0034541	LIPO	0	1E-06	1	0	0	0
	Possible implication in pea protein degradation	S09C	MER0034615	LIPO	0	0	1	0	0	0
		C11X	MER0028004	LIPO	0.33491	8.5E-05	0.66498	0	0	0
	Possible implication in pea protein degradation	M49	MER0028047	LIPO	0	0	1	0	0	0
	Possible implication in pea protein degradation	S08A	MER0001521	LIPO	0	0	1	0	0	0
		S16	MER0014135	OTHER	1	0	0	0	0	0
		U32	MER0013876	OTHER	1	0	0	0	0	0
		C10	MER0001723	OTHER	1	0	0	0	0	0

	M24B	MER0001244	OTHER	1	0	0	0	0	0
	S66	MER0016191	OTHER	1	0	0	0	0	0
	M38	MER0033186	OTHER	1	0	0	0	0	0
	M24A	MER0005690	OTHER	1	0	0	0	0	0
	M24B	MER0003105	OTHER	1	0	0	0	0	0
	S41A	MER0003514	OTHER	0.5897	0.13572	0.27427	0.00015	0.0001	8.7E-05
	U32	MER0003242	OTHER	1	0	0	0	0	0
Possible implication in pea protein degradation	M16B	MER0003448	OTHER	1	0	0	0	0	0
	M20A	MER0028025	OTHER	1	0	0	0	0	0
	M38	MER0066192	OTHER	1	0	0	0	0	0
	M103	MER0028087	OTHER	1	0	0	0	0	0
	S14	MER0000474	OTHER	1	0	0	0	0	0
	S41A	MER0001297	OTHER	0.99812	0.00187	1E-06	1E-06	1E-06	1E-06
	C26	MER0045886	OTHER	1	0	0	0	0	0
	M20B	MER0001421	OTHER	1	0	0	0	0	0
	M38	MER0005767	OTHER	1	0	0	0	0	0
	C26	MER0043394	OTHER	1	0	0	0	0	0
	C44	MER0034539	OTHER	1	0	0	0	0	0
	C44	MER0198917	OTHER	1	0	0	0	0	0
	C44	MER0003327	OTHER	1	0	0	0	0	0
	C26	MER0060647	OTHER	1	0	0	0	0	0
	C26	MER0437468	OTHER	1	0	0	0	0	0
	M24A	MER0001243	OTHER	1	0	0	0	0	0
	M38	MER0066192	OTHER	1	0	0	0	0	0
	M38	MER0066192	OTHER	1	0	0	0	0	0
	C11X	MER0030326	OTHER	1	0	0	0	0	0
	S16	MER0000485	OTHER	1	0	0	0	0	0
	S01C	MER0013623	OTHER	0.97113	0.02881	2.3E-05	3.4E-05	2.8E-05	0.00002
	C26	MER0065588	OTHER	0.99999	0	0	0	0	0
	M20C	MER0001283	OTHER	1	0	0	0	0	0
	C56	MER0042827	OTHER	1	0	0	0	0	0

	S49A	MER0001299	OTHER	0.99999	1E-06	0	0	0	0
	M20F	MER0026494	OTHER	0.99999	0	0	0	0	0
	M20C	MER0047723	OTHER	1	0	0	0	0	0
	M41	MER0056525	OTHER	1	3E-06	0	0	0	0
	S37	MER0001350	SP	0.00021	0.9992	0.00017	0.00017	0.00013	0.00013
	S12	MER0004090	SP	0.00017	0.99927	0.00016	0.00016	0.00013	0.00013
	S41A	MER0003413	SP	0.00018	0.99926	0.00016	0.00015	0.00013	0.00013
	S13	MER0000471	SP	0.0002	0.99917	0.00019	0.00017	0.00014	0.00014
Possible implication in pea protein degradation	S09B	MER0005196	SP	0.00019	0.99922	0.00017	0.00017	0.00014	0.00013
	S46	MER0014366	SP	0.00018	0.9992	0.00016	0.00016	0.00014	0.00014
Possible implication in pea protein degradation	S09B	MER0001423	SP	0.00029	0.99897	0.00023	0.00019	0.00017	0.00016
	M03A	MER0001161	SP	0.00023	0.99911	0.00019	0.00017	0.00014	0.00015
Possible implication in pea protein degradation	M16B	MER0001233	SP	0.00012	0.83471	0.16489	0.0001	9.1E-05	9.1E-05
	M48C	MER0002639	SP	0.00013	0.83278	0.16679	0.00011	9.2E-05	9.7E-05
	S46	MER0039993	SP	0.00018	0.99922	0.00016	0.00017	0.00014	0.00014
	S46	MER0039993	SP	0.00016	0.99928	0.00014	0.00015	0.00013	0.00013
Possible implication in pea protein degradation	S09B	MER0004921	SP	0.00018	0.99923	0.00016	0.00017	0.00014	0.00014
Possible implication in pea protein degradation	M16B	MER0001233	SP	0.00043	0.99847	0.00034	0.0003	0.00024	0.00024
Possible implication in pea protein degradation	C01B	MER0002481	SP	0.0002	0.99917	0.00017	0.00017	0.00014	0.00014
Possible implication in pea protein degradation	C01A	MER0095620	SP	0.0002	0.9992	0.00017	0.00017	0.00014	0.00013
	M103	MER0016301	TAT	0	0	0	1	0	0

Supplementary Table 4. DNA sequencing and data filtering

			Sample	Sequenced reads	High quality reads	Classified reads
**S1**	**A**	**CTRL**	**S1-A-CTRL**	361766	317695	99662
**S1**	**B**	**CTRL**	**S1-B-CTRL**	297494	271760	99750
**S1**	**C**	**CTRL**	**S1-C-CTRL**	304357	283260	99600
**S1**	**A**	**PPC**	**S1-A-PPC**	494775	391355	99201
**S1**	**B**	**PPC**	**S1-B-PPC**	444137	405729	99064
**S1**	**C**	**PPC**	**S1-C-PPC**	328865	259958	99226
**S1**	**A**	**PPI**	**S1-A-PPI**	355028	311280	99324
**S1**	**B**	**PPI**	**S1-B-PPI**	228780	209293	99489
**S1**	**C**	**PPI**	**S1-C-PPI**	428618	394572	99148
**S2**	**A**	**CTRL**	**S2-A-CTRL**	450176	387987	98852
**S2**	**B**	**CTRL**	**S2-B-CTRL**	501145	451384	99073
**S2**	**C**	**CTRL**	**S2-C-CTRL**	269541	196232	98942
**S2**	**A**	**PPC**	**S2-A-PPC**	293278	262155	97942
**S2**	**B**	**PPC**	**S2-B-PPC**	434704	384592	98149
**S2**	**C**	**PPC**	**S2-C-PPC**	453835	417520	98658
**S2**	**A**	**PPI**	**S2-A-PPI**	342331	255259	98513
**S2**	**B**	**PPI**	**S2-B-PPI**	452008	406846	98559
**S2**	**C**	**PPI**	**S2-C-PPI**	287222	246788	98396
**S3**	**A**	**CTRL**	**S3-A-CTRL**	713160	604106	99412
**S3**	**B**	**CTRL**	**S3-B-CTRL**	410774	389648	99433
**S3**	**C**	**CTRL**	**S3-C-CTRL**	452295	398300	99452
**S3**	**A**	**PPC**	**S3-A-PPC**	528150	483084	98187
**S3**	**B**	**PPC**	**S3-B-PPC**	410894	378547	98224
**S3**	**C**	**PPC**	**S3-C-PPC**	451724	422042	98448
**S3**	**A**	**PPI**	**S3-A-PPI**	543327	492291	99226
**S3**	**B**	**PPI**	**S3-B-PPI**	617406	508260	98796
**S3**	**C**	**PPI**	**S3-C-PPI**	554729	488706	99239
**S4**	**A**	**CTRL**	**S4-A-CTRL**	474808	413053	99298
**S4**	**B**	**CTRL**	**S4-B-CTRL**	365196	333702	99192
**S4**	**C**	**CTRL**	**S4-C-CTRL**	389248	373662	99230
**S4**	**A**	**PPC**	**S4-A-PPC**	446025	401968	99330
**S4**	**B**	**PPC**	**S4-B-PPC**	245309	221044	99333
**S4**	**C**	**PPC**	**S4-C-PPC**	414804	366494	99235
**S4**	**A**	**PPI**	**S4-A-PPI**	370185	333832	98585
**S4**	**B**	**PPI**	**S4-B-PPI**	457922	434812	98754
**S4**	**C**	**PPI**	**S4-C-PPI**	484211	421563	98859
**S5**	**A**	**CTRL**	**S5-A-CTRL**	234907	226193	99477
**S5**	**B**	**CTRL**	**S5-B-CTRL**	364624	322673	99381
**S5**	**C**	**CTRL**	**S5-C-CTRL**	399156	376475	99683
**S5**	**A**	**PPC**	**S5-A-PPC**	284831	258191	99716
**S5**	**B**	**PPC**	**S5-B-PPC**	399779	359961	99711
**S5**	**C**	**PPC**	**S5-C-PPC**	136970	130451	99673
**S5**	**A**	**PPI**	**S5-A-PPI**	350081	298837	99580
**S5**	**B**	**PPI**	**S5-B-PPI**	323653	308260	99498
**S5**	**C**	**PPI**	**S5-C-PPI**	360514	323847	99565
**S6**	**A**	**CTRL**	**S6-A-CTRL**	167512	162041	98711
**S6**	**B**	**CTRL**	**S6-B-CTRL**	249891	244491	99137
**S6**	**C**	**CTRL**	**S6-C-CTRL**	164623	159675	99114
**S6**	**A**	**PPC**	**S6-A-PPC**	80274	78528	48160
**S6**	**B**	**PPC**	**S6-B-PPC**	75534	73484	50171
**S6**	**C**	**PPC**	**S6-C-PPC**	171184	168344	96388
**S6**	**A**	**PPI**	**S6-A-PPI**	48860	46766	40044
**S6**	**B**	**PPI**	**S6-B-PPI**	189074	183755	98441
**S6**	**C**	**PPI**	**S6-C-PPI**	102168	99624	68018

Supplementary Table 5. Relative abundance of bacterial species identified across all samples

Bacterial species	Abditibacterium below species threshold	Acetatifactor below species threshold	Acetivibrio below species threshold	Acetobacter below species threshold	Acidaminococcus below species threshold	Acidaminococcus fermentans	Acidaminococcus massiliensis	Acidaminococcus timonensis	Acutalibacter below species threshold	Adlercreutzia below species threshold	Aeromonas below species threshold	Aeromonas caviae	Aeromonas hydrophila	Aeromonas veronii	Agathobacter below species threshold
S1-A-CTRL	0	0	0	0	0	0	0	0	0	0	0	0	0	0	0
S1-B-CTRL	0	0	0	0	0	0	0	0	0	0	0	0	0	0	0
S1-C-CTRL	0	0	0	0	0	0	0	0	0	0	0	0	0	0	0
S1-A-PPC	0	0	0	0	0	0	0	0	0	0	0	0	0	0	0
S1-B-PPC	0	0	0	0	0	0	0	0	0	0	0	0	0	0	0
S1-C-PPC	0	0	0	0	0	0	0	0	0	0	0	0	0	0	0
S1-A-PPI	0	0	0	0	0	0	0	0	0	0	0	0	0	0	0
S1-B-PPI	0	0	0	0	0	0	0	0	0	0	0	0	0	0	0
S1-C-PPI	0	0	0	0	0	0	0	0	0	0	0	0	0	0	0
S2-A-CTRL	0	0	0	0	0	0	0	0	0	0	0	0	0.01	0	0.03
S2-B-CTRL	0	0	0	0	0	0	0	0	0	0	0	0	0	0	0.02
S2-C-CTRL	0	0	0	0	0	0	0	0	0	0	0	0	0	0	0.02
S2-A-PPC	0	0	0	0	0	0	0	0	0	0	0	0	0	0	0.07
S2-B-PPC	0	0	0	0	0	0	0	0	0	0	0	0	0	0	0.06
S2-C-PPC	0	0	0	0	0	0	0	0	0	0	0	0	0	0	0.05
S2-A-PPI	0	0	0	0	0	0	0	0	0	0	0	0	0	0	0.04
S2-B-PPI	0	0	0	0	0	0	0	0	0	0	0	0	0	0	0.05
S2-C-PPI	0	0	0	0	0	0	0	0	0	0	0.01	0	0.01	0	0.03
S3-A-CTRL	0	0	0	0	0	0	0	0	0	0	0	0	0	0.12	0
S3-B-CTRL	0	0	0	0	0	0	0	0	0	0	0	0	0	0	0
S3-C-CTRL	0	0	0	0	0	0	0	0	0	0	0	0	0	0	0
S3-A-PPC	0	0	0	0	0.02	0	0	0	0	0	0	0	0	0	0
S3-B-PPC	0	0	0	0	0	0	0	0	0	0	0	0	0	0	0
S3-C-PPC	0	0	0	0	0	0	0	0	0	0	0	0	0	0	0
S3-A-PPI	0	0	0	0	0	0	0	0	0	0	0	0	0	0	0
S3-B-PPI	0	0	0	0	0	0	0	0	0	0	0	0	0	0	0
S3-C-PPI	0	0	0	0	0	0	0	0	0	0	0	0	0	0	0
S4-A-CTRL	0	0	0	0	0	0	0	0	0	0	0	0	0	0	0
S4-B-CTRL	0	0	0	0	0	0	0	0	0	0	0	0	0	0	0
S4-C-CTRL	0	0	0	0	0	0	0	0	0	0	0	0	0	0	0
S4-A-PPC	0	0	0	0	0	0	0	0	0	0	0	0	0	0	0
S4-B-PPC	0	0	0	0	0	0	0	0	0	0	0	0	0	0	0
S4-C-PPC	0	0	0	0	0	0	0	0	0	0	0	0	0	0	0
S4-A-PPI	0	0	0	0	0	0	0	0	0	0	0	0	0	0	0
S4-B-PPI	0	0	0	0	0	0	0	0	0	0	0	0	0	0	0
S4-C-PPI	0	0	0	0	0	0	0	0	0	0	0	0	0	0	0
S5-A-CTRL	0	0	0	0	0	0	0	0	0	0	0	0	0	0	0
S5-B-CTRL	0.83	0	0	0	0	0	0	0	0	0	0	0	0	0	0
S5-C-CTRL	0	0	0	0	0	0	0	0	0	0	0	0	0	0	0
S5-A-PPC	0	0	0	0	0	0	0	0	0	0	0	0	0	0	0
S5-B-PPC	0	0	0	0	0	0	0	0	0	0	0	0	0	0	0
S5-C-PPC	0	0	0	0	0	0	0	0	0	0	0	0	0	0	0
S5-A-PPI	0	0	0	0	0	0	0	0	0	0	0	0	0	0	0
S5-B-PPI	0	0	0	0	0	0	0	0	0	0	0	0	0	0	0
S5-C-PPI	0	0	0	0	0	0	0	0	0	0	0	0	0	0	0
S6-A-CTRL	0	0	0	0	0	0	0	0	0	0	0	0.23	0	0	0
S6-B-CTRL	0	0	0	0	0	0	0	0	0	0	0	0.26	0	0	0
S6-C-CTRL	0	0	0	0	0	0	0	0	0	0	0	0.34	0	0	0
S6-A-PPC	0	0	0	0	0	0	0	0	0	0	0	0	0	0	0
S6-B-PPC	0	0	0	0	0	0	0	0	0	0	0	0	0	0	0
S6-C-PPC	0	0	0	0	0	0	0	0	0	0	0	0	0	0	0
S6-A-PPI	0	0	0	0	0	0	0	0	0	0	0	0.05	0	0	0
S6-B-PPI	0	0	0	0	0.08	0	0	0	0	0	0	0.12	0	0	0
S6-C-PPI	0	0	0	0	0.07	0	0	0	0	0	0	0.06	0	0	0

Bacterial species	Agathobacter rectalis	Agathobaculum below species threshold	Agathobaculum butyriciproducens	Agathobaculum multihit species	Akkermansia below species threshold	Akkermansia massiliensis	Akkermansia muciniphila	Akkermansia multihit species	Alistipes below species threshold	Alistipes communis	Alistipes dispar	Alistipes finegoldii	Alistipes ihumii	Alistipes indistinctus	Alistipes intestinihominis	Alistipes montrealensis	Alistipes multihit species	Alistipes onderdonkii
**S1-A-CTRL**	0.4	0	0	0	0	0	0	0	0.68	0	0	0	0	0	0	0	0.35	1.25
**S1-B-CTRL**	0.43	0	0	0	0	0	0	0	0.49	0	0	0	0	0	0	0	0	0.58
**S1-C-CTRL**	0.5	0	0	0	0	0	0	0	0.59	0	0	0	0	0	0	0	0.26	0.96
**S1-A-PPC**	0.47	0	0	0	0	0	0	0	0.12	0	0.17	0	0	0	0	0	0.27	0.57
**S1-B-PPC**	0.44	0	0	0	0	0	0	0	0	0	0	0	0	0	0	0	0.12	0.4
**S1-C-PPC**	0.54	0	0	0	0	0	0	0	0.13	0	0	0	0	0	0	0	0	0.27
**S1-A-PPI**	0.37	0	0	0	0	0	0	0	0.33	0	0	0	0	0	0	0	0.17	0.82
**S1-B-PPI**	0.69	0	0	0	0	0	0	0	0.46	0	0	0	0	0	0	0	0	0.86
**S1-C-PPI**	0.41	0	0	0	0	0	0	0	0.33	0	0	0	0	0	0	0	0.2	1.03
**S2-A-CTRL**	2.4	0	0.09	0	0	0.05	0	0	0.07	0	0	0	0	0	0	0	0.17	1.22
**S2-B-CTRL**	2.25	0	0.04	0	0	0.04	0	0	0.05	0	0	0	0	0	0	0	0.13	1.01
**S2-C-CTRL**	2.39	0	0.06	0	0	0.03	0	0	0.05	0	0	0	0	0	0	0	0.18	1.25
**S2-A-PPC**	5.03	0.02	0.09	0	0	0.02	0	0	0.06	0	0	0	0	0	0	0	0.12	1.09
**S2-B-PPC**	4.13	0.04	0.08	0	0	0.02	0	0	0.05	0	0	0	0	0	0	0	0.12	1.05
**S2-C-PPC**	3.53	0	0.05	0	0	0.03	0	0	0.03	0	0	0	0	0	0	0	0.1	0.84
**S2-A-PPI**	3.17	0.02	0.08	0	0	0.04	0	0	0.06	0	0	0	0	0	0	0	0.16	1.16
**S2-B-PPI**	3.88	0	0.08	0	0	0.02	0	0	0.05	0	0	0	0	0	0	0	0.17	1.37
**S2-C-PPI**	3.45	0.02	0.07	0	0	0.02	0	0	0.05	0	0	0	0	0	0	0	0.13	1.08
**S3-A-CTRL**	0	0	0	0	0	0	0	0	0	0	0	0	0	0	0	0	0	0
**S3-B-CTRL**	0	0	0	0	0	0	0	0	0	0	0	0	0	0	0	0	0	0
**S3-C-CTRL**	0	0	0	0	0	0	0	0	0	0.28	0	0	0	0	0	0	0	0
**S3-A-PPC**	0.04	0	0	0	0	0	0	0	0.03	0.03	0.03	0	0	0	0	0	0.02	0.02
**S3-B-PPC**	0.03	0	0	0	0	0	0	0	0.04	0	0.05	0	0	0	0	0	0.03	0
**S3-C-PPC**	0.04	0	0	0	0	0	0	0	0	0.02	0.04	0	0	0	0	0	0	0
**S3-A-PPI**	0.15	0	0	0	0	0	0	0	0	0	0.13	0	0	0	0	0	0	0
**S3-B-PPI**	0.08	0	0	0	0	0	0	0	0	0	0	0	0	0	0	0	0	0
**S3-C-PPI**	0	0	0	0	0	0	0	0	0	0	0	0	0	0	0	0	0	0
**S4-A-CTRL**	0	0	0	0	0	0	0	0	0.16	0	0	0.24	0	0	0	0	0.1	0.53
**S4-B-CTRL**	0	0	0	0	0	0	0	0	0.1	0	0	0.39	0.07	0	0	0	0.12	0.54
**S4-C-CTRL**	0	0	0	0	0	0	0	0	0.24	0	0	0.54	0	0	0	0	0.13	0.55
**S4-A-PPC**	0	0	0	0	0	0	0	0	0.56	0	0	2.73	0	0	0	0	0.59	1.4
**S4-B-PPC**	0	0	0	0	0	0	0	0	0.58	0	0	2.28	0	0	0	0	0.71	0.81
**S4-C-PPC**	0	0	0	0	0	0	0	0	0.5	0	0	3.14	0.51	0	0	0	0.7	0.92
**S4-A-PPI**	0.06	0	0	0	0	0	0	0	0.19	0	0	0.75	0.08	0	0	0	0.21	0.45
**S4-B-PPI**	0	0	0	0	0	0	0	0	0.22	0	0	0.78	0	0	0	0	0.22	0.6
**S4-C-PPI**	0	0	0	0	0	0	0	0	0.3	0	0	1.22	0	0	0	0	0.32	0.53
**S5-A-CTRL**	0	0	0	0	0	0	0.87	0	0	0	0	0	0	0	0	0	0	0.81
**S5-B-CTRL**	0	0	0	0	0	0	0.69	0	0	0	0	0.32	0	0	0	0	0	1.05
**S5-C-CTRL**	0	0	0	0	0	0	1	0	0	0	0	0	0	0	0	0	0	1.06
**S5-A-PPC**	0	0	0	0	0	0	1.18	0	0	0	0	0	0	0	0	0	0	0.86
**S5-B-PPC**	0	0	0	0	0	0	1.39	0	0	0	0	0	0	0	0	0	0	0.9
**S5-C-PPC**	0	0	0	0	0	0	1.25	0	0	0	0	0	0	0	0	0	0	1.12
**S5-A-PPI**	0	0	0	0	0	0	1	0	0	0	0	0.31	0	0	0	0	0.34	1.96
**S5-B-PPI**	0	0	0	0	0	0	0.93	0	0	0	0	0	0	0	0	0	0	1.11
**S5-C-PPI**	0	0	0	0	0	0	0.71	0	0	0	0	0.18	0	0	0	0	0.21	1
**S6-A-CTRL**	0.82	0	0	0	0	0	0	0	0	0	0	0	0	0	0	0	0	0
**S6-B-CTRL**	0.58	0	0	0	0	0	0	0	0	0	0	0	0	0	0	0	0	0
**S6-C-CTRL**	0.73	0	0	0	0	0	0	0	0	0	0	0	0	0	0	0	0	0
**S6-A-PPC**	0.77	0	0	0	0	0	0	0	0	0	0	0	0	0	0	0	0	0
**S6-B-PPC**	0.7	0	0	0	0	0	0	0	0	0	0	0	0	0	0	0	0	0
**S6-C-PPC**	0.76	0	0	0	0	0	0	0	0	0	0	0	0	0	0	0	0	0
**S6-A-PPI**	1.46	0	0	0	0	0	0	0	0	0	0	0	0	0	0	0	0	0
**S6-B-PPI**	1.36	0	0	0	0	0	0	0	0	0	0	0	0	0	0	0	0	0
**S6-C-PPI**	1.56	0	0	0	0	0	0	0	0	0	0	0	0	0	0	0	0	0

**Bacterial species**	Alistipes provencensis	Alistipes putredinis	Alistipes senegalensis	Alistipes shahii	Alistipes timonensis	Allisonella below species threshold	Allisonella histaminiformans	Allocoprobacillus below species threshold	Allofournierella below species threshold	Allofournierella massiliensis	Alloprevotella below species threshold	Amedibacterium below species threshold	Anaerobutyricum below species threshold	Anaerobutyricum hallii	Anaerocaecibacter below species threshold	Anaerocolumna below species threshold
**S1-A-CTRL**	0	0	0	0	0	0	1.71	0	0	0	0	0	0	0	0	0
**S1-B-CTRL**	0	0	0	0	0	0	1.36	0	0	0	0	0	0	0	0	0
**S1-C-CTRL**	0	0	0	0	0	0	1.22	0	0	0	0	0	0	0	0	0
**S1-A-PPC**	0	0	0	0	0	0	0	0	0	0	0	0	0	0	0	0
**S1-B-PPC**	0	0	0	0	0	0	0	0	0	0	0	0	0	0	0	0
**S1-C-PPC**	0	0	0	0	0	0	0	0	0	0	0	0	0	0	0	0
**S1-A-PPI**	0	0	0	0	0	0	0.54	0	0	0	0	0	0	0	0	0
**S1-B-PPI**	0	0	0	0	0	0	0.41	0	0	0	0	0	0	0	0	0
**S1-C-PPI**	0	0	0	0	0	0	1.02	0	0	0	0	0	0	0	0	0
**S2-A-CTRL**	0	0	0	0.03	0	0	0	0	0	0	0	0	0	0	0	0
**S2-B-CTRL**	0	0	0	0.03	0	0	0	0	0	0	0	0	0	0	0	0
**S2-C-CTRL**	0	0	0	0.02	0	0	0	0	0	0	0	0	0	0	0	0
**S2-A-PPC**	0	0	0	0.06	0	0	0	0	0	0	0	0	0	0	0	0
**S2-B-PPC**	0	0	0	0.05	0	0	0	0	0	0	0	0	0	0	0	0
**S2-C-PPC**	0	0	0	0.02	0	0	0	0	0	0	0	0	0	0	0	0
**S2-A-PPI**	0	0	0	0.05	0	0	0	0	0	0	0	0	0	0	0	0
**S2-B-PPI**	0	0	0	0.04	0	0	0	0	0	0	0	0	0	0	0	0
**S2-C-PPI**	0	0	0	0.02	0	0	0	0	0	0	0	0	0	0	0	0
**S3-A-CTRL**	0	0.64	0	0	0	0	0.9	0	0	0	0	0	0	0	0	0
**S3-B-CTRL**	0	1	0	0	0	0.56	0.91	0	0	0	0	0	0	0	0	0
**S3-C-CTRL**	0	1	0	0	0	0	1.35	0	0	0	0	0	0	0	0	0
**S3-A-PPC**	0	0.12	0	0.02	0	0	0	0	0	0	0	0	0	0.03	0	0
**S3-B-PPC**	0	0.14	0	0	0	0	0	0	0	0	0	0	0	0.02	0	0
**S3-C-PPC**	0	0.12	0	0	0	0	0.05	0	0	0	0	0	0	0	0	0
**S3-A-PPI**	0	0.4	0	0	0	0	0.41	0	0	0	0	0	0	0	0	0
**S3-B-PPI**	0	0.13	0	0	0	0	0	0	0	0	0	0	0	0	0	0
**S3-C-PPI**	0	0.25	0	0	0	0	0	0	0	0	0	0	0	0	0	0
**S4-A-CTRL**	0	0.71	0	0.11	0	0	0	0	0	0	0	0	0	0	0	0
**S4-B-CTRL**	0	0.75	0	0.17	0	0	0	0	0	0	0	0	0	0	0	0
**S4-C-CTRL**	0	0.8	0	0.24	0.05	0	0	0	0	0	0	0	0	0	0	0
**S4-A-PPC**	0	3.09	0	0.51	0	0	0	0	0	0	0	0	0	0	0	0
**S4-B-PPC**	0	1.71	0	0.65	0	0	0	0	0	0	0	0	0	0	0	0
**S4-C-PPC**	0	1.81	0	1.31	0	0	0	0	0	0	0	0	0	0	0	0
**S4-A-PPI**	0	1.15	0	0.15	0.05	0	0	0	0	0	0	0	0	0	0	0
**S4-B-PPI**	0	0.97	0	0.26	0.07	0	0	0	0	0	0	0	0	0	0	0
**S4-C-PPI**	0	1.27	0	0.27	0.11	0	0	0	0	0	0	0	0	0	0	0
**S5-A-CTRL**	0	0.67	0	0	0	0	0	0	0	0	0	0	0	0	0	0
**S5-B-CTRL**	0	1.17	0	0	0	0	0	0	0	0	0	0	0	0	0	0
**S5-C-CTRL**	0	0.76	0	0	0	0	0	0	0	0	0	0	0	0	0	0
**S5-A-PPC**	0	0.96	0	0	0	0	0	0	0	0	0	0	0	0	0	0
**S5-B-PPC**	0	0.89	0	0	0	0	0	0	0	0	0	0	0	0	0	0
**S5-C-PPC**	0	0.78	0	0	0	0	0	0	0	0	0	0	0	0	0	0
**S5-A-PPI**	0	0.79	0	0	0	0	0	0	0	0	0	0	0	0	0	0
**S5-B-PPI**	0	0.64	0	0	0	0	0	0	0	0	0	0	0	0	0	0
**S5-C-PPI**	0	0.54	0	0	0	0	0	0	0	0	0	0	0	0	0	0
**S6-A-CTRL**	0	1.64	0	0	0	0	0	0	0	0	0	0	0	0	0	0
**S6-B-CTRL**	0	1.45	0	0	0	0	0	0	0	0	0	0	0	0	0	0
**S6-C-CTRL**	0	1.39	0	0	0	0	0	0	0	0	0	0	0	0	0	0
**S6-A-PPC**	0	0.14	0	0	0	0	0	0	0	0	0	0	0	0	0	0
**S6-B-PPC**	0	0.09	0	0	0	0	0	0	0	0	0	0	0	0	0	0
**S6-C-PPC**	0	0.15	0	0	0	0.08	0	0	0	0	0	0	0	0	0	0
**S6-A-PPI**	0	0.24	0	0	0	0	0	0	0	0	0	0	0	0	0	0
**S6-B-PPI**	0	0.29	0	0	0	0	0	0	0	0	0	0	0	0	0	0
**S6-C-PPI**	0	0.27	0	0	0	0.15	0	0	0	0	0	0	0	0	0	0

**Bacterial species**	Anaeromassilibacillus below species threshold	Anaeromicropila below species threshold	Anaeroplasma below species threshold	Anaerosporobacter below species threshold	Anaerostipes amylophilus	Anaerostipes below species threshold	Anaerostipes hadrus	Anaerotignum below species threshold	Anaerotignum faecicola	Anaerotignum lactatifermentans	Anaerotruncus below species threshold	Anaerotruncus colihominis	Anaerotruncus rubiinfantis	Anthropogastromicrobium aceti	Anthropogastromicrobium below species threshold	Aristaeella below species threshold
**S1-A-CTRL**	0	0	0	0	0	0	0	0	0	0	0	0	0	0	0	0
**S1-B-CTRL**	0	0	0	0	0	0	0	0	0	0	0	0	0	0	0	0
**S1-C-CTRL**	0	0	0	0	0	0	0.22	0	0	0	0	0	0	0	0	0
**S1-A-PPC**	0	0	0	0	0	0	1.09	0	0	0	0	0	0	0	0	0
**S1-B-PPC**	0	0	0	0	0	0	1.57	0	0	0	0	0	0	0	0	0
**S1-C-PPC**	0	0	0	0	0	0	1.13	0	0	0	0	0	0	0	0	0
**S1-A-PPI**	0	0	0	0	0	0	0.27	0	0	0	0	0	0	0	0	0
**S1-B-PPI**	0	0	0	0	0	0	0.4	0	0	0	0	0	0	0	0	0
**S1-C-PPI**	0	0	0	0	0	0	0.23	0	0	0	0	0	0	0	0	0
**S2-A-CTRL**	0	0	0	0	0	0	0.04	0	0	0	0	0	0	0	0	0
**S2-B-CTRL**	0	0	0	0	0	0	0.02	0	0	0	0	0	0	0	0	0
**S2-C-CTRL**	0	0	0	0	0	0.02	0.02	0	0	0	0	0	0	0	0	0
**S2-A-PPC**	0	0	0	0	0	0.03	0.1	0	0	0	0	0	0	0	0	0
**S2-B-PPC**	0	0	0	0	0	0.02	0.08	0	0	0	0	0	0	0	0	0
**S2-C-PPC**	0	0	0	0	0	0	0.06	0	0	0	0	0	0	0	0	0
**S2-A-PPI**	0	0	0	0	0	0.02	0.04	0	0	0	0	0	0	0	0	0.02
**S2-B-PPI**	0	0	0	0	0	0.03	0.08	0	0	0	0	0	0	0	0	0
**S2-C-PPI**	0	0	0	0	0	0.02	0.06	0	0	0	0	0	0	0	0	0
**S3-A-CTRL**	0	0	0	0	0	0	0	0	0	0	0	0	0	0	0	0
**S3-B-CTRL**	0	0	0	0	0	0	0	0	0	0	0	0	0	0	0	0
**S3-C-CTRL**	0	0	0	0	0	0	0	0	0	0	0	0	0	0	0	0
**S3-A-PPC**	0	0	0	0	0	0	0	0	0	0	0	0	0	0	0	0
**S3-B-PPC**	0	0	0	0	0	0	0	0	0	0	0	0	0	0	0	0
**S3-C-PPC**	0	0	0	0	0	0	0.02	0	0	0	0	0	0	0	0	0
**S3-A-PPI**	0	0	0	0	0	0	0	0	0	0	0	0	0	0	0	0
**S3-B-PPI**	0	0	0	0	0	0	0	0	0	0	0	0	0	0	0	0
**S3-C-PPI**	0	0	0	0	0	0	0	0	0	0	0	0	0	0	0	0
**S4-A-CTRL**	0	0	0	0	0	0	0	0	0	0	0	0	0	0	0	0
**S4-B-CTRL**	0	0	0	0	0	0	0	0	0	0	0	0	0	0	0	0
**S4-C-CTRL**	0	0	0	0	0	0	0	0	0	0	0	0	0	0	0	0
**S4-A-PPC**	0	0	0	0	0	0	0	0	0	0	0	0	0	0	0	0
**S4-B-PPC**	0	0	0	0	0	0	0	0	0	0	0	0	0	0	0	0
**S4-C-PPC**	0	0	0	0	0	0	0	0	0	0	0	0	0	0	0	0
**S4-A-PPI**	0	0	0	0	0	0	0	0	0	0	0	0	0	0	0	0
**S4-B-PPI**	0	0	0	0	0	0	0	0	0	0	0	0	0	0	0	0
**S4-C-PPI**	0	0	0	0	0	0	0	0	0	0	0	0	0	0	0	0
**S5-A-CTRL**	0	0	0	0	0	0	0	0	0	0	0	0	0	0	0	0
**S5-B-CTRL**	0	0	0	0	0	0	0	0	0	0	0	0	0	0	0	0
**S5-C-CTRL**	0	0	0	0	0	0	0	0	0	0	0	0	0	0	0	0
**S5-A-PPC**	0	0	0	0	0	0	0	0	0	0	0	0	0	0	0	0
**S5-B-PPC**	0	0	0	0	0	0	0	0	0	0	0	0	0	0	0	0
**S5-C-PPC**	0	0	0	0	0	0	0.34	0	0	0	0	0	0	0	0	0
**S5-A-PPI**	0	0	0	0	0	0	0	0	0	0	0	0	0	0	0	0
**S5-B-PPI**	0	0	0	0	0	0	0	0	0	0	0	0	0	0	0	0
**S5-C-PPI**	0	0	0	0	0	0	0	0	0	0	0	0	0	0	0	0
**S6-A-CTRL**	0	0	0	0	0	0	0	0	0	0	0	0	0	0	0	0
**S6-B-CTRL**	0	0	0	0	0	0	0	0	0	0	0	0	0	0	0	0
**S6-C-CTRL**	0	0	0	0	0	0	0	0	0	0	0	0	0	0	0	0
**S6-A-PPC**	0	0	0	0	0.05	0	0	0	0	0	0	0	0	0	0	0
**S6-B-PPC**	0	0	0	0	0	0	0	0	0	0	0	0	0	0	0	0
**S6-C-PPC**	0	0	0	0	0	0	0	0	0	0	0	0	0	0	0	0
**S6-A-PPI**	0	0	0	0	0	0	0	0	0	0	0	0	0	0	0	0
**S6-B-PPI**	0	0	0	0	0	0	0	0	0	0	0	0	0	0	0	0
**S6-C-PPI**	0	0	0	0	0	0	0	0	0	0	0	0	0	0	0	0

**Bacterial species**	Bacillus below species threshold	Bacillus multihit species	Bacillus paranthracis	Bacteroides acidifaciens	Bacteroides below species threshold	Bacteroides caccae	Bacteroides caecimuris	Bacteroides cellulosilyticus	Bacteroides clarus	Bacteroides congonensis	Bacteroides eggerthii	Bacteroides faecis	Bacteroides faecium	Bacteroides finegoldii	Bacteroides fragilis	Bacteroides hominis
**S1-A-CTRL**	0	0	0	0	0.46	0	0	0.21	0	0	1.6	0	0	0	0	0
**S1-B-CTRL**	0	0	0	0	0.49	0	0	0.22	0	0	1.08	0	0	0	0	0
**S1-C-CTRL**	0	0	0	0	0.7	0.15	0	0.2	0	0	1.32	0	0	0	0	0
**S1-A-PPC**	0	0	0	0	0.28	0	0	0.06	0	0	0.51	0	0	0	0	0
**S1-B-PPC**	0	0	0	0	0.17	0	0	0.07	0	0	0.41	0	0	0	0	0
**S1-C-PPC**	0	0	0	0	0.19	0.09	0	0.06	0	0	0.26	0	0	0	0	0
**S1-A-PPI**	0	0	0	0	0.3	0	0	0	0	0	0.45	0	0	0	0	0
**S1-B-PPI**	0	0	0	0	0.53	0.24	0	0	0	0	0.5	0	0	0	0	0
**S1-C-PPI**	0	0	0	0	0.36	0.15	0	0.05	0	0	0.36	0	0	0	0	0
**S2-A-CTRL**	0	0	0	0	0.05	0.06	0	0	0	0.3	0	0	0.01	0	0.05	0
**S2-B-CTRL**	0	0.02	0.01	0	0.05	0.05	0	0	0	0.34	0	0	0.01	0	0.04	0
**S2-C-CTRL**	0	0	0	0.01	0.06	0.05	0	0	0	0.29	0	0	0.01	0	0.04	0
**S2-A-PPC**	0	0	0	0	0.03	0.1	0	0	0	0.08	0	0	0	0	0.01	0
**S2-B-PPC**	0	0	0	0	0.02	0.09	0	0	0	0.07	0	0	0	0	0	0
**S2-C-PPC**	0	0	0	0	0.01	0.08	0	0	0	0.07	0	0	0	0	0	0
**S2-A-PPI**	0	0	0	0	0.04	0.09	0	0	0	0.2	0	0	0	0	0.03	0
**S2-B-PPI**	0	0	0	0	0.03	0.15	0	0	0	0.22	0	0	0	0	0.02	0
**S2-C-PPI**	0	0	0	0	0.04	0.14	0	0	0	0.25	0	0	0	0	0.03	0
**S3-A-CTRL**	0	0	0	0	0.24	0	0	0	0	0	0.47	0	0	0	0	0
**S3-B-CTRL**	0	0	0	0	0.39	0	0	0	0	0	0.49	0	0	0	0	0
**S3-C-CTRL**	0	0	0	0	0.34	0	0	0	0	0	0.61	0	0	0	0	0
**S3-A-PPC**	0	0	0	0	0.04	0.02	0	0	0	0	0.06	0	0	0	0	0
**S3-B-PPC**	0	0	0	0	0.04	0.02	0	0	0	0	0.06	0	0	0	0	0
**S3-C-PPC**	0	0	0	0	0.03	0	0	0	0	0	0.06	0	0	0	0	0
**S3-A-PPI**	0	0	0	0	0.14	0	0	0	0	0	0.45	0	0	0	0	0
**S3-B-PPI**	0	0	0	0	0.14	0.11	0	0	0	0	0.28	0	0	0	0	0
**S3-C-PPI**	0	0	0	0	0.14	0.07	0	0	0	0	0.29	0	0	0	0	0
**S4-A-CTRL**	0	0	0	0	1.47	5.53	0.05	0.43	0.06	0	0	0	0.05	0.1	1.86	0.04
**S4-B-CTRL**	0	0	0	0	1.54	5.08	0	0.55	0.05	0	0	0	0.04	0.07	2.24	0.07
**S4-C-CTRL**	0	0	0	0	1.32	4.99	0.04	0.42	0.05	0	0	0	0	0.07	1.88	0.05
**S4-A-PPC**	0	0	0	0	0.66	1.22	0	0	0	0	0	0	0	0	1.29	0
**S4-B-PPC**	0	0	0	0	0.51	1.27	0	0	0	0	0	0	0	0	1.01	0
**S4-C-PPC**	0	0	0	0	0.77	1.15	0	0.16	0	0	0	0	0	0	0.44	0
**S4-A-PPI**	0	0	0	0	0.58	2.14	0	0.17	0.05	0	0	0	0	0.05	1.44	0
**S4-B-PPI**	0	0	0	0	0.67	2.93	0	0.16	0.04	0	0	0	0	0.03	1.74	0
**S4-C-PPI**	0	0	0	0	0.57	2.36	0	0.12	0.04	0	0	0	0	0	1.32	0
**S5-A-CTRL**	0	0	0	0	4.34	0	0	0.2	0	0	0	0	0	0.79	0.54	0
**S5-B-CTRL**	0	0	0	0	2.87	0	0	0	0	0	0	0	0	0.54	0.28	0
**S5-C-CTRL**	0	0	0	0	2.57	0	0	0	0	0	0	0	0	0.94	0.47	0.31
**S5-A-PPC**	0	0	0	0	0.17	0	0	0	0	0	0	0	0	0.17	0	0
**S5-B-PPC**	0	0	0	0	0.34	0	0	0	0	0	0	0	0	0	0	0
**S5-C-PPC**	0	0	0	0	0.15	0	0	0	0	0	0	0	0	0.21	0	0
**S5-A-PPI**	0	0	0	0	0.84	0	0	0	0	0	0	0	0	0.43	0.6	0
**S5-B-PPI**	0	0	0	0	1.05	0	0	0	0	0	0	0	0	0.37	0.44	0
**S5-C-PPI**	0	0	0	0	0.76	0	0	0	0	0	0	0	0	0.42	0.46	0
**S6-A-CTRL**	0	0	0	0	0	0	0	0	0	0	0	0	0	0	0	0
**S6-B-CTRL**	0	0	0	0	0	0	0	0	0	0	0	0	0	0	0	0
**S6-C-CTRL**	0	0	0	0	0	0	0	0	0	0	0	0	0	0	0	0
**S6-A-PPC**	0	0	0	0	0.01	0	0	0	0	0	0	0	0	0	0	0
**S6-B-PPC**	0	0	0	0	0	0	0	0	0	0	0	0	0	0	0	0
**S6-C-PPC**	0	0	0	0	0.02	0	0	0	0	0	0	0	0	0	0	0
**S6-A-PPI**	0	0	0	0	0	0	0	0	0	0	0	0	0	0	0	0
**S6-B-PPI**	0	0	0	0	0.05	0	0	0	0	0	0	0	0	0	0	0
**S6-C-PPI**	0	0	0	0	0.05	0	0	0	0	0	0	0	0	0	0	0

**Bacterial species**	Bacteroides ilei	Bacteroides intestinalis	Bacteroides mediterraneensis	Bacteroides multihit species	Bacteroides ndongoniae	Bacteroides nordii	Bacteroides oleiciplenus	Bacteroides ovatus	Bacteroides pyogenes	Bacteroides rodentium	Bacteroides salyersiae	Bacteroides stercorirosoris	Bacteroides stercoris	Bacteroides thetaiotaomicron	Bacteroides timonensis	Bacteroides uniformis
**S1-A-CTRL**	0.34	0	0	8.08	0.67	0	0	0.84	0	0	0.27	0	0	6.71	0	12.88
**S1-B-CTRL**	0.23	0	0	7.4	0.57	0	0	1.09	0	0	0.31	0	0	5.19	0	11.69
**S1-C-CTRL**	0.19	0	0	8.34	0.8	0	0	0.9	0	0	0.45	0	0	6.73	0	11.57
**S1-A-PPC**	0	0	0	1.3	0.4	0	0	0.09	0	0	0.21	0	0	0.73	0	2.29
**S1-B-PPC**	0	0	0	1.35	0.48	0	0	0.11	0	0	0.3	0	0	0.71	0	1.72
**S1-C-PPC**	0	0	0	1.29	0.35	0	0	0.14	0	0	0.24	0	0	0.65	0	1.59
**S1-A-PPI**	0	0	0	2.27	0.35	0	0	0.21	0	0	0.18	0	0	1.58	0	3.09
**S1-B-PPI**	0	0	0	2.71	0.56	0	0	0.17	0	0	0.33	0	0	1.94	0	2.97
**S1-C-PPI**	0	0	0	2.77	0.33	0	0	0.36	0	0	0.14	0	0	1.67	0	2.78
**S2-A-CTRL**	0	0	0	0.12	0	0	0	0.01	0	0	0	0	0	0.04	0	0
**S2-B-CTRL**	0	0	0	0.15	0	0	0	0.01	0	0	0	0	0	0.04	0	0
**S2-C-CTRL**	0	0	0	0.15	0	0	0	0	0	0	0	0	0	0.03	0	0
**S2-A-PPC**	0	0	0	0.08	0	0	0	0	0	0	0	0	0	0.04	0	0
**S2-B-PPC**	0	0	0	0.08	0	0	0	0	0	0	0	0	0	0.02	0	0
**S2-C-PPC**	0	0	0	0.06	0	0	0	0	0	0	0	0	0	0.03	0	0
**S2-A-PPI**	0	0	0	0.12	0	0	0	0	0	0	0	0	0	0.04	0	0
**S2-B-PPI**	0	0	0	0.14	0	0	0	0	0	0	0	0	0	0.06	0	0
**S2-C-PPI**	0	0	0	0.11	0	0	0	0.01	0	0	0	0	0	0.05	0	0
**S3-A-CTRL**	0	0	0	4.8	0	0	0	0.2	0	0	0	0	0	0.58	0	2.12
**S3-B-CTRL**	0	0	0	2.34	0	0	0	0	0	0	0	0	0	0.2	0	2.65
**S3-C-CTRL**	0	0	0	2.45	0	0	0	0	0	0	0	0	0	0.16	0	2.22
**S3-A-PPC**	0	0	0	0.12	0	0	0	0	0	0	0	0	0	0	0	0.11
**S3-B-PPC**	0	0	0	0.13	0	0	0	0	0	0	0	0	0	0	0	0.13
**S3-C-PPC**	0	0	0	0.12	0	0	0	0	0	0	0	0	0	0	0	0.12
**S3-A-PPI**	0	0	0	1.29	0	0	0	0	0	0	0	0	0.09	0.1	0	1.36
**S3-B-PPI**	0	0	0	0.37	0	0	0	0	0	0	0	0	0	0.05	0	0.39
**S3-C-PPI**	0	0	0	0.59	0	0	0	0	0	0	0	0	0	0.08	0	0.52
**S4-A-CTRL**	0	0.87	0	24.34	0	0.66	0.02	1.25	0	0	0	0	0.08	2.07	0	0.89
**S4-B-CTRL**	0	1	0	24.45	0	0.64	0.02	1.36	0	0	0	0	0.11	2.28	0.02	0.93
**S4-C-CTRL**	0	1.11	0	22.04	0	0.61	0	1.31	0	0	0	0	0.12	2.33	0	1.05
**S4-A-PPC**	0	0.25	0	3.17	0	0	0	0	0	0	0	0	0	0.61	0	0.74
**S4-B-PPC**	0	0.23	0	3.79	0	0	0	0	0	0	0	0	0	0.62	0	0.35
**S4-C-PPC**	0	0.19	0	3.87	0	0	0	0	0	0	0	0	0	1	0	0.46
**S4-A-PPI**	0	0.29	0	7.18	0	0.27	0	0.36	0	0	0	0	0.11	1.57	0	1.29
**S4-B-PPI**	0	0.26	0	8.6	0	0.21	0	0.43	0	0	0	0	0.09	1.92	0	1.71
**S4-C-PPI**	0	0.2	0	6.9	0	0.14	0	0.28	0	0	0	0	0.08	1.33	0	1.2
**S5-A-CTRL**	0	1.94	0	12.31	0	0	0	5.75	0	0	0.63	0	0	0	0	8.29
**S5-B-CTRL**	0	1.7	0	10.25	0	0	0	5.81	0	0	0.76	0	0	0	0	5.93
**S5-C-CTRL**	0	1.83	0	10.91	0	0	0	5.26	0	0	0.61	0	0	0	0	5.66
**S5-A-PPC**	0	0.47	0	1.48	0	0	0	0.26	0	0	0.29	0	0	0	0	1.18
**S5-B-PPC**	0	0.23	0	1.57	0	0	0	0.38	0	0	0.2	0	0	0	0	1.42
**S5-C-PPC**	0	0.54	0	2.37	0	0	0	0.45	0	0	0.2	0	0	0	0	1.62
**S5-A-PPI**	0	1.04	0	5.16	0	0	0	1.47	0	0	0.45	0	0	0	0	6.47
**S5-B-PPI**	0	0.73	0	4.95	0	0	0	1.34	0	0	0.46	0	0	0	0	5.51
**S5-C-PPI**	0	1.25	0	4.86	0	0	0	1.34	0	0	0.41	0	0	0	0	5.73
**S6-A-CTRL**	0	0	0	0.41	0	0	0	0.13	0	0	0	0	0	0	0	0.39
**S6-B-CTRL**	0	0	0	0.61	0	0	0	0.12	0	0	0	0	0	0	0	0.47
**S6-C-CTRL**	0	0	0	0.44	0	0	0	0.1	0	0	0	0	0	0	0	0.35
**S6-A-PPC**	0	0	0	0.02	0	0	0	0	0	0	0	0	0.03	0	0	0
**S6-B-PPC**	0	0	0	0.04	0	0	0	0	0	0	0	0	0	0	0	0.02
**S6-C-PPC**	0	0	0	0.03	0	0	0	0	0	0	0	0	0.02	0	0	0
**S6-A-PPI**	0	0	0	0.1	0	0	0	0	0	0	0	0	0	0	0	0.1
**S6-B-PPI**	0	0	0	0.15	0	0	0	0	0	0	0	0	0	0	0	0.11
**S6-C-PPI**	0	0	0	0.12	0	0	0	0	0	0	0	0	0	0	0	0.13

**Bacterial species**	Bacteroides vicugnae	Bacteroides xylanisolvens	Bacteroides zhangwenhongii	Barnesiella below species threshold	Barnesiella intestinihominis	Beduinella below species threshold	Bianquea below species threshold	Bifidobacterium adolescentis	Bifidobacterium below species threshold	Bifidobacterium bifidum	Bifidobacterium breve	Bifidobacterium catenulatum	Bifidobacterium dentium	Bifidobacterium longum	Bifidobacterium multihit species	Bifidobacterium pseudocatenulatum
**S1-A-CTRL**	0	0.19	0	0	0	0	0	5.61	0	0	0	0	0	1.39	1.36	0
**S1-B-CTRL**	0	0.15	0	0	0	0	0	10.47	0	0	0	0	0	1.17	2.6	0
**S1-C-CTRL**	0	0.26	0	0	0	0	0	5.51	0	0	0	0	0	1.01	1.61	0.3
**S1-A-PPC**	0	0.06	0	0	0	0	0	1.29	0	0	0	0	0	4.63	1.35	0.18
**S1-B-PPC**	0	0	0	0	0	0	0	1.58	0	0	0	0	0	6.32	1.8	0.21
**S1-C-PPC**	0	0.07	0	0	0	0	0	1.36	0	0	0	0	0	9.12	1.99	0.21
**S1-A-PPI**	0	0.13	0	0	0	0	0	1.1	0	0	0	0	0	1.54	0.67	0
**S1-B-PPI**	0	0.18	0	0	0	0	0	0.95	0	0	0	0	0	1.31	0.53	0
**S1-C-PPI**	0	0.17	0	0	0	0	0	0.93	0	0	0	0	0	0.69	0.3	0
**S2-A-CTRL**	0	0	0	0	0	0	0	0.22	0.18	0.39	0	0	0	24.22	4.35	0.49
**S2-B-CTRL**	0	0	0	0	0	0	0	0.18	0.08	0.24	0	0	0	12.54	2.54	0.55
**S2-C-CTRL**	0	0	0	0	0	0.01	0	0.17	0.14	0.27	0	0	0	18.39	3.26	0.42
**S2-A-PPC**	0	0	0	0	0	0	0	0.18	0.21	1.19	0	0.03	0	24.75	4.95	0.96
**S2-B-PPC**	0	0	0	0	0	0	0	0.17	0.21	1.71	0.03	0.03	0	30.2	5.95	1.04
**S2-C-PPC**	0	0	0	0	0	0	0	0.11	0.25	1.29	0	0	0	31.14	5.74	0.74
**S2-A-PPI**	0	0	0	0	0	0	0	0.2	0.09	0.38	0	0	0	12.02	2.55	0.25
**S2-B-PPI**	0	0	0	0	0	0	0	0.15	0.11	0.32	0	0	0	15.59	2.8	0.3
**S2-C-PPI**	0	0	0	0	0	0	0	0.15	0.1	0.32	0	0	0	11.54	2.27	0.24
**S3-A-CTRL**	0	1.21	0	0	0	0	0	8.57	0.53	2.08	0	0	0	1.8	1.06	0.23
**S3-B-CTRL**	0	0.42	0	0	0	0	0	12.64	0.58	2.64	0	0	0	1.52	1.07	0.31
**S3-C-CTRL**	0	0.31	0	0	0	0	0	11.4	0.9	3.1	0	0	0	1.66	1.27	0
**S3-A-PPC**	0	0.01	0	0	0.03	0	0	10.14	0.64	3.45	0.04	0	0	4.94	1.85	0.16
**S3-B-PPC**	0	0.01	0	0	0.03	0	0	10.54	0.69	3.35	0.03	0.03	0	5.23	1.81	0.2
**S3-C-PPC**	0	0	0	0	0.03	0	0	9.88	0.6	4.62	0.04	0.03	0	5.22	2.03	0.18
**S3-A-PPI**	0	0.12	0	0	0	0	0	13.31	0.76	1.11	0	0	0	3.8	1.74	0
**S3-B-PPI**	0	0	0	0	0	0	0	8.81	0.55	4.39	0	0	0	4.46	2.07	0.16
**S3-C-PPI**	0	0.07	0	0	0	0	0	9.35	0.63	4.43	0	0	0	3.9	1.68	0.3
**S4-A-CTRL**	0.1	10.74	0	0	0	0	0	0	0	0	0	0	0	0.36	0.08	0
**S4-B-CTRL**	0.09	10.71	0.03	0	0	0	0	0	0	0	0	0	0	0.3	0	0
**S4-C-CTRL**	0.08	9.19	0	0	0	0	0	0	0	0	0	0	0	0.26	0	0
**S4-A-PPC**	0	0.59	0	0	0	0	0	0.77	0	0	0	0	0	6.06	1.96	0
**S4-B-PPC**	0	0.94	0	0	0	0	0	0	0	0	0	0	0	6.92	1.41	0
**S4-C-PPC**	0	0.99	0	0	0	0	0	0.63	0	0	0	0	0	5.03	0.63	0
**S4-A-PPI**	0	1.83	0	0	0	0	0	0.16	0	0	0	0	0	0.92	0.19	0
**S4-B-PPI**	0	2.83	0	0	0	0	0	0.1	0	0	0	0	0	0.81	0.11	0
**S4-C-PPI**	0	2.15	0	0	0	0	0	0.4	0	0	0	0	0	1.55	0.37	0
**S5-A-CTRL**	1.2	0.37	0.31	0	0	0	0	0	0	8.73	0	0	0	3.58	1.84	0
**S5-B-CTRL**	1.43	0.22	0.22	0	0	0	0	0.6	0	7.18	0	0	0	3.37	1.94	0
**S5-C-CTRL**	1.04	0.33	0	0	0	0	0	0.84	0	8.77	0	0	0	3.53	2.42	0
**S5-A-PPC**	0	0	0	0	0	0	0	1.45	0	5.99	0	0	0	17.38	5.54	0
**S5-B-PPC**	0	0	0	0	0	0	0	2.19	0.42	6.85	0	0	0	20.26	6.88	0
**S5-C-PPC**	0	0	0	0	0	0	0	1.46	0	4.33	0	0	0	17.71	4.79	0
**S5-A-PPI**	0.36	0	0	0	0	0	0	1.24	0	6.39	0	0	0	13.29	3.69	0
**S5-B-PPI**	0.32	0	0	0	0	0	0	1.73	0.35	5.97	0	0	0	9.16	3.15	0
**S5-C-PPI**	0.24	0	0	0	0	0	0	0.86	0	4.33	0	0	0	8.12	2.82	0
**S6-A-CTRL**	0	0.27	0	0	0	0	0	0.38	0.32	0	0	0	0.43	0.78	0.3	0
**S6-B-CTRL**	0	0.19	0	0	0	0	0	0.26	0.34	0	0	0	0.75	0.76	0.39	0
**S6-C-CTRL**	0	0.2	0	0	0	0	0	0.25	0.24	0	0	0	0.4	0.64	0.48	0
**S6-A-PPC**	0	0.04	0	0	0	0	0	0.93	0.14	0.66	0	0	0	9.42	1.34	0
**S6-B-PPC**	0	0.03	0	0	0	0	0	1.11	0.19	0.81	0	0	0	11.16	1.48	0
**S6-C-PPC**	0	0.04	0	0	0	0	0	1.24	0.2	1.25	0	0	0	14.62	1.81	0
**S6-A-PPI**	0	0.14	0	0	0	0	0	1.01	0.14	0.62	0	0	0	3.33	0.75	0
**S6-B-PPI**	0	0.1	0	0	0	0	0	1.2	0.15	0.91	0	0	0.13	4.03	0.86	0
**S6-C-PPI**	0	0.11	0	0	0	0	0	1.3	0.11	0.81	0	0	0	2.97	0.62	0

**Bacterial species**	Bifidobacterium ruminantium	Bilophila below species threshold	Bilophila wadsworthia	Blautia acetigignens	Blautia below species threshold	Blautia caecimuris	Blautia fusiformis	Blautia hansenii	Blautia intestinalis	Blautia multihit species	Blautia obeum	Blautia segnis	Blautia wexlerae	Bovifimicola below species threshold	Bradyrhizobium below species threshold	Brotaphodocola below species threshold
**S1-A-CTRL**	0	0	0.3	0	0	0	0	0	0	0	0	0	0	0	0	0
**S1-B-CTRL**	0	0	0	0	0	0	0	0	0	0	0	0	0	0	0	0
**S1-C-CTRL**	0	0	0	0	0	0	0	0	0	0	0	0	0	0	0	0
**S1-A-PPC**	0	0	0	0	0.1	0	0	0	0	0	0	0	0	0	0	0
**S1-B-PPC**	0	0	0	0	0	0	0	0	0	0	0	0	0	0	0	0
**S1-C-PPC**	0	0	0	0	0	0	0	0	0	0	0	0	0	0	0	0
**S1-A-PPI**	0	0	0	0	0	0	0	0	0	0	0	0	0	0	0	0
**S1-B-PPI**	0	0	0	0	0	0	0	0	0	0	0	0	0	0	0	0
**S1-C-PPI**	0	0	0	0	0	0.09	0	0	0	0	0	0	0	0	0	0
**S2-A-CTRL**	0	0	0.03	0	0.17	0	0.03	0	0	0.03	0	0	0.02	0	0	0
**S2-B-CTRL**	0	0	0.02	0	0.1	0	0.02	0	0	0.02	0.02	0	0.02	0	0	0
**S2-C-CTRL**	0	0	0.02	0	0.14	0	0.02	0	0	0.02	0	0	0.02	0	0	0
**S2-A-PPC**	0	0	0.02	0	0.27	0	0.04	0	0	0.04	0.03	0	0.05	0	0	0
**S2-B-PPC**	0	0	0.01	0	0.22	0	0.03	0	0	0.03	0.02	0	0.04	0	0	0
**S2-C-PPC**	0	0	0	0	0.21	0	0.03	0	0	0.02	0.03	0	0.04	0	0	0
**S2-A-PPI**	0	0	0.02	0	0.24	0	0.04	0	0	0.05	0.03	0	0.05	0	0	0
**S2-B-PPI**	0	0	0	0	0.19	0	0.03	0	0	0.05	0.02	0	0.05	0	0	0
**S2-C-PPI**	0	0	0.02	0	0.31	0	0.02	0	0	0.08	0.04	0	0.06	0	0	0
**S3-A-CTRL**	0	0	0.12	0	0	0	0	0	0	0	0	0	0	0	0	0
**S3-B-CTRL**	0	0	0	0	0	0	0	0	0	0	0	0	0	0	0	0
**S3-C-CTRL**	0	0	0	0	0	0	0	0	0	0	0	0	0	0	0	0
**S3-A-PPC**	0.05	0	0	0	0.08	0	0.02	0	0	0.02	0	0	0	0	0	0
**S3-B-PPC**	0.05	0	0	0	0.07	0	0	0	0	0	0	0	0	0	0	0
**S3-C-PPC**	0.06	0	0	0	0.09	0	0.03	0	0	0.02	0	0	0	0	0	0
**S3-A-PPI**	0	0	0	0	0.41	0	0	0	0	0	0	0	0	0	0	0
**S3-B-PPI**	0	0	0	0	0.29	0	0	0	0	0.07	0	0	0	0	0	0
**S3-C-PPI**	0	0	0	0	0.14	0	0	0	0	0	0	0	0	0	0	0
**S4-A-CTRL**	0	0	0.04	0	0.08	0	0	0	0	0	0	0	0	0	0	0
**S4-B-CTRL**	0	0	0.06	0	0.06	0	0	0	0	0	0	0	0	0	0	0
**S4-C-CTRL**	0	0	0	0	0.07	0	0	0	0	0	0	0	0	0	0	0
**S4-A-PPC**	0	0	0.25	0	0.33	0	0	0	0	0	0	0	0	0	0	0
**S4-B-PPC**	0	0	0	0	0	0	0	0	0	0	0	0	0	0	0	0
**S4-C-PPC**	0	0	0	0	0.27	0	0	0	0	0	0	0	0	0	0	0
**S4-A-PPI**	0	0	0.05	0	0.08	0	0	0	0	0	0	0	0	0	0	0
**S4-B-PPI**	0	0	0	0	0.15	0	0	0	0	0	0	0	0	0	0	0
**S4-C-PPI**	0	0	0.04	0	0.08	0	0	0	0	0	0	0	0	0	0	0
**S5-A-CTRL**	0	0	0	0	0.31	0	0	0	0	0	0	0	0	0	0	0
**S5-B-CTRL**	0	0	0	0	0.34	0	0	0	0	0	0	0	0	0	0.2	0
**S5-C-CTRL**	0	0	0	0	0.53	0	0	0	0	0	0	0	0	0	0	0
**S5-A-PPC**	0	0	0	0	0.52	0	0	0	0	0.39	0	0	2.8	0	0	0
**S5-B-PPC**	0	0	0	0	0.24	0	0	0	0	0	0	0	1.09	0	0	0
**S5-C-PPC**	0	0	0	0	1.08	0	0	0	0	0.24	0	0	1.71	0	0	0
**S5-A-PPI**	0	0	0	0	0.26	0	0	0	0	0.21	0	0	0.25	0	0	0
**S5-B-PPI**	0	0	0	0	0.31	0	0	0	0	0	0	0	0.58	0	0	0
**S5-C-PPI**	0	0	0	0	0.34	0	0	0	0	0	0	0	0.32	0	0	0
**S6-A-CTRL**	0	0	0	0	0.25	0	0	0	0	0	0	0	0	0	0	0
**S6-B-CTRL**	0	0	0	0	0	0	0	0	0	0	0	0	0	0	0	0
**S6-C-CTRL**	0	0	0	0	0.19	0	0	0	0	0	0	0	0	0	0	0
**S6-A-PPC**	0	0	0	0	0.21	0	0.04	0	0	0.04	0.02	0	0.03	0	0	0
**S6-B-PPC**	0	0	0	0	0.18	0	0.06	0	0	0.03	0	0	0.03	0	0	0
**S6-C-PPC**	0	0	0	0.03	0.24	0	0.09	0	0	0.06	0	0.03	0.03	0	0	0
**S6-A-PPI**	0	0	0	0.06	0.19	0	0	0	0	0.08	0	0	0.06	0	0	0
**S6-B-PPI**	0	0	0	0	0.34	0	0	0	0	0.12	0	0	0.06	0	0	0
**S6-C-PPI**	0	0	0	0.06	0.29	0	0.05	0	0	0.12	0	0	0.08	0	0	0

**Bacterial species**	Brotocaccenecus below species threshold	Brotocaccenecus cirricatena	Butyribacter below species threshold	Butyribacter intestini	Butyricicoccus below species threshold	Butyricicoccus faecihominis	Butyricicoccus pullicaecorum	Butyricimonas below species threshold	Butyricimonas faecihominis	Butyricimonas multihit species	Butyricimonas paravirosa	Butyricimonas virosa	Butyrivibrio below species threshold	Caecibacteroides below species threshold	Caldicellulosiruptor below species threshold	Capnocytophaga below species threshold
**S1-A-CTRL**	0	0	0	0	0	0	0	0	0	0	0	0	0	0	0	0
**S1-B-CTRL**	0	0	0	0	0	0	0	0	0	0	0	0	0	0	0	0
**S1-C-CTRL**	0	0	0	0	0	0	0	0	0	0	0	0	0	0	0	0
**S1-A-PPC**	0	0	0	0	0	0	0	0	0	0	0	0	0	0	0	0
**S1-B-PPC**	0	0	0	0	0	0	0	0	0	0	0	0	0	0	0	0
**S1-C-PPC**	0	0	0	0	0	0	0	0	0	0	0	0	0	0	0	0
**S1-A-PPI**	0	0	0	0	0	0	0	0	0	0	0	0.16	0	0	0	0
**S1-B-PPI**	0	0	0	0	0	0	0	0	0	0	0	0	0	0	0	0
**S1-C-PPI**	0	0	0	0	0	0	0	0	0	0	0	0.13	0	0	0	0
**S2-A-CTRL**	0	0	0	0	0	0	0	0	0	0	0	0	0	0	0	0
**S2-B-CTRL**	0	0	0	0	0	0	0	0	0	0	0	0	0	0	0	0
**S2-C-CTRL**	0	0	0	0	0	0	0	0	0	0	0	0	0	0	0	0
**S2-A-PPC**	0.02	0	0.02	0	0	0	0	0	0	0	0	0	0	0	0	0
**S2-B-PPC**	0.02	0	0	0	0	0	0	0	0	0	0	0	0	0	0	0
**S2-C-PPC**	0.02	0	0.02	0	0	0	0	0	0	0	0	0	0	0	0	0
**S2-A-PPI**	0.05	0	0	0	0	0	0	0	0	0	0	0	0	0	0	0
**S2-B-PPI**	0	0	0	0	0	0	0	0	0	0	0	0	0	0	0	0
**S2-C-PPI**	0.02	0	0	0	0	0	0	0	0	0	0	0	0	0	0	0
**S3-A-CTRL**	0.27	0	0	0	0	0	0	0	0	0	0	0	0	0	0	0
**S3-B-CTRL**	0.3	0	0	0	0	0	0	0	0	0	0	0	0	0	0	0
**S3-C-CTRL**	0	0	0	0	0	0	0	0	0	0	0	0	0	0	0	0
**S3-A-PPC**	0.05	0	0	0	0	0	0	0	0	0	0	0	0	0	0	0
**S3-B-PPC**	0.03	0	0	0	0	0	0	0	0	0	0	0	0	0	0	0
**S3-C-PPC**	0.03	0	0	0	0	0	0	0	0	0	0	0	0	0	0	0
**S3-A-PPI**	0.13	0	0	0	0	0	0	0	0	0	0	0	0	0	0	0
**S3-B-PPI**	0.08	0	0	0	0	0	0	0	0	0	0	0	0	0	0	0
**S3-C-PPI**	0	0	0	0	0	0	0	0	0	0	0	0	0	0	0	0
**S4-A-CTRL**	0	0.06	0	0	0	0	0	0	0	0	0	0.05	0	0	0	0
**S4-B-CTRL**	0	0.12	0	0	0	0	0	0	0	0	0	0.06	0	0	0	0
**S4-C-CTRL**	0	0	0	0	0	0	0	0	0	0	0	0.09	0	0	0	0
**S4-A-PPC**	0	0	0	0	0	0	0	0	0	0	0	0	0	0	0	0
**S4-B-PPC**	0	0.34	0	0	0	0	0	0	0	0	0	0	0	0	0	0
**S4-C-PPC**	0.28	0.4	0	0	0	0	0	0	0	0	0	0	0	0	0	0
**S4-A-PPI**	0	0.18	0	0	0	0	0	0	0	0	0	0.17	0	0	0	0
**S4-B-PPI**	0	0.13	0	0	0	0	0	0	0	0	0	0.08	0	0	0	0
**S4-C-PPI**	0	0.18	0	0	0	0	0	0	0	0	0	0.06	0	0	0	0
**S5-A-CTRL**	0	0	0	0	0	0	0	0	0	0	0.31	0	0	0	0	0
**S5-B-CTRL**	0	0	0	0	0	0	0	0	0	0	0	0	0	0	0	0
**S5-C-CTRL**	0	0	0	0	0	0	0	0	0	0	0	0	0	0	0	0
**S5-A-PPC**	0	0	0	0	0	0	0	0	0	0	0	0	0	0	0	0
**S5-B-PPC**	0	0	0	0	0	0	0	0	0	0	0	0	0	0	0	0
**S5-C-PPC**	0	0	0	0	0	0	0	0	0	0	0	0	0	0	0	0
**S5-A-PPI**	0	0	0	0	0	0	0	0	0	0	0	0	0	0	0	0
**S5-B-PPI**	0	0	0	0	0	0	0	0	0	0	0	0	0	0	0	0
**S5-C-PPI**	0	0	0	0	0	0	0	0	0	0	0	0	0	0	0	0
**S6-A-CTRL**	0.23	0	0	0	0	0	0	0	0	0	0	0	0	0	0	0
**S6-B-CTRL**	0.33	0	0	0	0	0	0	0	0	0	0	0	0	0	0	0
**S6-C-CTRL**	0.16	0	0	0	0	0	0	0	0	0	0	0	0	0	0	0
**S6-A-PPC**	0.06	0	0	0	0	0	0	0	0	0	0	0	0	0	0	0
**S6-B-PPC**	0.07	0	0	0	0	0	0	0	0	0	0	0	0	0	0	0
**S6-C-PPC**	0.07	0	0	0	0	0	0	0	0	0	0	0	0	0	0	0
**S6-A-PPI**	0.06	0	0	0	0	0	0	0	0	0	0	0	0	0	0	0
**S6-B-PPI**	0.15	0	0	0	0	0	0	0	0	0	0	0	0	0	0	0
**S6-C-PPI**	0.2	0	0	0	0	0	0	0	0	0	0	0	0	0	0	0

**Bacterial species**	Caproicibacterium below species threshold	Caproiciproducens below species threshold	Carnobacterium below species threshold	Cedecea davisae	Cetobacterium below species threshold	Chitinophaga below species threshold	Christensenella below species threshold	Citrobacter below species threshold	Citrobacter braakii	Citrobacter enshiensis	Citrobacter freundii	Citrobacter koseri	Citrobacter multihit species	Citrobacter portucalensis	Clavelimonas below species threshold	Clostridium below species threshold
**S1-A-CTRL**	0	0	0	0	0	0	0	0	0	0	0	0.23	0	0	0	0
**S1-B-CTRL**	0	0	0	0	0	0	0	0	0	0	0	0	0	0	0	0
**S1-C-CTRL**	0	0	0	0	0	0	0	0	0	0	0	0.15	0	0	0	0
**S1-A-PPC**	0	0	0	0	0	0	0	0	0	0	0	0.11	0	0	0	0
**S1-B-PPC**	0	0	0	0	0	0	0	0	0	0	0	0.13	0	0	0	0
**S1-C-PPC**	0	0	0	0	0	0	0	0	0	0	0	0	0	0	0	0.14
**S1-A-PPI**	0	0	0	0	0.53	0	0	0	0	0	0	0	0	0	0	0.46
**S1-B-PPI**	0	0	0	0	0.36	0	0	0.16	0	0	0	0.21	0	0	0	0.18
**S1-C-PPI**	0	0	0	0	0.58	0	0	0	0	0	0	0.07	0	0	0	0.32
**S2-A-CTRL**	0	0	0	0	0	0	0	0.04	0	0	0.05	0	0.1	0.25	0	0
**S2-B-CTRL**	0	0	0	0	0	0	0	0.05	0	0.01	0.08	0.01	0.2	0.96	0	0.01
**S2-C-CTRL**	0	0	0	0	0	0	0	0.04	0	0	0.08	0.01	0.11	0.01	0	0.02
**S2-A-PPC**	0	0	0	0	0	0	0	0.02	0	0	0.06	0	0.08	0.07	0	0.04
**S2-B-PPC**	0	0	0	0	0	0	0	0.04	0	0	0.05	0	0.12	0.2	0	0.03
**S2-C-PPC**	0	0	0	0.01	0	0	0	0.01	0	0	0.04	0	0.14	0.29	0	0.02
**S2-A-PPI**	0	0	0	0	0	0	0	0.03	0	0	0.07	0	0.23	0.48	0	0.02
**S2-B-PPI**	0	0	0	0	0	0	0	0.02	0	0	0.09	0.01	0.18	0.51	0	0.02
**S2-C-PPI**	0	0	0	0.01	0	0	0	0.03	0.01	0	0.1	0	0.19	0.23	0	0.02
**S3-A-CTRL**	0	0	0	0	0	0	0	0	0	0	0.1	0	0.39	0	0	0
**S3-B-CTRL**	0	0	0	0	0	0	0	0	0	0	0	0	0.28	0.32	0	0
**S3-C-CTRL**	0	0	0	0	0	0	0	0	0	0	0	0	0.29	0.13	0	0
**S3-A-PPC**	0	0	0	0	0	0	0	0	0	0	0	0	0.04	0.01	0	0.02
**S3-B-PPC**	0	0	0	0	0	0	0	0	0	0	0	0	0.05	0.01	0	0.02
**S3-C-PPC**	0	0	0	0	0	0	0	0	0	0	0	0	0.04	0	0	0.02
**S3-A-PPI**	0	0	0	0	0	0	0	0	0	0	0	0	0.16	0.1	0	0
**S3-B-PPI**	0	0	0	0	0	0	0	0	0	0	0	0	0.12	0.06	0	0.06
**S3-C-PPI**	0	0	0	0	0	0	0	0	0	0	0.07	0	0.16	0.08	0	0
**S4-A-CTRL**	0	0	0	0	0	0	0	0	0	0	0	0	0.04	0.11	0	0
**S4-B-CTRL**	0	0	0	0	0	0	0	0	0	0	0	0	0.06	0.07	0	0
**S4-C-CTRL**	0	0	0	0	0	0	0	0	0	0	0	0	0.04	0.07	0	0
**S4-A-PPC**	0	0	0	0	0	0	0	0	0	0	0	0	0.28	0.66	0	0
**S4-B-PPC**	0	0	0	0	0	0	0	0	0	0	0	0	0.37	0.65	0	0
**S4-C-PPC**	0	0	0	0	0	0	0	0	0	0	0	0	0.39	0.63	0	0
**S4-A-PPI**	0	0	0	0	0	0	0	0	0	0	0	0	0.06	0.1	0	0.06
**S4-B-PPI**	0	0	0	0	0	0	0	0	0	0	0	0	0.03	0.14	0	0
**S4-C-PPI**	0	0	0	0	0	0	0	0	0	0	0	0	0	0.07	0	0
**S5-A-CTRL**	0	0	0	0	0	0	0	0	0	0	0	0	0	0	0	0
**S5-B-CTRL**	0	0	0	0	0	0	0	0	0	0	0	0	0	0	0	0
**S5-C-CTRL**	0	0	0	0	0	0	0	0	0	0	0	0	0	0	0	0
**S5-A-PPC**	0	0	0	0	0	0	0	0	0	0	0	0	0	0	0	0
**S5-B-PPC**	0	0	0	0	0	0	0	0	0	0	0	0	0	0	0	0
**S5-C-PPC**	0	0	0	0	0	0	0	0	0	0	0	0	0	0	0	0
**S5-A-PPI**	0	0	0	0	0	0	0	0	0	0	0	0	0	0	0	0
**S5-B-PPI**	0	0	0	0	0	0	0	0	0	0	0	0	0	0	0	0
**S5-C-PPI**	0	0	0	0	0	0	0	0	0	0	0	0	0	0	0	0.14
**S6-A-CTRL**	0	0	0	0	0	0	0	0	0	0	0	0	0	0	0	0
**S6-B-CTRL**	0	0	0	0	0	0	0	0	0	0	0	0	0.2	0	0	0
**S6-C-CTRL**	0	0	0	0	0	0	0	0	0	0	0	0	0.14	0	0	0
**S6-A-PPC**	0	0	0.1	0	0	0	0	0	0	0	0	0	0	0	0	0
**S6-B-PPC**	0	0	0.06	0	0	0	0	0	0	0	0	0	0	0	0	0
**S6-C-PPC**	0	0	0.08	0	0	0	0	0	0	0	0	0	0	0	0	0
**S6-A-PPI**	0	0	0	0	0	0	0	0	0	0	0	0	0.05	0	0	0.05
**S6-B-PPI**	0	0	0.1	0	0	0	0	0	0	0	0	0	0.07	0	0	0
**S6-C-PPI**	0	0	0.1	0	0	0	0	0	0	0	0	0	0	0	0	0

**Bacterial species**	[Clostridium] below species threshold	Clostridium fessum	Clostridium multihit species	Clostridium phoceensis	Clostridium segne	Clostridium tertium	Cohnella below species threshold	Collinsella aerofaciens	Collinsella below species threshold	Congzhengia below species threshold	Coprobacter below species threshold	Coprobacter fastidiosus	Coprobacter secundus	Coprococcus aceti	Coprococcus ammoniilyticus	Coprococcus below species threshold
**S1-A-CTRL**	0	0	0	0	0	0	0	0	0	0	0	0	0	0	0	0
**S1-B-CTRL**	0	0	0	0	0	0	0	0	0	0	0	0	0	0	0	0
**S1-C-CTRL**	0	0	0	0.23	0	0	0	0	0	0	0	0	0	0	0	0
**S1-A-PPC**	0	0	0	0	0	0	0	0.25	0	0	0	0	0	0	0	0.12
**S1-B-PPC**	0	0	0	0	0	0	0	0	0	0	0	0	0	0	0	0
**S1-C-PPC**	0	0	0	0	0	0	0	0	0	0	0	0	0	0	0	0
**S1-A-PPI**	0	0	0	0	0	0	0	1.12	0	0	0	0	0	0	0	0
**S1-B-PPI**	0	0	0	0	0	0	0	0	0	0	0	0	0	0	0	0
**S1-C-PPI**	0	0	0	0	0	0	0	1.13	0	0	0	0	0	0	0	0
**S2-A-CTRL**	0	0	0	0.05	0	0	0	0	0	0	0	0	0	0	0	0
**S2-B-CTRL**	0	0	0	0.03	0	0	0	0	0	0	0	0	0	0	0	0
**S2-C-CTRL**	0	0	0	0.03	0	0	0	0	0	0	0	0	0	0	0	0
**S2-A-PPC**	0.02	0	0	0.08	0	0	0	0	0	0	0	0	0	0	0	0
**S2-B-PPC**	0	0.02	0	0.06	0	0	0	0	0	0	0	0	0	0	0	0.02
**S2-C-PPC**	0	0	0	0.02	0	0	0	0	0	0	0	0	0	0	0	0.03
**S2-A-PPI**	0	0	0	0.05	0	0	0	0	0	0	0	0	0	0	0	0.03
**S2-B-PPI**	0	0	0	0.07	0	0	0	0	0	0	0	0	0	0	0	0.03
**S2-C-PPI**	0	0.03	0	0.06	0	0	0	0	0	0	0	0	0	0	0	0.02
**S3-A-CTRL**	0	0	0	0	0	0	0	0.3	0	0	0	0	0	0	0	0
**S3-B-CTRL**	0	0	0	0	0	0	0	0.35	0	0	0	0	0	0	0	0
**S3-C-CTRL**	0	0	0	0	0	0	0	0.39	0	0	0	0	0	0	0	0
**S3-A-PPC**	0	0	0	0	0	0	0	0.47	0.04	0	0	0	0	0	0.16	0
**S3-B-PPC**	0	0	0	0	0	0	0	0.47	0.04	0	0	0	0	0	0.16	0
**S3-C-PPC**	0	0	0	0	0	0	0	0.49	0.03	0	0	0	0	0	0.13	0
**S3-A-PPI**	0	0	0	0	0	0.1	0	0.65	0	0	0	0	0	0	0.22	0
**S3-B-PPI**	0	0	0	0	0	0	0	0.53	0	0	0	0	0	0	0.23	0
**S3-C-PPI**	0	0	0	0	0	0	0	0.7	0	0	0	0	0	0	0.22	0
**S4-A-CTRL**	0	0	0	0	0	0	0	0	0	0	0.06	0	0.05	0	0	0
**S4-B-CTRL**	0	0	0	0	0	0	0	0	0	0	0	0	0	0	0	0
**S4-C-CTRL**	0	0	0	0	0	0.05	0	0	0	0	0.07	0	0	0	0	0
**S4-A-PPC**	0	0	0	0	0	0	0	1.09	0	0	0	0	0	0	0	0
**S4-B-PPC**	0	0	0	0	0	0	0	2.72	0	0	0	0	0	0	0	0
**S4-C-PPC**	0	0	0	0	0	0	0	0.81	0	0	0.25	0	0	0	0	0
**S4-A-PPI**	0	0	0	0	0	0	0	0	0	0	0.08	0	0.06	0	0	0
**S4-B-PPI**	0	0	0	0	0	0	0	0	0	0	0	0	0.04	0	0	0
**S4-C-PPI**	0	0	0	0	0	0	0	0	0	0	0.07	0	0	0	0	0
**S5-A-CTRL**	0	0	0	0	0	0	0	0	0	0	0	0	0	0.42	0	0
**S5-B-CTRL**	0	0	0	0	0	0	0	0	0	0	0	0	0	0.49	0	0
**S5-C-CTRL**	0	0	0	0	0	0	0	0	0	0	0	0	0	0.6	0	0
**S5-A-PPC**	0	0	0	0	0	0	0	0.39	0	0	0	0	0	3.01	0	0
**S5-B-PPC**	0	0	0	0	0	0	0	0.74	0	0	0	0	0	2.45	0	0
**S5-C-PPC**	0	0	0	0	0	0	0	0.36	0	0	0	0	0	3.67	0	0.25
**S5-A-PPI**	0	0	0	0	0	0	0	0.3	0	0	0	0	0	0.51	0	0
**S5-B-PPI**	0	0	0	0	0	0	0	0.32	0	0	0	0	0	1.05	0	0
**S5-C-PPI**	0	0	0	0	0	0.13	0	0.27	0	0	0	0	0	0.4	0	0
**S6-A-CTRL**	0	0	0	0	0	0	0	0	0	0	0	0	0	0	0	0
**S6-B-CTRL**	0	0	0	0	0	0	0	0	0	0	0	0	0	0	0	0
**S6-C-CTRL**	0	0	0	0	0	0	0	0	0	0	0	0	0	0	0	0
**S6-A-PPC**	0	0	0	0	0	0	0	0	0	0	0	0	0	0.03	0.03	0
**S6-B-PPC**	0	0	0	0	0	0	0	0	0	0	0	0	0	0	0.03	0
**S6-C-PPC**	0	0	0	0	0	0	0	0	0	0	0	0	0	0	0	0
**S6-A-PPI**	0	0	0	0	0	0	0	0	0	0	0	0	0	0	0	0
**S6-B-PPI**	0	0	0	0	0	0	0	0	0	0	0	0	0	0	0.15	0
**S6-C-PPI**	0	0	0	0	0	0	0	0	0	0	0	0	0	0	0.09	0.05

**Bacterial species**	Coprococcus catus	Coprococcus comes	Coprococcus eutactus	Coprococcus intestinihominis	Coprococcus multihit species	Coprococcus phoceensis	Corynebacterium below species threshold	Cronobacter below species threshold	Cutibacterium acnes	Cutibacterium below species threshold	Cutibacterium multihit species	Cutibacterium namnetense	Dakarella below species threshold	Dakarella massiliensis	Desulfovibrio below species threshold	Desulfovibrio piger
**S1-A-CTRL**	0	0	0	0	0	1.76	0	0	0	0	0	0	0	0	0	0.52
**S1-B-CTRL**	0	0	0	0	0	1.04	0	0	0.48	0	0	0	0	0	0	0.74
**S1-C-CTRL**	0	0	0	0	0	2.21	0	0	1.6	0	0	0	0	0	0	0.79
**S1-A-PPC**	0	0	0	0	0	1.73	0	0	0.64	0	0	0	0	0	0	0
**S1-B-PPC**	0	0	0	0	0	2.6	0	0	0.25	0	0	0	0	0	0	0
**S1-C-PPC**	0	0	0	0	0	1.82	0	0	1.86	0	0	0	0	0	0	0.28
**S1-A-PPI**	0	0	0	0	0	1.61	0	0	0.26	0	0	0	0	0	0	0
**S1-B-PPI**	0	0	0	0	0	3	0	0	0.27	0	0	0	0	0	0	0.27
**S1-C-PPI**	0	0	0	0	0	1.84	0	0	0.19	0	0	0	0	0	0	0.12
**S2-A-CTRL**	0	0.02	0	0	0	0	0	0	0.16	0	0	0	0	0	0	0
**S2-B-CTRL**	0	0.02	0	0	0	0	0	0	0.18	0	0	0	0	0	0	0
**S2-C-CTRL**	0	0.04	0	0	0	0	0	0	0.1	0	0	0	0	0	0	0
**S2-A-PPC**	0	0.04	0	0	0	0	0	0	0.88	0.03	0.03	0.05	0	0	0	0
**S2-B-PPC**	0	0.06	0	0	0	0	0	0	0.28	0	0	0	0	0	0	0
**S2-C-PPC**	0	0.07	0	0	0	0	0	0	0.11	0	0	0	0	0	0	0
**S2-A-PPI**	0	0.04	0	0	0	0	0	0	0.2	0	0	0	0	0	0	0
**S2-B-PPI**	0	0.06	0	0	0	0	0	0	0.22	0	0	0	0	0	0	0
**S2-C-PPI**	0	0.04	0	0	0	0	0	0	0.13	0	0	0	0	0	0	0
**S3-A-CTRL**	0	0.23	0	0	0	0	0	0	0.71	0	0	0	0	0	0	0
**S3-B-CTRL**	0	0.28	0	0	0	0	0	0	0.74	0	0	0	0	0	0	0
**S3-C-CTRL**	0	0.25	0	0	0	0	0	0	0.99	0	0	0	0	0	0	0
**S3-A-PPC**	0	0.1	0	0	0	0	0	0	0.36	0	0	0	0	0	0	0
**S3-B-PPC**	0	0.11	0	0	0	0	0	0	0.23	0	0	0	0	0	0	0
**S3-C-PPC**	0	0.09	0	0	0	0	0	0	0.35	0	0	0	0	0	0	0
**S3-A-PPI**	0	0.42	0	0	0	0	0	0	0.64	0	0	0	0.17	0.5	0	0
**S3-B-PPI**	0	0.37	0	0	0	0	0	0	0.63	0	0	0	0	0	0	0
**S3-C-PPI**	0	0.3	0	0	0	0	0	0	1.55	0	0	0	0	0.19	0	0
**S4-A-CTRL**	0	0	0	0	0	0	0	0	0.53	0	0	0	0	0	0	0
**S4-B-CTRL**	0	0	0	0	0	0	0	0	0.63	0	0	0	0	0.09	0	0
**S4-C-CTRL**	0	0	0	0	0	0	0	0	0.1	0	0	0	0.07	0.3	0	0
**S4-A-PPC**	0	0	0	0	0	0	0	0	5.46	0	0	0	0	0.66	0	0
**S4-B-PPC**	0	0	0	0	0	0	0	0	1.98	0	0	0	0.38	0.57	0	0
**S4-C-PPC**	0	0	0	0	0	0	0	0	1.7	0	0	0	0.46	0.96	0	0
**S4-A-PPI**	0	0	0	0	0	0	0	0	0.14	0	0	0	0.11	0.36	0	0
**S4-B-PPI**	0	0	0	0	0	0	0	0	0.33	0	0	0	0.32	0.6	0	0
**S4-C-PPI**	0	0	0	0	0	0	0	0	2.31	0.09	0	0	0	0.18	0	0
**S5-A-CTRL**	0	0	0	0	0	0	0	0	0	0	0	0	0	0	0	0
**S5-B-CTRL**	0	0	0	0	0	0	0	0	10.23	0	0	0	0	0	0	0
**S5-C-CTRL**	0	0	0	0	0	0	0	0	5.48	0	0	0	0	0	0	0
**S5-A-PPC**	0	0	0	0	0	0	0	0	2.19	0	0	0	0	0	0	0
**S5-B-PPC**	0	0	0	0	0	0	0	0	1.21	0	0	0	0	0	0	0
**S5-C-PPC**	0	0.27	0	0	0	0	0	0	0.32	0	0	0	0	0	0	0
**S5-A-PPI**	0	0	0	0	0	0	0	0	5.47	0	0	0	0	0	0	0
**S5-B-PPI**	0	0	0	0	0	0	0	0	0.43	0	0	0	0	0	0	0
**S5-C-PPI**	0	0.19	0	0	0	0	0	0	1.87	0	0	0	0	0	0	0
**S6-A-CTRL**	0	0	0	0	0	0	0	0	0.25	0	0	0	0	0	0	0
**S6-B-CTRL**	0	0	0	0	0	0	0	0.14	0.48	0	0	0	0	0	0	0
**S6-C-CTRL**	0	0	0	0	0	0	0	0	0.67	0	0	0	0	0	0	0
**S6-A-PPC**	0	0.05	0	0	0	0	0	0	0.03	0	0	0	0	0	0	0
**S6-B-PPC**	0	0	0	0	0	0	0	0	0.07	0	0	0	0	0	0	0
**S6-C-PPC**	0	0.03	0	0	0	0	0	0	0.05	0	0	0	0	0	0	0
**S6-A-PPI**	0	0.06	0	0	0	0	0	0	0.17	0	0	0	0	0	0	0
**S6-B-PPI**	0	0.1	0	0	0	0	0	0	0.23	0	0	0	0	0	0	0
**S6-C-PPI**	0	0.06	0	0	0	0	0	0	0.22	0	0	0	0	0	0	0

Bacterial species	Dialister below species threshold	Dialister hominis	Dialister invisus	Dialister massiliensis	Dialister multihit species	Dialister succinatiphilus	Dielma fastidiosa	Diplocloster below species threshold	Dorea ammoniilytica	Dorea amylophila	Dorea below species threshold	Dorea formicigenerans	Dorea longicatena	Dorea multihit species	Duncaniella below species threshold	Dysgonomonas below species threshold
**S1-A-CTRL**	0	0	0	0	0	0	0	0	0	0	0	0	0	0	0	0
**S1-B-CTRL**	0	0	0	0	0	0	0	0	0	0	0	0	0	0	0	0
**S1-C-CTRL**	0	0	0	0	0	0	0	0	0	0.4	0	0	0	0	0	0
**S1-A-PPC**	0	0	0	0	0	0	0	0	0	0.95	0	0	0	0.34	0	0
**S1-B-PPC**	0	0	0	0	0	0	0	0	0	0.6	0	0	0	0.24	0	0
**S1-C-PPC**	0	0	0	0	0	0	0	0	0	0.48	0	0	0	0.34	0	0
**S1-A-PPI**	0	0	0	0	0	0	0	0	0	0.6	0	0	0	0.4	0	0
**S1-B-PPI**	0	0	0	0	0	0	0	0	0	0.79	0	0	0	0.3	0	0
**S1-C-PPI**	0	0	0	0	0	0	0	0	0	0.47	0	0	0.12	0.39	0	0
**S2-A-CTRL**	0.05	0	0.84	0	0	0	0	0	0	0	0	0	0.12	0.06	0	0
**S2-B-CTRL**	0.03	0	0.77	0	0	0	0	0	0	0	0	0	0.03	0.04	0	0
**S2-C-CTRL**	0.03	0	0.81	0	0	0	0	0	0	0	0	0	0.09	0.05	0	0
**S2-A-PPC**	0.05	0	1.39	0	0	0	0	0	0.03	0	0.04	0.03	0.46	0.15	0	0
**S2-B-PPC**	0.06	0	1.4	0	0	0	0	0	0	0	0.03	0.02	0.33	0.12	0	0
**S2-C-PPC**	0.06	0	1.28	0	0	0	0	0	0	0	0.03	0.02	0.35	0.15	0	0
**S2-A-PPI**	0.06	0	0.7	0	0	0	0	0	0	0	0.04	0.03	0.26	0.12	0	0
**S2-B-PPI**	0	0	0.76	0	0	0	0	0	0	0	0.03	0	0.37	0.19	0	0
**S2-C-PPI**	0.03	0	0.88	0	0	0	0	0	0	0	0.02	0.02	0.4	0.17	0	0
**S3-A-CTRL**	0	0.49	0	0.4	2.38	0	0	0	0	0	0	0	0	0	0	0
**S3-B-CTRL**	0	0	0	0	1.19	0	0	0	0	0	0	0	0	0	0	0
**S3-C-CTRL**	0	0.72	0	0.52	2.85	0	0	0	0	0	0	0	0	0	0	0
**S3-A-PPC**	0	0	0	0	0.07	0	0	0	0	0	0	0	0.09	0.02	0	0
**S3-B-PPC**	0	0	0	0	0.07	0	0	0	0	0	0	0	0.06	0.03	0	0
**S3-C-PPC**	0	0	0	0	0.05	0	0	0	0	0	0	0	0.06	0.02	0	0
**S3-A-PPI**	0	0.29	0	0	0.96	0	0	0	0	0	0	0	0.27	0	0	0
**S3-B-PPI**	0	0	0	0	0.42	0	0	0	0	0	0	0	0.1	0	0	0
**S3-C-PPI**	0	0.17	0	0	0.43	0	0	0	0	0	0	0	0.12	0	0	0
**S4-A-CTRL**	0	0	0.23	0	0	0	0.1	0	0	0	0	0	0	0	0	0
**S4-B-CTRL**	0	0	0.36	0	0	0	0.12	0	0	0	0	0	0	0	0	0
**S4-C-CTRL**	0	0	0.42	0	0	0	0.13	0	0	0	0	0	0	0	0	0
**S4-A-PPC**	0	0	0	0	0	0	0	0	0	0	0	0	0	0	0	0
**S4-B-PPC**	0	0	0	0	0	0	0	0	0	0	0	0	0	0	0	0
**S4-C-PPC**	0	0	0.49	0	0	0	0	0	0	0	0	0	0	0	0	0
**S4-A-PPI**	0	0	0.14	0	0	0	0	0	0	0	0	0	0	0	0	0
**S4-B-PPI**	0	0	0	0	0	0	0	0	0	0	0	0	0	0	0	0
**S4-C-PPI**	0	0	0.1	0	0	0	0	0	0	0	0	0	0	0	0	0
**S5-A-CTRL**	0	0	3.48	0	0	0	0	0	0	0	0	0	0	0	0	0
**S5-B-CTRL**	0	0	1.26	0	0	0	0	0	0	0	0	0	0	0	0	0
**S5-C-CTRL**	0	0	2.43	0	0	0	0	0	0	0	0	0	0	0	0	0
**S5-A-PPC**	0	0	0	0	0	0	0	0	0	0	0	0	0	0	0	0
**S5-B-PPC**	0	0	0	0	0	0	0	0	0	0	0	0	0	0	0	0
**S5-C-PPC**	0	0	0	0	0	0	0	0	0	0	0	0	0.29	0	0	0
**S5-A-PPI**	0	0	0.47	0	0	0	0	0	0	0	0	0	0	0	0	0
**S5-B-PPI**	0	0	0.68	0	0	0	0	0	0	0	0	0	0.18	0	0	0
**S5-C-PPI**	0	0	0.5	0	0	0	0	0	0	0	0	0	0.15	0	0	0
**S6-A-CTRL**	5.33	0	0	0	0	1.17	0	0	0	0	0	0	0	0	0	0
**S6-B-CTRL**	7.34	0	0	0	0	1.21	0	0	0	0	0	0	0	0	0	0
**S6-C-CTRL**	8.08	0	0	0	0	1.87	0	0	0	0	0	0	0	0	0	0
**S6-A-PPC**	2.21	0	0	0	0	0.41	0	0	0	0	0	0	0.12	0.04	0	0
**S6-B-PPC**	2.37	0	0	0	0	0.51	0	0	0	0	0	0	0.19	0.04	0	0
**S6-C-PPC**	2.75	0	0	0	0	0.64	0	0	0	0	0	0	0.22	0.05	0	0
**S6-A-PPI**	6.39	0	0	0	0	1.41	0	0	0	0	0	0	0.45	0.11	0	0
**S6-B-PPI**	6.53	0	0	0	0	1.51	0	0	0	0	0	0	0.49	0	0	0
**S6-C-PPI**	6.8	0	0	0	0	1.16	0	0	0	0	0	0	0.41	0.14	0	0

**Bacterial species**	Dysosmobacter acutus	Dysosmobacter below species threshold	Dysosmobacter segnis	Dysosmobacter welbionis	Eggerthella below species threshold	Eggerthella lenta	Eisenbergiella below species threshold	Eisenbergiella multihit species	Eisenbergiella porci	Eisenbergiella tayi	Emergencia below species threshold	Emergencia timonensis	Enorma below species threshold	Enterobacter asburiae	Enterobacter below species threshold	Enterobacter bugandensis
**S1-A-CTRL**	0	0	0	0	0	0	0	0	0	0	0	0	0	0	0	0
**S1-B-CTRL**	0	0	0	0	0	0	0	0	0	0	0	0	0	0	0	0
**S1-C-CTRL**	0	0	0	0	0	0	0	0	0	0	0	0	0	0	0	0
**S1-A-PPC**	0	0	0	0	0	0	0	0	0	0	0	0	0	0	0	0
**S1-B-PPC**	0	0	0	0	0	0	0	0	0	0	0	0	0	0	0	0
**S1-C-PPC**	0	0	0	0	0	0	0	0	0	0	0	0	0	0	0	0
**S1-A-PPI**	0	0	0	0	0	0	0	0	0	0	0	0	0	0	0	0
**S1-B-PPI**	0	0	0	0	0	0	0	0	0	0	0	0	0	0	0	0
**S1-C-PPI**	0	0	0	0	0	0	0	0	0	0	0	0	0	0	0	0
**S2-A-CTRL**	0	0	0	0	0	0.01	0	0	0	0	0	0	0	0.08	0.18	0.05
**S2-B-CTRL**	0	0	0	0	0	0	0	0	0	0	0	0	0	0.15	0.3	0.08
**S2-C-CTRL**	0	0	0	0	0	0.02	0.01	0	0	0	0	0	0	0.17	0.34	0.09
**S2-A-PPC**	0	0	0	0	0	0.07	0	0.01	0.01	0	0	0	0	0.2	0.2	0.08
**S2-B-PPC**	0	0.02	0	0	0	0.05	0.01	0.01	0	0	0	0	0	0.17	0.21	0.1
**S2-C-PPC**	0	0	0	0	0	0.02	0	0.01	0	0	0	0	0	0.15	0.21	0.08
**S2-A-PPI**	0	0	0	0	0	0	0.02	0.01	0.01	0	0	0	0	0.4	0.42	0.09
**S2-B-PPI**	0	0	0	0	0	0.02	0.01	0.02	0.01	0	0	0	0	0.32	0.28	0.08
**S2-C-PPI**	0	0.03	0	0	0	0	0.01	0	0.01	0	0	0	0	0.23	0.41	0.14
**S3-A-CTRL**	0	0.19	0	0	0	0	0	0	0	0	0	0	0	0	0	0
**S3-B-CTRL**	0	0	0	0	0	0	0	0	0	0	0	0	0	0.15	0	0
**S3-C-CTRL**	0	0.22	0	0	0	0	0	0	0	0	0	0	0	0	0	0
**S3-A-PPC**	0	0.03	0.02	0	0	0	0	0	0	0	0	0	0	0	0	0
**S3-B-PPC**	0	0.03	0	0	0	0	0	0	0	0	0	0	0	0	0	0
**S3-C-PPC**	0	0.03	0.02	0	0	0	0	0	0	0	0	0	0	0	0	0
**S3-A-PPI**	0	0.14	0	0	0	0	0	0	0	0	0	0	0	0	0	0
**S3-B-PPI**	0	0.13	0	0	0	0	0	0	0	0	0	0	0	0.05	0	0
**S3-C-PPI**	0	0.12	0	0	0	0	0	0	0	0	0	0	0	0	0	0
**S4-A-CTRL**	0	0	0	0	0	0.07	0	0	0	0	0	0	0	0	0.04	0
**S4-B-CTRL**	0	0	0	0	0	0	0	0	0	0	0	0	0	0	0	0
**S4-C-CTRL**	0	0	0	0	0	0	0	0	0	0	0	0	0	0	0	0
**S4-A-PPC**	0	0	0.28	0	0	0	0	0	0	0	0	0	0	0	0.36	0
**S4-B-PPC**	0	0	0	0	0	0	0	0	0	0	0	0	0	0	0.4	0
**S4-C-PPC**	0	0	0.25	0	0	0	0	0	0	0	0	0	0	0	0.29	0
**S4-A-PPI**	0	0	0.05	0	0	0	0	0	0	0	0	0	0	0	0.09	0
**S4-B-PPI**	0	0	0	0	0	0	0	0	0	0	0	0	0	0	0.04	0
**S4-C-PPI**	0	0	0.06	0	0	0	0	0	0	0	0	0	0	0	0	0
**S5-A-CTRL**	0	0	0	0	0	0	0	0	0	0	0	0	0	0	0	0
**S5-B-CTRL**	0	0	0	0	0	0	0	0	0	0	0	0	0	0	0	0
**S5-C-CTRL**	0	0	0	0	0	0	0	0	0	0	0	0	0	0	0	0
**S5-A-PPC**	0	0	0	0	0	0	0	0	0	0	0	0	0	0	0	0
**S5-B-PPC**	0	0	0	0	0	0	0	0	0	0	0	0	0	0	0	0
**S5-C-PPC**	0	0	0	0	0	0	0	0	0	0	0	0	0	0	0	0
**S5-A-PPI**	0	0	0	0	0	0	0	0	0	0	0	0	0	0	0	0
**S5-B-PPI**	0	0	0	0	0	0	0	0	0	0	0	0	0	0	0	0
**S5-C-PPI**	0	0	0	0	0	0	0	0	0	0	0	0	0	0	0	0
**S6-A-CTRL**	0	0.25	0.19	0	0	0	0	0	0	0	0	0	0	0	0.11	0
**S6-B-CTRL**	0	0.26	0.24	0	0	0	0	0	0	0	0	0	0	0	0.12	0
**S6-C-CTRL**	0	0.19	0	0	0	0	0	0	0	0	0	0	0	0	0.16	0
**S6-A-PPC**	0	0.03	0	0	0	0	0	0	0	0	0	0	0	0	0.02	0
**S6-B-PPC**	0	0.04	0	0	0	0	0	0	0	0	0	0	0	0	0.02	0
**S6-C-PPC**	0	0.05	0.05	0	0	0	0	0	0	0	0	0	0	0	0.02	0
**S6-A-PPI**	0	0.13	0.06	0	0	0	0	0	0	0	0	0	0	0	0.07	0
**S6-B-PPI**	0	0.18	0.09	0	0	0	0	0	0	0	0	0	0	0	0	0
**S6-C-PPI**	0	0.11	0.07	0	0	0	0	0	0	0	0	0	0	0	0.04	0

**Bacterial species**	Enterobacter cancerogenus	Enterobacter chengduensis	Enterobacter chuandaensis	Enterobacter cloacae	Enterobacter hormaechei	Enterobacter intestinihominis	Enterobacter kobei	Enterobacter ludwigii	Enterobacter mori	Enterobacter multihit species	Enterobacter pasteurii	Enterobacter quasihormaechei	Enterobacter roggenkampii	Enterobacter sichuanensis	Enterobacter soli	Enterocloster asparagiformis
**S1-A-CTRL**	0	0	0	0	0	0	0	0	0	0.49	0	0	0	0	0	0
**S1-B-CTRL**	0	0	0	0	0	0	0	0	0	0.65	0	0	0	0	0	0
**S1-C-CTRL**	0	0	0	0	0	0	0	0	0	0.37	0	0	0	0	0	0
**S1-A-PPC**	0	0	0	0	0	0	0	0	0	0.32	0	0	0	0	0	0
**S1-B-PPC**	0	0	0	0	0	0	0	0	0	0.25	0	0	0	0	0	0
**S1-C-PPC**	0	0	0	0	0	0	0	0	0	0.33	0	0	0	0	0	0
**S1-A-PPI**	0	0	0	0	0	0	0	0	0	0.42	0	0	0	0	0	0
**S1-B-PPI**	0	0	0	0	0	0	0	0	0	0.42	0	0	0	0	0	0
**S1-C-PPI**	0	0	0	0	0	0	0	0	0	0.14	0	0	0	0	0	0
**S2-A-CTRL**	0.02	0.01	0	0.05	2.16	0.56	9.56	0.07	0.03	4.31	0	0.03	0.05	0.01	0.18	0
**S2-B-CTRL**	0.02	0.01	0	0.09	3.39	0.89	14.3	0.09	0.02	6.45	0.01	0.03	0.09	0.02	0.26	0
**S2-C-CTRL**	0.02	0	0	0.1	3.17	0.74	17	0.13	0.03	6.31	0.01	0.03	0.08	0.03	0.35	0
**S2-A-PPC**	0.02	0	0	0.06	2.89	0.81	7.32	0.07	0.02	5.65	0	0.03	0.07	0	0.14	0
**S2-B-PPC**	0.02	0	0	0.06	2.85	0.81	7.78	0.08	0.02	5.64	0	0.02	0.09	0	0.16	0
**S2-C-PPC**	0.03	0	0	0.08	2.75	0.76	7.95	0.07	0.01	5.31	0	0.03	0.1	0.02	0.15	0
**S2-A-PPI**	0.04	0.02	0	0.12	2.81	0.6	25.15	0.15	0.05	6.62	0	0.03	0.11	0.04	0.5	0
**S2-B-PPI**	0.04	0.01	0	0.09	3.82	1.04	13.09	0.11	0.05	7.46	0.02	0.04	0.13	0.03	0.26	0
**S2-C-PPI**	0.03	0.01	0.02	0.13	4.18	1.12	20.61	0.15	0.04	8.79	0	0.04	0.15	0.04	0.41	0
**S3-A-CTRL**	0	0	0	0	0	0	0	0	0	1.86	0	0	0	0	0	0
**S3-B-CTRL**	0	0	0	0	0	0.18	0	0	0	2.71	0	0	0	0	0	0
**S3-C-CTRL**	0	0	0	0.13	0	0	0	0	0	2.72	0	0	0	0	0	0
**S3-A-PPC**	0	0	0	0	0	0	0	0	0	0.14	0	0	0	0	0	0
**S3-B-PPC**	0	0	0	0	0	0	0	0	0	0.14	0	0	0	0	0	0
**S3-C-PPC**	0	0	0	0	0	0	0	0	0	0.15	0	0	0	0	0	0
**S3-A-PPI**	0	0	0	0	0	0	0	0	0	1.34	0	0	0	0	0	0
**S3-B-PPI**	0	0	0	0	0	0	0	0	0	0.87	0	0	0	0	0	0
**S3-C-PPI**	0	0	0	0	0	0	0	0	0	1.07	0	0	0	0	0	0
**S4-A-CTRL**	0	0	0	0	0	0	0	0	0	0.11	0	0	0	0	0	0
**S4-B-CTRL**	0	0	0	0	0	0	0	0	0	0.09	0	0	0	0	0	0
**S4-C-CTRL**	0	0	0	0	0	0	0	0	0	0.1	0	0	0	0	0	0
**S4-A-PPC**	0	0	0	0	0	0	0	0	0	0.98	0	0	0	0	0	0
**S4-B-PPC**	0	0	0	0	0	0	0	0	0	1.23	0	0	0	0	0	0
**S4-C-PPC**	0	0	0	0	0	0	0	0	0	0.71	0	0	0	0	0	0
**S4-A-PPI**	0	0	0	0	0	0	0	0	0	0.21	0	0	0	0	0	0
**S4-B-PPI**	0	0	0	0	0	0	0	0	0	0.12	0	0	0	0	0	0
**S4-C-PPI**	0	0	0	0	0	0	0	0	0	0.11	0	0	0	0	0	0
**S5-A-CTRL**	0	0	0	0	0	0	0	0	0	0.31	0	0	0	0	0	0
**S5-B-CTRL**	0	0	0	0	0	0	0	0	0	0.55	0	0	0	0	0	0
**S5-C-CTRL**	0	0	0	0	0	0	0	0	0	0.75	0	0	0	0	0	0
**S5-A-PPC**	0	0	0	0	0	0	0	0	0	0.47	0	0	0	0	0	0
**S5-B-PPC**	0	0	0	0	0	0	0	0	0	0.3	0	0	0	0	0	0
**S5-C-PPC**	0	0	0	0	0	0	0	0	0	0.25	0	0	0	0	0	0
**S5-A-PPI**	0	0	0	0	0	0	0	0	0	0.28	0	0	0	0	0	0
**S5-B-PPI**	0	0	0	0	0	0	0	0	0	0.25	0	0	0	0	0	0
**S5-C-PPI**	0	0	0	0	0	0	0	0	0	0.4	0	0	0	0	0	0
**S6-A-CTRL**	0	0	0	0	0	0	0	0	0	0	0	0	0	0	0	0
**S6-B-CTRL**	0	0	0	0	0	0	0	0	0	0	0	0	0	0	0	0
**S6-C-CTRL**	0	0	0	0	0	0	0	0	0	0	0	0	0	0	0	0
**S6-A-PPC**	0	0	0	0	0	0	0	0	0	0	0	0	0	0	0	0
**S6-B-PPC**	0	0	0	0	0	0	0	0	0	0	0	0	0	0	0	0
**S6-C-PPC**	0	0	0	0	0	0	0	0	0	0	0	0	0	0	0	0
**S6-A-PPI**	0	0	0	0	0	0	0	0	0	0.04	0	0	0	0	0	0
**S6-B-PPI**	0	0	0	0	0	0	0	0	0	0	0	0	0	0	0	0
**S6-C-PPI**	0	0	0	0	0	0	0	0	0	0	0	0	0	0	0	0

**Bacterial species**	Enterocloster below species threshold	Enterocloster bolteae	Enterocloster citroniae	Enterocloster lavalensis	Enterococcus below species threshold	Enterococcus casseliflavus	Enterococcus durans	Enterococcus faecalis	Enterococcus faecium	Enterococcus hirae	Enterococcus italicus	Enterococcus lactis	Enterococcus multihit species	Enterococcus villorum	Escherichia albertii	Escherichia below species threshold
**S1-A-CTRL**	0	0	0	0	0	0	0	0	0	0	0	0	0	0	0	2.54
**S1-B-CTRL**	0	0	0	0	0	0	0	0	0	0	0	0	0	0	0.33	3.24
**S1-C-CTRL**	0.17	0.17	0	0	0	0	0	0	0	0	0	0	0	0	0.24	2.33
**S1-A-PPC**	0.1	0	0	0	0	0	0	0	0	0	0	0	0	0	0.12	1.76
**S1-B-PPC**	0.09	0	0	0	0	0	0	0	0	0	0	0	0	0	0.1	1.87
**S1-C-PPC**	0.09	0	0	0	0	0	0	0	0	0	0	0	0	0	0.08	1.58
**S1-A-PPI**	0	0	0	0	0	0	0	0	0	0	0	0	0	0	0.15	2.36
**S1-B-PPI**	0	0	0	0	0	0	0	0	0	0	0	0	0	0	0.2	2.53
**S1-C-PPI**	0	0	0	0	0	0	0	0	0	0	0	0	0	0	0.13	1.17
**S2-A-CTRL**	0	0	0	0	0	0	0	0	0	0	0	0	0	0	0.02	0.23
**S2-B-CTRL**	0	0	0	0	0	0	0	0	0	0	0	0	0	0	0.05	0.36
**S2-C-CTRL**	0	0	0	0	0	0	0	0	0	0	0	0	0	0	0.02	0.24
**S2-A-PPC**	0.01	0.01	0	0	0	0	0	0	0	0	0	0	0	0	0.02	0.16
**S2-B-PPC**	0	0	0	0	0	0	0	0	0	0	0	0	0	0	0.03	0.2
**S2-C-PPC**	0	0.01	0	0	0	0	0	0	0	0	0	0	0	0	0	0.12
**S2-A-PPI**	0.02	0.01	0	0	0	0	0	0	0	0	0	0	0	0	0.04	0.26
**S2-B-PPI**	0.01	0.01	0	0	0	0	0	0	0	0	0	0	0	0	0	0.15
**S2-C-PPI**	0.01	0	0	0	0	0	0	0	0.02	0	0	0	0	0	0	0.15
**S3-A-CTRL**	0	0	0	0	0.55	0	0.2	0	10.29	0	0	0.37	0.03	0	0.41	2.9
**S3-B-CTRL**	0	0	0	0	0.72	0	0.44	0	10.34	0	0	0.29	0.02	0	0.42	4.25
**S3-C-CTRL**	0	0	0	0	0.83	0	0.55	0	11.93	0	0	0	0.02	0	0.49	3.48
**S3-A-PPC**	0	0	0	0	0.63	0.07	0.17	0	6.23	0.09	0	0.24	0.04	0	0.03	0.24
**S3-B-PPC**	0	0	0	0	0.67	0.07	0.15	0.03	6.62	0.1	0	0.31	0.04	0	0.04	0.3
**S3-C-PPC**	0	0	0	0	0.53	0.07	0.13	0	6.12	0.09	0	0.28	0.04	0	0.04	0.25
**S3-A-PPI**	0	0	0	0	0.81	0	1.07	0	20.44	0	0	0.51	0.07	0	0.24	2.94
**S3-B-PPI**	0	0	0	0	1.32	0.09	1.64	0	24.88	0.23	0	0.63	0.15	0	0.13	2.55
**S3-C-PPI**	0	0	0	0	1.02	0	1.37	0	30.27	0.21	0	0.72	0.16	0	0.15	1.78
**S4-A-CTRL**	0	0	0	0	0	0	0	0	0	0	0	0	0	0	0.11	0.49
**S4-B-CTRL**	0	0	0	0	0	0	0	0	0	0	0	0	0	0	0.08	0.44
**S4-C-CTRL**	0	0	0	0	0	0	0	0	0	0	0	0	0	0	0.09	0.42
**S4-A-PPC**	0	0	0	0	0	0	0	0	1.9	0	0	0	0	0	0.93	4.04
**S4-B-PPC**	0	0	0	0	0.52	0	0	0	3.67	0	0	0	0	0	0.65	3.59
**S4-C-PPC**	0	0	0	0	0	0	0	0	0.62	0	0	0	0	0	0.74	3.28
**S4-A-PPI**	0	0	0	0	0.07	0	0	0	0.07	0	0	0	0	0	0.15	0.69
**S4-B-PPI**	0	0	0	0	0	0	0	0	0	0	0	0	0	0	0.09	0.46
**S4-C-PPI**	0	0	0	0	0	0	0	0	0	0	0	0	0	0	0.1	0.48
**S5-A-CTRL**	0	0	0	0	0	0	0	0	0	0.36	0	0	0	0	0.64	3.22
**S5-B-CTRL**	0	0	0	0	0	0	0	0	0	0.59	0	0	0	0	0.62	3.94
**S5-C-CTRL**	0	0	0	0	0	0	0	0	0	0	0	0	0	0	0.91	3.66
**S5-A-PPC**	0	0	0	0	0	0	0	0	0	0.38	0	0	0	0	0.55	2.83
**S5-B-PPC**	0	0	0	0	0	0	0	0	0	0.33	0	0	0	0	0.86	2.92
**S5-C-PPC**	0	0	0	0	0	0	0	0	0	0	0	0	0	0	0.75	2.73
**S5-A-PPI**	0	0	0	0	0	0	0	0	0	0	0	0	0	0	0.47	2.49
**S5-B-PPI**	0	0	0	0	0	0	0	0	0	0	0	0	0	0	0.4	1.78
**S5-C-PPI**	0	0	0	0	0	0	0	0	0	0.25	0	0	0	0	0.42	1.29
**S6-A-CTRL**	0	0	0	0	1.38	0	1.31	33.7	0.94	0	0	0.5	0	0	0.56	12.48
**S6-B-CTRL**	0	0	0	0	2.2	0	2.28	40.65	0.88	0	0	0.69	0	0	0.61	7.63
**S6-C-CTRL**	0	0	0	0	1.81	0	1.95	40.31	1.18	0	0	0.62	0.01	0	0.45	7.02
**S6-A-PPC**	0	0	0	0	1.28	0.06	0.19	13.44	10.93	0.07	0	2.65	0.36	0.03	0.02	0.94
**S6-B-PPC**	0	0	0	0	1.36	0.08	0.22	13.78	11.34	0.17	0	2.72	0.38	0	0.04	1.11
**S6-C-PPC**	0	0	0	0	1.67	0.09	0.26	14.13	12.11	0.1	0.07	2.73	0.39	0.06	0.03	1.14
**S6-A-PPI**	0	0	0	0	1.3	0	0.29	31.23	7.99	0.11	0	1.95	0.22	0	0.12	3.13
**S6-B-PPI**	0	0	0	0	1.36	0	0.37	37.69	9.45	0.27	0	2.24	0.24	0	0.18	2.79
**S6-C-PPI**	0	0	0	0	1.67	0.07	0.26	35.53	10.07	0.1	0	2.18	0.27	0	0.12	2.84

**Bacterial species**	Escherichia coli	Escherichia fergusonii	Escherichia multihit species	Eshraghiella below species threshold	Eshraghiella crossota	Eubacterium album	Eubacterium below species threshold	Eubacterium ramulus	Eubacterium segne	Eubacterium ventriosum	Evtepia below species threshold	Evtepia gabavorous	Faecalibacillus below species threshold	Faecalibacillus intestinalis	Faecalibacterium below species threshold	Faecalibacterium duncaniae
**S1-A-CTRL**	0.58	0.23	0.11	0	0	0	0	0	0	0.32	0	0	0	0	0	0
**S1-B-CTRL**	0.59	0.24	0.1	0	0	0	0	0	0	0	0	0	0	0	0	0
**S1-C-CTRL**	0.56	0.21	0.1	0	0	0	0	0	0	0.48	0	0	0	0	0	0
**S1-A-PPC**	0.5	0.13	0.1	0	0	0	0	0	0	0.69	0	0	0	0	0	0.14
**S1-B-PPC**	0.5	0.12	0.09	0	0	0	0	0	0	1.18	0	0	0	0	0.16	0
**S1-C-PPC**	0.49	0	0.09	0	0	0	0	0	0	0.73	0	0	0	0	0	0.19
**S1-A-PPI**	0.56	0	0.09	0	0	0	0	0	0	0.27	0	0	0	0	0	0
**S1-B-PPI**	0.57	0.14	0.1	0	0	0	0	0	0	0.62	0	0	0	0	0	0
**S1-C-PPI**	0.48	0.12	0.09	0	0	0	0	0	0	0.6	0	0	0	0	0	0
**S2-A-CTRL**	0.01	0.03	0	0	0	0	0	0	0	0.03	0	0	0	0	0.46	0.17
**S2-B-CTRL**	0.02	0.04	0	0	0	0	0	0	0	0.03	0	0	0	0	0.34	0.12
**S2-C-CTRL**	0.01	0.03	0	0	0	0	0	0	0	0.05	0	0	0	0	0.35	0.14
**S2-A-PPC**	0.01	0.01	0	0	0	0	0.07	0	0	0.07	0	0	0	0	0.43	0.62
**S2-B-PPC**	0.01	0.02	0	0	0	0	0.06	0	0	0.06	0	0	0	0	0.42	0.6
**S2-C-PPC**	0	0	0	0	0	0	0.06	0	0	0.07	0	0	0	0	0.35	0.53
**S2-A-PPI**	0.01	0.03	0	0	0	0	0.06	0	0	0	0	0	0	0	0.33	0.75
**S2-B-PPI**	0.01	0.02	0	0	0	0	0.06	0	0	0.06	0	0	0	0	0.27	0.72
**S2-C-PPI**	0	0.01	0	0	0	0	0.06	0	0	0.04	0	0	0	0	0.29	0.64
**S3-A-CTRL**	0.3	4.75	0.1	0	0	0	0	0	0	0	0	0	0	0	0	0
**S3-B-CTRL**	0.36	9.03	0.11	0	0	0	0	0	0	0	0	0	0	0	0.33	0
**S3-C-CTRL**	0.36	6.04	0.11	0	0	0	0	0	0	0	0	0	0	0	0	0
**S3-A-PPC**	0.05	0.29	0.01	0	0	0	0.03	0	0	0	0	0	0	0	0.09	0.06
**S3-B-PPC**	0.06	0.33	0.01	0	0	0	0.02	0	0	0	0	0	0	0	0.07	0.05
**S3-C-PPC**	0.06	0.35	0.01	0	0	0	0	0	0	0	0	0	0	0	0.07	0.04
**S3-A-PPI**	0.33	4.37	0.07	0	0	0	0	0	0	0	0	0	0	0	0.2	0.21
**S3-B-PPI**	0.16	4.88	0.05	0	0	0	0	0	0	0	0	0	0	0	0.2	0.16
**S3-C-PPI**	0.19	3.7	0.06	0	0	0	0	0	0	0	0	0	0	0	0.26	0.2
**S4-A-CTRL**	0.31	0.08	0.06	0	0	0	0	0	0	0	0	0	0	0	0.08	0
**S4-B-CTRL**	0.3	0.1	0.06	0	0	0	0	0	0	0	0	0	0	0	0.09	0
**S4-C-CTRL**	0.29	0.09	0.06	0	0	0	0	0	0	0	0	0	0	0	0.09	0
**S4-A-PPC**	0.58	0.49	0.12	0	0	0	0.3	0	0	0	0	0	0	0	1.25	1.23
**S4-B-PPC**	0.57	0.53	0.12	0	0	0	0	0	0	0	0	0	0	0	1.44	1.1
**S4-C-PPC**	0.56	0.72	0.11	0	0	0	0	0	0	0	0	0	0	0	0.88	0.74
**S4-A-PPI**	0.33	0.12	0.07	0	0	0	0	0	0	0	0	0	0	0	0.28	0.24
**S4-B-PPI**	0.31	0.09	0.06	0	0	0	0	0	0	0	0	0	0	0	0.19	0.1
**S4-C-PPI**	0.31	0.11	0.06	0	0	0	0	0	0	0	0	0	0	0	0.22	0.19
**S5-A-CTRL**	0.59	1.36	0.11	0	0	0	0	0	0	0	0	0	0	0	0	0
**S5-B-CTRL**	0.6	1.11	0.11	0	0	0	0	0	0	0	0	0	0	0	0	0
**S5-C-CTRL**	0.61	1.38	0.11	0	0	0	0	0	0	0	0	0	0	0	0	0
**S5-A-PPC**	0.58	1.32	0.11	0	0	0	0	0	0	0	0	0	0	0	0	0
**S5-B-PPC**	0.59	1.36	0.12	0	0	0	0	0	0	0	0	0	0	0	0	0
**S5-C-PPC**	0.58	1.05	0.12	0	0	0	0	0	0	0	0	0	0	0	0.27	0
**S5-A-PPI**	0.56	0.62	0.1	0	0	0	0	0	0	0	0	0	0	0	0	0
**S5-B-PPI**	0.55	0.77	0.1	0	0	0	0	0	0	0	0	0	0	0	0	0
**S5-C-PPI**	0.53	0.46	0.1	0	0	0	0	0	0	0	0	0	0	0	0	0
**S6-A-CTRL**	0.45	2.32	0.09	0	0	0	0	0	0	0	0	0	0	0	0.43	0
**S6-B-CTRL**	0.48	1.88	0.08	0	0	0	0	0	0	0	0	0	0	0	0.44	0
**S6-C-CTRL**	0.45	1.52	0.09	0	0	0	0	0	0	0	0	0	0	0	0.44	0
**S6-A-PPC**	0.04	0.14	0.01	0	0	0	0	0	0	0	0	0	0	0	0.12	0.03
**S6-B-PPC**	0.04	0.1	0.01	0	0	0	0	0	0	0.04	0	0	0	0	0.13	0
**S6-C-PPC**	0.04	0.15	0.01	0	0	0	0	0	0	0	0	0	0	0	0.16	0.04
**S6-A-PPI**	0.17	0.45	0.03	0	0	0	0	0	0.07	0.13	0	0	0	0	0.26	0.12
**S6-B-PPI**	0.19	0.55	0.03	0	0	0	0	0	0	0	0	0	0	0	0.43	0
**S6-C-PPI**	0.16	0.55	0.03	0	0	0	0	0	0.06	0.14	0	0	0	0	0.27	0.1

Bacterial species	Faecalibacterium faecis	Faecalibacterium hattorii	Faecalibacterium intestinale	Faecalibacterium multihit species	Faecalibacterium prausnitzii	Faecalibacterium taiwanense	Faecalibacterium tardum	Faecalibacterium wellingii	Faecalicatena below species threshold	Faecalicatena contorta	Faecalicoccus below species threshold	Faecalicoccus pleomorphus	Faecalispora below species threshold	Faecousia below species threshold	Faecousia intestinalis	Feifania below species threshold
**S1-A-CTRL**	0	0	0	0	0.36	0	0	0	0	0	0	0	0	0	0	0
**S1-B-CTRL**	0	0	0	0	0.36	0	0	0	0	0	0	0	0	0	0	0
**S1-C-CTRL**	0	0	0	0	0.33	0	0	0	0	0	0	0	0	0	0	0
**S1-A-PPC**	0	0	0	0	0.21	0	0	0	0	0	0	0	0	0	0	0
**S1-B-PPC**	0	0	0	0	0.3	0	0	0	0	0	0	0	0	0	0	0
**S1-C-PPC**	0	0	0	0.2	0.28	0.13	0	0	0	0	0	0	0	0	0	0
**S1-A-PPI**	0	0	0	0	0	0	0	0	0	0	0	0	0	0	0	0
**S1-B-PPI**	0	0	0	0	0.27	0	0	0	0	0	0	0	0	0	0	0
**S1-C-PPI**	0	0	0	0.12	0.2	0	0	0	0	0	0	0	0	0	0	0
**S2-A-CTRL**	0.12	0.02	0.01	0.9	0.34	0.61	0	1.02	0	0	0	0	0	0.02	0	0
**S2-B-CTRL**	0.12	0	0	0.78	0.34	0.59	0	0.86	0	0	0	0	0	0.02	0	0
**S2-C-CTRL**	0.13	0.02	0	0.74	0.3	0.51	0	0.86	0	0	0	0	0	0.02	0	0
**S2-A-PPC**	0.14	0	0	0.84	0.39	0.53	0	1.05	0.01	0	0	0	0	0.05	0	0
**S2-B-PPC**	0.13	0.02	0	0.87	0.37	0.58	0	0.96	0	0	0	0	0	0.03	0	0
**S2-C-PPC**	0.12	0	0	0.66	0.27	0.42	0	0.9	0	0	0	0	0	0	0	0
**S2-A-PPI**	0.11	0	0	0.8	0.36	0.47	0	0.9	0	0	0	0	0	0.05	0	0
**S2-B-PPI**	0.15	0	0	0.69	0.37	0.51	0	1.04	0	0	0	0	0	0.03	0	0
**S2-C-PPI**	0.12	0	0	0.84	0.29	0.47	0	0.92	0	0	0	0	0	0.04	0	0
**S3-A-CTRL**	0	0	0	0	0	0	0	0	0	0	0	0	0	0	0	0
**S3-B-CTRL**	0	0	0	0.29	0	0	0	0	0	0	0	0	0	0	0	0
**S3-C-CTRL**	0	0	0	0.22	0.24	0.3	0	0	0	0	0	0	0	0	0	0
**S3-A-PPC**	0	0	0	0.07	0.03	0.06	0	0	0	0	0	0.08	0	0.02	0	0
**S3-B-PPC**	0.02	0	0	0.09	0.03	0.03	0	0	0	0	0	0.05	0	0	0.03	0
**S3-C-PPC**	0	0	0	0.08	0.03	0.03	0	0	0	0	0	0.07	0	0	0.03	0
**S3-A-PPI**	0	0	0	0.24	0	0.17	0	0	0	0	0	0	0	0.14	0	0
**S3-B-PPI**	0	0	0	0.13	0	0.13	0	0	0	0	0	0.11	0	0	0	0
**S3-C-PPI**	0	0	0	0.18	0	0	0	0	0	0	0	0	0	0	0	0
**S4-A-CTRL**	0	0	0	0	0	0	0	0	0	0	0	0	0	0	0	0
**S4-B-CTRL**	0	0	0	0	0	0	0	0	0	0	0	0	0	0	0	0
**S4-C-CTRL**	0	0	0	0	0	0	0	0	0	0	0	0	0	0	0	0
**S4-A-PPC**	0	0	0	0.61	0	0	0	0	0	0	0	0	0	0.39	0	0
**S4-B-PPC**	0	0	0	0.44	0.33	0	0	0	0	0	0	0	0	0	0	0
**S4-C-PPC**	0	0	0	0	0	0	0	0	0	0	0	0	0	0.34	0	0
**S4-A-PPI**	0	0	0	0.1	0	0	0	0	0	0	0	0	0	0.09	0	0
**S4-B-PPI**	0	0	0	0.09	0	0	0	0	0	0	0	0	0	0.09	0	0
**S4-C-PPI**	0	0	0	0	0	0	0	0	0	0	0	0	0	0	0	0
**S5-A-CTRL**	0	0	0	0	0	0	0	0	0	0.22	0	0	0	0	0	0
**S5-B-CTRL**	0	0	0	0	0	0	0	0	0	0.26	0	0	0	0	0	0
**S5-C-CTRL**	0	0	0	0	0	0	0	0	0	0	0	0	0	0	0	0
**S5-A-PPC**	0	0	0	0	0	0	0	0	0	0	0	0	0	0	0	0
**S5-B-PPC**	0	0	0	0	0	0	0	0	0	0	0	0	0	0	0	0
**S5-C-PPC**	0	0	0	0	0	0	0	0	0	0.17	0	0	0	0	0	0
**S5-A-PPI**	0	0	0	0	0	0	0	0	0	0	0	0	0	0	0	0
**S5-B-PPI**	0	0	0	0	0	0	0	0	0	0	0	0	0	0	0	0
**S5-C-PPI**	0	0	0	0	0	0	0	0	0	0	0	0	0	0	0	0
**S6-A-CTRL**	0	0	0	0.27	0	0.2	0	0.36	0	0	0	0	0	0.24	0	0
**S6-B-CTRL**	0	0	0	0.27	0.2	0.26	0	0	0	0	0	0	0	0	0	0
**S6-C-CTRL**	0	0	0	0.31	0.17	0	0	0	0	0	0	0	0	0	0	0
**S6-A-PPC**	0	0	0	0.07	0.07	0.04	0	0.05	0	0	0	0	0	0.04	0	0
**S6-B-PPC**	0	0	0	0.08	0.09	0.08	0	0.05	0	0	0	0	0	0	0	0
**S6-C-PPC**	0.04	0	0	0.06	0.07	0.06	0	0.05	0	0	0	0	0	0.05	0	0
**S6-A-PPI**	0	0	0	0.13	0.17	0.11	0	0.11	0	0	0	0	0	0.11	0	0
**S6-B-PPI**	0	0	0	0.18	0.13	0.19	0	0.11	0	0	0	0	0	0.13	0	0
**S6-C-PPI**	0	0	0	0.14	0.19	0.18	0	0.1	0	0	0	0	0	0.08	0	0

**Bacterial species**	Flavobacterium below species threshold	Flavonifractor below species threshold	Flavonifractor plautii	Flintibacter below species threshold	Flintibacter faecis	Flintibacter hominis	Franconibacter helveticus	Frisingicoccus below species threshold	Frisingicoccus caecimuris	Fumia below species threshold	Fusicatenibacter below species threshold	Fusicatenibacter faecihominis	Fusobacterium below species threshold	Fusobacterium hominis	Fusobacterium necrogenes	Gabonibacter below species threshold
**S1-A-CTRL**	0	0	0.42	0	0	0	0	0	0	0	0	0	0	0	0	0
**S1-B-CTRL**	0	0	0	0	0	0	0	0	0	0	0	0	0	0	0	0
**S1-C-CTRL**	0	0	0.27	0	0	0	0	0	0	0	0	0	0	0	0	0
**S1-A-PPC**	0	0	0.11	0	0	0	0	0	0	0	0	0	3.2	0	1.7	0
**S1-B-PPC**	0	0.11	0	0	0	0	0	0	0	0	0	0	2.06	0	1.2	0
**S1-C-PPC**	0	0.1	0.21	0	0	0	0	0	0	0	0	0	1.85	0	1.24	0
**S1-A-PPI**	0	0	0.17	0	0	0	0	0	0	0	0	0	18.2	0	10.85	0
**S1-B-PPI**	0	0	0.22	0	0	0	0	0	0	0	0	0	3.65	0	3.33	0
**S1-C-PPI**	0	0.08	0.14	0	0	0	0	0	0	0	0	0	21.32	0.31	12.57	0
**S2-A-CTRL**	0	0.02	0.05	0	0	0	0	0	0	0	0	0	0	0	0	0
**S2-B-CTRL**	0	0	0.05	0	0	0	0	0	0	0	0	0	0	0	0	0
**S2-C-CTRL**	0	0.02	0.04	0	0	0	0	0	0	0	0	0	0	0	0	0
**S2-A-PPC**	0	0.02	0.06	0	0	0	0	0	0	0	0	0	0	0	0	0
**S2-B-PPC**	0	0.03	0.07	0.02	0	0	0	0	0	0	0	0	0	0	0	0
**S2-C-PPC**	0	0.03	0.05	0.02	0	0	0	0	0	0	0	0	0	0	0	0
**S2-A-PPI**	0	0.03	0.05	0	0	0	0	0	0	0	0	0	0	0	0	0
**S2-B-PPI**	0	0.02	0.05	0.02	0	0	0	0	0	0	0	0	0	0	0	0
**S2-C-PPI**	0	0.02	0.07	0	0	0	0	0	0	0	0	0	0	0	0	0
**S3-A-CTRL**	0	0	0	0	0	0	0	0	0	0	0	0	0	0	0	0
**S3-B-CTRL**	0	0.2	0.18	0	0	0	0	0	0	0	0	0	0	0	0	0
**S3-C-CTRL**	0	0	0.16	0	0	0	0	0	0	0	0	0	0	0	0	0
**S3-A-PPC**	0	0.02	0.02	0	0	0	0	0	0	0	0	0	0	0	0	0
**S3-B-PPC**	0	0.02	0.02	0	0	0	0	0	0	0	0	0	0	0	0	0
**S3-C-PPC**	0	0.02	0	0.02	0	0	0	0	0	0	0	0	0	0	0	0
**S3-A-PPI**	0	0.1	0	0	0	0	0	0	0	0	0	0	0	0	0	0
**S3-B-PPI**	0	0.1	0	0	0	0	0	0	0	0	0	0	0	0	0	0
**S3-C-PPI**	0	0.09	0	0.1	0	0	0	0	0	0	0	0	0	0	0	0
**S4-A-CTRL**	0	0	0	0	0	0	0	0	0	0	0	0	0	0	0	0
**S4-B-CTRL**	0	0	0	0	0	0	0	0	0	0	0	0	0	0	0	0
**S4-C-CTRL**	0	0.04	0	0	0	0	0	0	0	0	0	0	0	0	0	0
**S4-A-PPC**	0	0	0.29	0	0	0	0	0	0	0	0	0	0	0	0	0
**S4-B-PPC**	0	0.33	0.22	0	0	0	0	0	0	0	0	0	0	0	0	0
**S4-C-PPC**	0	0	0.25	0	0	0	0	0	0	0	0	0	0	0	0	0
**S4-A-PPI**	0	0	0.05	0	0	0	0	0	0	0	0	0	0	0	0	0
**S4-B-PPI**	0	0.05	0.1	0	0	0	0	0	0	0	0	0	0	0	0	0
**S4-C-PPI**	0	0	0.08	0.05	0	0	0	0	0	0	0	0	0	0	0	0
**S5-A-CTRL**	0	0	0.41	0	0	0	0	0	0	0	0	0	0	0	0	0
**S5-B-CTRL**	0	0	0.32	0	0	0	0	0	0	0	0	0	0	0	0	0
**S5-C-CTRL**	0	0	0	0	0	0	0	0	0	0	0	0	0	0	0	0
**S5-A-PPC**	0	0	0.27	0	0	0	0	0	0	0	0	0	0	0	0	0
**S5-B-PPC**	0	0	0	0	0	0	0	0	0	0	0	0	0	0	0	0
**S5-C-PPC**	0	0	0.27	0	0	0	0	0	0	0	0	0	0	0	0	0
**S5-A-PPI**	0	0	0.23	0	0	0	0	0	0	0	0	0	0	0	0	0
**S5-B-PPI**	0	0	0.17	0	0	0	0	0	0	0	0	0	0	0	0	0
**S5-C-PPI**	0	0	0.12	0	0	0	0	0	0	0	0	0	0	0	0	0
**S6-A-CTRL**	0	0	0	0	0	0	0.28	0	0	0	0	0	0	0	0	0
**S6-B-CTRL**	0	0	0	0	0	0	0.28	0	0	0	0	0	0	0	0	0
**S6-C-CTRL**	0	0	0.13	0	0	0	0.18	0	0	0	0	0	0	0	0	0
**S6-A-PPC**	0	0	0.02	0	0	0	0	0	0	0	0	0	0	0	0	0
**S6-B-PPC**	0	0	0.02	0.03	0	0	0	0	0	0	0	0	0	0	0	0
**S6-C-PPC**	0	0	0	0	0	0	0	0	0	0	0	0	0	0	0	0
**S6-A-PPI**	0	0	0	0	0	0	0.04	0	0	0	0	0	0	0	0	0
**S6-B-PPI**	0	0.06	0	0.07	0	0	0.07	0	0	0	0	0	0	0	0	0
**S6-C-PPI**	0	0.07	0.04	0	0	0	0.04	0	0	0	0	0	0	0	0	0

**Bacterial species**	Gabonibacter massiliensis	Gallalistipes below species threshold	Gallintestinimicrobium below species threshold	Gallintestinimicrobium propionicum	Gehongia below species threshold	Gemmiger below species threshold	Gemmiger formicilis	Guopingia below species threshold	Haemophilus parainfluenzae	Hafnia alvei	Hafnia paralvei	Hallella absiana	Hallella below species threshold	Hallella faecis	Hallella mizrahii	Hallella multihit species
**S1-A-CTRL**	0	0	0	0	0	0	0	0	0	0	0	0	0	0	0	0
**S1-B-CTRL**	0	0	0	0	0	0	0	0	0	0	0	0	0	0	0	0
**S1-C-CTRL**	0	0	0	0	0	0	0.37	0	0	0	0	0	0	0	0	0
**S1-A-PPC**	0	0	0	0	0	0	0.18	0	0	0	0	0	0	0	0	0
**S1-B-PPC**	0	0	0	0	0	0	0.22	0	0	0	0	0	0	0	0	0
**S1-C-PPC**	0	0	0	0	0	0	0.16	0	0	0	0	0	0	0	0	0
**S1-A-PPI**	0	0	0	0	0	0	0	0	0	0	0	0	0	0	0	0
**S1-B-PPI**	0	0	0	0	0	0	0.31	0	0	0	0	0	0	0	0	0
**S1-C-PPI**	0	0	0	0	0	0	0.16	0	0	0	0	0	0	0	0	0
**S2-A-CTRL**	0	0	0	0	0	0.04	0.03	0	0	0	0.09	0	0	0	0	0
**S2-B-CTRL**	0	0	0	0	0	0.04	0	0	0	0	0	0	0	0	0	0
**S2-C-CTRL**	0	0	0	0	0	0.04	0.03	0	0	0	0.05	0	0	0	0	0
**S2-A-PPC**	0	0	0	0	0	0.07	0.04	0	0.04	0	0	0	0	0	0	0
**S2-B-PPC**	0	0	0	0	0	0.06	0.02	0	0	0.02	0.06	0	0	0	0	0
**S2-C-PPC**	0	0	0	0	0	0.05	0.02	0	0	0.01	0.02	0	0	0	0	0
**S2-A-PPI**	0	0	0	0	0	0.04	0	0	0	0	0.03	0	0	0	0	0
**S2-B-PPI**	0	0	0	0	0	0.06	0.03	0	0	0.01	0.11	0	0	0	0	0
**S2-C-PPI**	0	0	0	0	0	0.07	0.03	0	0	0	0.04	0	0	0	0	0
**S3-A-CTRL**	0	0	0	0	0	0	0	0	0	0	0	0	0	0	0	0
**S3-B-CTRL**	0	0	0	0	0	0	0	0	0	0	0	0	0	0	0	0
**S3-C-CTRL**	0	0	0	0	0	0	0	0	0	0	0	0	0	0	0	0
**S3-A-PPC**	0	0	0	0	0	0.03	0.1	0	0	0	0	0	0.02	0	0	0
**S3-B-PPC**	0	0	0	0	0	0.05	0.07	0	0	0	0	0	0.03	0	0	0
**S3-C-PPC**	0	0	0	0	0	0.04	0.07	0	0	0	0	0	0.02	0	0	0
**S3-A-PPI**	0	0	0	0	0	0.14	0.11	0	0	0	0	0	0	0	0	0
**S3-B-PPI**	0	0	0	0	0	0.11	0.15	0	0	0	0	0	0	0	0	0
**S3-C-PPI**	0	0	0	0	0	0.13	0.13	0	0	0	0	0	0	0	0	0
**S4-A-CTRL**	0	0	0	0	0	0	0	0	0	0	0	0	0	0	0	0
**S4-B-CTRL**	0	0	0	0	0	0	0	0	0	0	0	0	0	0	0	0
**S4-C-CTRL**	0	0	0	0	0	0	0	0	0	0	0	0	0	0	0	0
**S4-A-PPC**	0	0	0	0	0	0	0	0	0	0	0	0	0	0	0	0
**S4-B-PPC**	0	0	0	0	0	0	0	0	0	0	0	0	0	0	0	0
**S4-C-PPC**	0	0	0	0	0	0	0	0	0	0	0	0	0	0	0	0
**S4-A-PPI**	0	0	0	0	0	0	0	0	0	0	0	0	0	0	0	0
**S4-B-PPI**	0	0	0	0	0	0	0	0	0	0	0	0	0	0	0	0
**S4-C-PPI**	0	0	0	0	0	0	0	0	0	0	0	0	0	0	0	0
**S5-A-CTRL**	0	0	0	0	0	0	0.4	0	0	0	0	0	0.26	0	0	0
**S5-B-CTRL**	0	0	0	0	0	0	0	0	0	0	0	0	0	0	0	0
**S5-C-CTRL**	0	0	0	0	0	0	0.53	0	0	0	0	0	0	0	0	0
**S5-A-PPC**	0	0	0	0	0	0.27	0.98	0	0	0	0	0	0	0	0	0
**S5-B-PPC**	0	0	0	0	0	0	1.24	0	0	0	0	0	0	0	0	0
**S5-C-PPC**	0	0	0	0	0	0.32	0.79	0	0	0	0	0	0	0	0	0
**S5-A-PPI**	0	0	0	0	0	0	0.54	0	0	0	0	0	0	0	0	0
**S5-B-PPI**	0	0	0	0	0	0	0.74	0	0	0	0	0	0	0	0	0
**S5-C-PPI**	0	0	0	0	0	0.15	0.55	0	0	0	0	0	0	0	0	0
**S6-A-CTRL**	0	0	0	0	0	0	0	0	0	0	0	0	0	0	0	0
**S6-B-CTRL**	0	0	0	0	0	0	0	0	0	0	0	0	0	0	0	0
**S6-C-CTRL**	0	0	0	0	0	0	0	0	0	0	0	0	0	0	0	0
**S6-A-PPC**	0	0	0	0	0	0.04	0	0	0	0	0	0	0.12	0	0	0
**S6-B-PPC**	0	0	0	0	0	0.04	0	0	0	0	0	0	0.06	0	0	0
**S6-C-PPC**	0	0	0	0	0	0	0	0	0	0	0	0	0.11	0	0	0
**S6-A-PPI**	0	0	0	0	0	0.06	0	0	0	0	0	0	0	0	0	0
**S6-B-PPI**	0	0	0	0	0	0.1	0	0	0	0	0	0	0.06	0	0	0
**S6-C-PPI**	0	0	0	0	0	0.06	0	0	0	0	0	0	0.06	0	0	0

Bacterial species	Hallella multisaccharivorax	Harryflintia below species threshold	Heminiphilus below species threshold	Holdemania below species threshold	Holdemania filiformis	Holdemania massiliensis	Hominenteromicrobium below species threshold	Hominenteromicrobium mulieris	Hominifimenecus below species threshold	Hominifimenecus microfluidus	Hominilimicola below species threshold	Hominilimicola fabiformis	Hominisplanchenecus below species threshold	Hominisplanchenecus faecis	Hominiventricola aquisgranensis	Hominiventricola below species threshold
**S1-A-CTRL**	0	0	0	0	0	0	0	0	0	0	0	0	0	0	0	0
**S1-B-CTRL**	0	0	0	0	0	0	0	0	0	0	0	0	0	0	0	0
**S1-C-CTRL**	0	0	0	0	0	0	0	0	0	0	0	0	0	0	0	0
**S1-A-PPC**	0	0	0	0	0	0	0	0	0	0	0	0	0	0	0	0
**S1-B-PPC**	0	0	0	0	0	0	0	0	0	0	0	0	0	0	0	0
**S1-C-PPC**	0	0	0	0	0	0	0	0	0	0	0	0	0	0	0	0
**S1-A-PPI**	0	0	0	0	0	0	0	0	0	0	0	0	0	0	0	0
**S1-B-PPI**	0	0	0	0	0	0	0	0	0	0	0	0	0	0	0	0
**S1-C-PPI**	0	0	0	0	0	0	0	0	0	0	0	0	0	0	0	0
**S2-A-CTRL**	0	0	0	0	0	0	0	0.02	0	0	0	0.04	0	0	0	0
**S2-B-CTRL**	0	0	0	0	0	0	0	0.02	0	0	0	0.02	0	0	0	0
**S2-C-CTRL**	0	0	0	0	0	0	0	0.03	0	0	0	0.03	0	0	0	0
**S2-A-PPC**	0	0	0	0	0	0	0	0.14	0	0	0	0.03	0	0	0	0
**S2-B-PPC**	0	0	0	0	0	0.02	0	0.11	0	0	0	0.03	0	0	0	0
**S2-C-PPC**	0	0	0	0	0	0	0	0.05	0	0	0	0.03	0	0	0	0
**S2-A-PPI**	0	0	0	0	0	0	0	0.07	0	0	0	0.03	0	0	0	0
**S2-B-PPI**	0	0	0	0	0	0.02	0	0.04	0	0	0	0.03	0	0	0	0
**S2-C-PPI**	0	0	0	0	0	0	0	0.07	0	0	0	0.04	0	0	0	0
**S3-A-CTRL**	0	0	0	0	0	0	0	0	0	0	0	0	0	0	0	0
**S3-B-CTRL**	0	0	0	0	0	0	0	0	0	0	0	0	0	0	0	0
**S3-C-CTRL**	0	0	0	0	0	0	0	0	0	0	0	0	0	0	0	0
**S3-A-PPC**	0	0	0	0.02	0	0	0	0	0	0	0	0	0	0	0	0
**S3-B-PPC**	0	0	0	0.02	0	0	0	0	0	0	0	0	0	0	0	0
**S3-C-PPC**	0	0	0	0.02	0	0	0	0	0	0	0	0	0	0	0	0
**S3-A-PPI**	0	0	0	0	0	0	0	0	0	0	0	0	0	0	0	0
**S3-B-PPI**	0	0	0	0	0	0	0	0	0	0	0	0	0	0	0	0
**S3-C-PPI**	0	0	0	0	0	0	0	0	0	0	0	0	0	0	0	0
**S4-A-CTRL**	0	0	0	0	0	0	0	0	0	0	0	0	0	0	0	0
**S4-B-CTRL**	0	0	0	0	0	0	0	0	0	0	0	0	0	0	0	0
**S4-C-CTRL**	0	0	0	0	0	0	0	0	0	0	0	0	0	0	0	0
**S4-A-PPC**	0	0	0	0	0	0	0	0	0	0	0	0	0	0	0	0
**S4-B-PPC**	0	0	0	0	0	0	0	0	0	0	0	0	0	0	0	0
**S4-C-PPC**	0	0	0	0	0	0	0	0	0	0	0	0	0	0	0	0
**S4-A-PPI**	0	0	0	0	0	0	0	0	0	0	0	0	0	0	0	0
**S4-B-PPI**	0	0	0	0	0	0	0	0	0	0	0	0	0	0	0	0
**S4-C-PPI**	0	0	0	0	0	0	0	0	0	0	0	0	0	0	0	0
**S5-A-CTRL**	0	0	0	0	0	0	0	0	0	0	0	0	0	0	0	0
**S5-B-CTRL**	0	0	0	0	0	0	0	0	0	0	0	0	0	0	0	0
**S5-C-CTRL**	0	0	0	0	0	0	0	0	0	0	0	0	0	0	0	0
**S5-A-PPC**	0	0	0	0	0	0	0	0	0	0	0	0	0	0	0	0
**S5-B-PPC**	0	0	0	0	0	0	0	0	0	0	0	0	0	0	0	0
**S5-C-PPC**	0	0	0	0	0	0	0	0	0	0	0	0	0	0	0	0
**S5-A-PPI**	0	0	0	0	0	0	0	0	0	0	0	0	0	0	0	0
**S5-B-PPI**	0	0	0	0	0	0	0	0	0	0	0	0	0	0	0	0
**S5-C-PPI**	0	0	0	0	0	0	0	0	0	0	0	0	0	0	0	0
**S6-A-CTRL**	0	0	0	0	0	0	0	0	0	0	0	0	0	0	0	0
**S6-B-CTRL**	0	0	0	0	0	0	0	0	0	0	0	0	0	0	0	0
**S6-C-CTRL**	0	0	0	0	0	0	0	0	0	0	0	0	0	0	0	0
**S6-A-PPC**	0	0	0	0	0	0	0	0	0	0	0	0	0	0	0	0
**S6-B-PPC**	0	0	0	0	0	0	0	0	0	0	0	0	0	0	0	0
**S6-C-PPC**	0	0	0	0	0	0	0	0	0	0	0	0	0	0	0	0
**S6-A-PPI**	0	0	0	0	0	0	0	0	0	0	0	0	0	0	0	0
**S6-B-PPI**	0	0	0	0	0	0	0	0	0	0	0	0	0	0	0	0
**S6-C-PPI**	0	0	0	0	0	0	0	0	0	0	0	0	0	0.05	0	0.05

**Bacterial species**	Hominiventricola filiformis	Hoylesella below species threshold	Huintestinicola below species threshold	Huintestinicola butyrica	Hungatella below species threshold	Hungatella hathewayi	Hydrogeniiclostridium below species threshold	Hydrogeniiclostridium mannosilyticum	Hymenobacter below species threshold	Ihuprevotella below species threshold	Intestinibacillus below species threshold	Intestinimonas below species threshold	Intestinimonas butyriciproducens	Intestinimonas timonensis	Jilunia below species threshold	Jingyaoa below species threshold
**S1-A-CTRL**	0	0	0	0	0	0	0	0	0	0	0	0	0	0	0	0
**S1-B-CTRL**	0	0	0	0	0	0	0	0	0	0	0	0	0	0	0	0
**S1-C-CTRL**	0	0	0	0	0	0	0	0	0	0	0	0	0	0	0	0
**S1-A-PPC**	0	0	0	0	0	0	0	0	0	0	0	0	0	0	0	0
**S1-B-PPC**	0	0	0	0	0	0	0	0	0	0	0	0	0	0	0	0
**S1-C-PPC**	0	0	0	0	0	0	0	0	0	0	0	0	0	0	0	0
**S1-A-PPI**	0	0	0	0	0	0	0	0	0	0	0	0	0	0	0	0
**S1-B-PPI**	0	0	0	0	0	0	0	0	0	0	0	0	0	0	0	0
**S1-C-PPI**	0	0	0	0	0	0	0	0	0	0	0	0	0	0	0	0
**S2-A-CTRL**	0	0	0	0.03	0	0	0	0.03	0	0	0	0	0	0	0	0
**S2-B-CTRL**	0	0	0	0.02	0	0	0	0.02	0	0	0	0	0	0	0	0
**S2-C-CTRL**	0	0	0	0.02	0	0	0	0.03	0	0	0	0	0	0.02	0	0
**S2-A-PPC**	0	0	0.02	0.63	0	0	0	0.03	0	0	0	0	0	0	0	0.02
**S2-B-PPC**	0.02	0	0.02	0.47	0	0	0	0.02	0	0	0	0.02	0	0	0	0
**S2-C-PPC**	0	0	0	0.37	0	0	0	0.02	0	0	0	0	0	0	0	0
**S2-A-PPI**	0	0	0	0.51	0	0	0	0	0	0	0	0.02	0	0.02	0	0
**S2-B-PPI**	0	0	0.03	0.5	0	0	0	0.02	0	0	0	0	0	0.02	0	0
**S2-C-PPI**	0	0	0.03	0.58	0	0	0	0.04	0	0	0	0	0	0.02	0	0
**S3-A-CTRL**	0	0	0	0	0	0	0	0	0	0	0	0	0	0	0	0
**S3-B-CTRL**	0	0	0	0	0	0	0	0	0	0	0	0	0	0	0	0
**S3-C-CTRL**	0	0	0	0	0	0	0	0	0	0	0	0	0	0	0	0
**S3-A-PPC**	0	0.02	0	0.02	0	0	0	0	0	0	0	0	0.02	0	0	0
**S3-B-PPC**	0	0	0	0.02	0	0	0	0	0	0	0	0	0	0	0	0
**S3-C-PPC**	0	0	0	0.03	0	0	0	0	0	0	0	0	0	0	0	0
**S3-A-PPI**	0	0	0	0	0	0	0	0	0	0	0	0	0	0	0	0
**S3-B-PPI**	0	0	0	0.13	0	0	0	0	0	0	0	0	0	0	0	0
**S3-C-PPI**	0	0	0	0.16	0	0	0	0	0	0	0	0	0	0	0	0
**S4-A-CTRL**	0	0	0	0	0	0	0	0	0	0	0	0	0	0	0	0
**S4-B-CTRL**	0	0	0	0	0	0	0	0	0	0	0	0	0	0	0	0
**S4-C-CTRL**	0	0	0	0	0	0	0	0	0	0	0	0	0	0	0	0
**S4-A-PPC**	0	0	0	0	0	0	0	0	0	0	0	0	0	0	0	0
**S4-B-PPC**	0	0	0	0	0	0	0	0	0	0	0	0	0	0	0	0
**S4-C-PPC**	0	0	0	0	0	0	0	0	0	0	0	0	0	0	0	0
**S4-A-PPI**	0	0	0	0	0	0	0	0	0	0	0	0	0	0	0	0
**S4-B-PPI**	0	0	0	0	0	0	0	0	0	0	0	0	0	0	0	0
**S4-C-PPI**	0	0	0	0	0	0	0	0	0	0	0	0	0	0	0	0
**S5-A-CTRL**	0	0	0	0	0	0	0	0	0	0	0	0	0.5	0	0	0
**S5-B-CTRL**	0	0	0	0	0	0	0	0	0	0	0	0	0.74	0	0	0
**S5-C-CTRL**	0	0	0	0	0	0	0	0	0	0	0	0	0.71	0	0	0
**S5-A-PPC**	0	0	0	0	0	0	0	0	0	0	0	0	0.25	0	0	0
**S5-B-PPC**	0	0	0	0	0	0	0	0	0	0	0	0	0.39	0	0	0
**S5-C-PPC**	0	0	0	0	0	0	0	0	0	0	0	0	0.26	0	0	0
**S5-A-PPI**	0	0	0	0	0	0	0	0	0	0	0	0	0.21	0	0	0
**S5-B-PPI**	0	0	0	0	0	0	0	0	0	0	0	0	0.28	0	0	0
**S5-C-PPI**	0	0	0	0	0	0	0	0	0	0	0	0	0.13	0	0	0
**S6-A-CTRL**	0	0	0	0	0	0	0	0	0	0	0	0	0	0	0	0
**S6-B-CTRL**	0	0	0	0	0	0	0	0	0	0	0	0	0	0	0	0
**S6-C-CTRL**	0	0	0	0	0	0	0	0	0	0	0	0	0	0	0	0
**S6-A-PPC**	0.03	0	0	0	0	0	0	0	0	0	0	0	0	0	0	0
**S6-B-PPC**	0	0	0	0	0	0	0	0	0	0	0	0	0	0	0	0
**S6-C-PPC**	0	0	0	0	0	0	0	0	0	0	0	0.04	0	0	0	0
**S6-A-PPI**	0.06	0	0	0	0	0	0	0	0	0	0	0	0	0	0	0
**S6-B-PPI**	0.06	0	0	0	0	0	0	0	0	0	0	0.07	0	0	0	0
**S6-C-PPI**	0.06	0	0	0	0	0	0	0	0	0	0	0	0	0	0	0

Bacterial species	Jutongia below species threshold	Jutongia hominis	Jutongia huaianensis	Klebsiella aerogenes	Klebsiella below species threshold	Klebsiella grimontii	Klebsiella michiganensis	Klebsiella multihit species	Klebsiella oxytoca	Klebsiella pneumoniae	Klebsiella quasipneumoniae	Klebsiella spallanzanii	Klebsiella variicola	Kluyvera multihit species	Kosakonia below species threshold	Kosakonia sacchari
**S1-A-CTRL**	0	0	0	0	0	0	0	0.61	0	0	0	0	0.25	0	0	0.24
**S1-B-CTRL**	0	0	0	0	0	0	0	0.29	0	0	0	0	0.23	0	0.19	0.21
**S1-C-CTRL**	0	0	0	0	0	0	0	0.34	0	0	0	0	0	0	0	0.22
**S1-A-PPC**	0	0	0	0	0	0	0	0.34	0	0	0	0	0	0	0	0
**S1-B-PPC**	0	0	0	0	0.11	0	0	0.4	0	0	0	0	0	0	0	0
**S1-C-PPC**	0	0	0	0	0	0	0	0.4	0	0	0	0	0	0	0	0.11
**S1-A-PPI**	0	0	0	0	0	0	0	0.44	0	0	0	0	0	0	0	0.14
**S1-B-PPI**	0	0	0	0	0	0	0	0.34	0	0	0	0	0	0	0	0
**S1-C-PPI**	0	0	0	0	0	0	0	0.22	0	0	0	0	0	0	0	0
**S2-A-CTRL**	0	0.02	0	0.02	0.11	0.04	5.3	0.65	0.02	0.03	0.02	0.01	0.02	0	0	0
**S2-B-CTRL**	0	0	0	0.02	0.15	0.04	7.33	0.91	0.03	0.02	0.01	0.01	0.02	0.01	0	0
**S2-C-CTRL**	0	0.02	0	0.03	0.11	0.04	5.75	0.79	0.03	0.03	0.02	0.01	0.02	0	0	0
**S2-A-PPC**	0.06	0.09	0	0.02	0.05	0.02	1.82	0.29	0.01	0.02	0.02	0	0.01	0	0	0
**S2-B-PPC**	0.05	0.06	0	0	0.05	0.02	1.95	0.29	0	0.01	0.01	0	0.01	0	0	0
**S2-C-PPC**	0.04	0.06	0	0.01	0.04	0.01	1.56	0.23	0	0.01	0.01	0.01	0	0	0	0
**S2-A-PPI**	0.04	0.05	0	0.03	0.12	0.03	6.61	0.89	0.03	0.02	0.02	0.01	0.02	0	0	0
**S2-B-PPI**	0.04	0.06	0	0.03	0.18	0.05	8.02	0.98	0.03	0.04	0.02	0.02	0.04	0.02	0	0
**S2-C-PPI**	0.03	0.06	0	0.04	0.15	0.05	7.29	0.87	0.04	0.05	0.02	0.01	0.03	0.01	0	0
**S3-A-CTRL**	0	0	0	0	0	0	0	0.17	0	0	0	0	0	0	0	0
**S3-B-CTRL**	0	0	0	0	0	0	0	0.24	0	0	0	0	0	0	0	0
**S3-C-CTRL**	0	0	0	0	0	0	0	0.22	0	0	0	0	0	0	0	0
**S3-A-PPC**	0	0	0	0	0	0	0	0.04	0	0	0	0	0	0	0	0
**S3-B-PPC**	0	0	0	0	0	0	0	0.05	0	0	0	0	0	0	0	0
**S3-C-PPC**	0	0	0	0	0	0	0	0.04	0	0	0	0	0	0	0	0
**S3-A-PPI**	0	0	0	0	0	0	0	0.12	0	0	0	0	0	0	0	0
**S3-B-PPI**	0	0	0	0	0	0	0	0.17	0	0	0	0	0	0	0	0
**S3-C-PPI**	0	0	0	0	0	0	0	0.18	0	0	0	0	0	0	0	0
**S4-A-CTRL**	0	0	0	0	0	0	0	0	0	0	0	0	0	0	0	0
**S4-B-CTRL**	0	0	0	0	0	0	0	0	0	0	0	0	0	0	0	0
**S4-C-CTRL**	0	0	0	0	0	0	0	0	0	0	0	0	0	0	0	0
**S4-A-PPC**	0.35	0	0	0	0	0	0	0	0	0	0	0	0	0	0	0
**S4-B-PPC**	0	0	0	0	0	0	0	0	0	0	0	0	0	0	0	0
**S4-C-PPC**	0	0	0	0	0	0	0	0	0	0	0	0	0	0	0	0
**S4-A-PPI**	0	0	0	0	0	0	0	0	0	0	0	0	0	0	0	0
**S4-B-PPI**	0	0	0	0	0	0	0	0	0	0	0	0	0	0	0	0
**S4-C-PPI**	0	0	0	0	0	0	0	0	0	0	0	0	0	0	0	0
**S5-A-CTRL**	0	0	0	0	0	0	0	0	0	0	0	0	0	0	0	0
**S5-B-CTRL**	0	0	0	0	0	0	0	0	0	0	0	0	0	0	0	0
**S5-C-CTRL**	0	0	0	0	0	0	0	0	0	0	0	0	0	0	0	0
**S5-A-PPC**	0	0	0	0	0	0	0	0	0	0	0	0	0	0	0	0
**S5-B-PPC**	0	0	0	0	0	0	0	0	0	0	0	0	0	0	0	0
**S5-C-PPC**	0	0	0	0	0	0	0	0	0	0	0	0	0	0	0	0
**S5-A-PPI**	0	0	0	0	0	0	0	0	0	0	0	0	0	0	0	0
**S5-B-PPI**	0	0	0	0	0	0	0	0	0	0	0	0	0	0	0	0
**S5-C-PPI**	0	0	0	0	0	0	0	0	0	0	0	0	0	0	0	0
**S6-A-CTRL**	0	0	0	0	0	0	0.09	0	0	0	0	0	0	0	0	0
**S6-B-CTRL**	0	0	0	0	0	0	0	0	0	0	0	0	0	0	0	0
**S6-C-CTRL**	0	0	0	0	0	0	0	0	0	0	0	0	0	0	0	0
**S6-A-PPC**	0	0	0	0	0	0	0	0.02	0	0	0	0	0	0	0	0
**S6-B-PPC**	0	0	0	0	0	0	0	0	0	0	0	0	0	0	0	0
**S6-C-PPC**	0	0	0	0	0	0	0	0	0	0	0	0	0	0	0	0
**S6-A-PPI**	0	0	0	0	0	0	0	0	0	0	0	0	0	0	0	0
**S6-B-PPI**	0	0	0	0	0	0	0.03	0.05	0	0	0	0	0	0	0	0
**S6-C-PPI**	0	0	0	0	0	0	0.03	0.04	0	0	0	0	0	0	0	0

**Bacterial species**	Lachnoclostridium below species threshold	Lachnospira below species threshold	Lachnospira eligens	Lacrimispora below species threshold	Lacrimispora saccharolytica	Lactobacillus below species threshold	Lactobacillus crispatus	Lactobacillus gasseri	Lactobacillus iners	Lactococcus below species threshold	Lactococcus cremoris	Lactococcus lactis	Lactonifactor below species threshold	Laedolimicola below species threshold	Laedolimicola intestinihominis	Lawsonibacter below species threshold
**S1-A-CTRL**	0	0	0	0	0	0	0	0	0	0	0	0	0	0	0	0
**S1-B-CTRL**	0	0	0	0	0	0	0	0	0	0	0	0	0	0	0	0
**S1-C-CTRL**	0	0	0	0	0	0	0	0	0	0	0	0	0	0	0	0
**S1-A-PPC**	0	0	0	0	0	0	0	0	0	0	0	0	0	0	0	0
**S1-B-PPC**	0	0	0	0	0	0	0	0	0	0	0	0	0	0	0	0
**S1-C-PPC**	0	0	0	0	0	0	0	0	0	0	0	0	0	0	0	0
**S1-A-PPI**	0	0	0	0	0	0	0	0	0	0	0	0	0	0	0	0
**S1-B-PPI**	0	0	0	0	0	0	0	0	0	0	0	0	0	0	0	0
**S1-C-PPI**	0	0	0	0	0	0	0	0	0	0	0	0	0	0	0	0
**S2-A-CTRL**	0	0.02	0.09	0	0	0	0	0	0	0	0	0	0	0	0	0
**S2-B-CTRL**	0	0.02	0.09	0	0	0	0	0	0	0	0	0	0	0	0	0
**S2-C-CTRL**	0	0.01	0.11	0	0	0	0	0	0	0	0	0	0	0	0	0
**S2-A-PPC**	0	0.04	0.4	0	0	0	0	0	0.06	0	0	0	0	0	0	0
**S2-B-PPC**	0	0.04	0.33	0	0	0	0	0	0	0	0	0	0	0	0	0.02
**S2-C-PPC**	0	0.03	0.18	0	0	0	0	0	0	0	0	0	0	0	0	0
**S2-A-PPI**	0	0	0.17	0	0	0	0	0	0	0	0	0	0	0	0	0
**S2-B-PPI**	0	0.05	0.3	0	0	0	0	0	0.05	0	0	0	0	0	0	0
**S2-C-PPI**	0	0.02	0.22	0	0	0	0	0	0	0	0	0	0	0	0	0
**S3-A-CTRL**	0	0.27	0	0	0	0	0	0	0	0	0	0	0	0	0	0
**S3-B-CTRL**	0	0	0	0	0	0	0	0	0	0	0	0	0	0	0	0
**S3-C-CTRL**	0	0	0	0	0	0	0	0	0	0	0	0	0	0	0	0
**S3-A-PPC**	0	0	0.02	0	0	0	0	0	0	0.03	0	0	0	0	0	0
**S3-B-PPC**	0	0	0.02	0	0	0	0	0	0	0.06	0	0	0	0	0	0
**S3-C-PPC**	0	0	0	0	0	0	0	0	0	0.03	0	0	0	0	0	0
**S3-A-PPI**	0	0	0	0	0	0	0	0	0	0.2	0	0	0	0	0	0
**S3-B-PPI**	0	0	0.15	0	0	0	0	0	0	0.14	0	0	0	0	0	0
**S3-C-PPI**	0	0	0	0	0	0	0	0	0	0.17	0	0	0	0	0	0
**S4-A-CTRL**	0	0	0	0	0	0	0	0	0	0	0	0	0	0	0	0
**S4-B-CTRL**	0	0	0	0	0	0	0	0	0	0	0	0	0	0	0	0
**S4-C-CTRL**	0	0	0	0	0	0	0	0	0	0	0	0	0	0	0	0
**S4-A-PPC**	0	0	0.47	0	0	0	0	0	0	0	0	0	0	0	0	0
**S4-B-PPC**	0	0	0.65	0	0	0	0	0	0	0	0	0	0	0	0	0
**S4-C-PPC**	0	0	0	0	0	0	0	0	0	0	0	0	0	0	0	0
**S4-A-PPI**	0	0	0.11	0	0	0	0	0	0	0.15	0	0	0	0	0	0
**S4-B-PPI**	0	0	0.07	0	0	0	0	0	0	0.08	0	0	0	0	0	0
**S4-C-PPI**	0	0	0	0	0	0	0	0	0	0.17	0	0	0	0	0	0
**S5-A-CTRL**	0	0	0	0	0	0	0	0	0	0	0	0	0	0	0	0
**S5-B-CTRL**	0	0	0	0	0	0	0	0	0	0	0	0	0	0	0	0
**S5-C-CTRL**	0	0	0	0	0	0	0	0	0	0	0	0	0	0	0	0
**S5-A-PPC**	0	0	0	0	0	0	0.91	0	0	0	0	0	0	0	0	0
**S5-B-PPC**	0	0	0	0	0	0	1.07	0	0	0	0	0	0	0	0	0
**S5-C-PPC**	0	0	0	0	0	0	1.18	0	0	0	0	0	0	0	0	0
**S5-A-PPI**	0	0	0	0	0	0	0	0	0	0	0	0	0	0	0	0
**S5-B-PPI**	0	0	0	0	0	0	0	0	0	0	0	0	0	0	0	0
**S5-C-PPI**	0	0	0	0	0	0	0	0	0	0	0	0	0	0	0	0
**S6-A-CTRL**	0	0.18	0	0	0	0	0	0	0	0	0	0	0	0	0	0
**S6-B-CTRL**	0	0.32	0	0	0	0	0	0	0	0	0	0	0	0	0	0
**S6-C-CTRL**	0	0.41	0	0	0	0	0	0	0	0	0	0	0	0	0	0
**S6-A-PPC**	0	0	0.09	0	0	0.13	0	0.22	0	0	0.16	0.07	0	0	0	0
**S6-B-PPC**	0	0.05	0.14	0	0	0.06	0	0.18	0	0	0.2	0.04	0	0	0	0
**S6-C-PPC**	0	0.03	0.15	0	0	0.06	0	0.22	0	0	0.16	0.04	0	0	0	0
**S6-A-PPI**	0	0	0.16	0	0	0.13	0	0.52	0	0	0.11	0.09	0	0	0	0
**S6-B-PPI**	0	0	0.23	0	0	0.11	0	0.35	0	0	0	0	0	0	0	0
**S6-C-PPI**	0	0.07	0.33	0	0	0.19	0	0.6	0	0	0.09	0	0	0	0	0

Bacterial species	Lawsonibacter faecis	Lawsonibacter hominis	Leclercia adecarboxylata	Leclercia below species threshold	Lepagella below species threshold	Leuconostoc lactis	Leuconostoc multihit species	Leyella below species threshold	Leyella lascolaii	Leyella stercorea	Ligaoa below species threshold	Ligilactobacillus animalis	Ligilactobacillus below species threshold	Ligilactobacillus multihit species	Ligilactobacillus murinus	Limosilactobacillus below species threshold
**S1-A-CTRL**	0	0	0	0	0	0	0	0	0	0	0	0	0	0	0	0
**S1-B-CTRL**	0	0	0	0	0	0	0	0	0	0	0	0	0	0	0	0
**S1-C-CTRL**	0	0	0	0	0	0	0	0	0	0	0	0	0	0	0	0
**S1-A-PPC**	0	0	0	0	0	0	0	0	0	0	0	0	0	0	0	0
**S1-B-PPC**	0	0	0	0	0	0	0	0	0	0	0	0	0	0	0	0
**S1-C-PPC**	0	0	0	0	0	0	0	0	0	0	0	0	0	0	0	0
**S1-A-PPI**	0	0	0	0	0	0	0	0	0	0	0	0	0	0	0	0
**S1-B-PPI**	0	0	0	0	0	0	0	0	0	0	0	0	0	0	0	0
**S1-C-PPI**	0	0	0	0	0	0	0	0	0	0	0	0	0	0	0	0
**S2-A-CTRL**	0	0	0.07	0.03	0	0	0	0	0	0	0	0	0	0	0	0
**S2-B-CTRL**	0	0	0.12	0.03	0	0	0	0	0	0	0	0	0	0	0	0
**S2-C-CTRL**	0	0	0.18	0.04	0	0	0	0	0	0	0	0	0	0	0	0
**S2-A-PPC**	0	0	0.05	0.02	0	0	0	0	0	0	0	0	0	0	0	0
**S2-B-PPC**	0	0	0.05	0.02	0	0	0	0	0	0	0	0	0	0	0	0
**S2-C-PPC**	0	0	0.06	0.03	0	0	0	0	0	0	0	0	0	0	0	0
**S2-A-PPI**	0	0	0.15	0.04	0	0	0	0	0	0	0	0	0	0	0	0
**S2-B-PPI**	0	0	0.11	0.03	0	0	0	0	0	0	0	0	0	0	0	0
**S2-C-PPI**	0	0	0.14	0.04	0	0	0	0	0	0	0	0	0	0	0	0
**S3-A-CTRL**	0	0	0	0	0	2.29	0	0.15	0	0	0	0	0	0	0	0
**S3-B-CTRL**	0	0	0	0	0	2.27	0	0.23	0	0	0	0	0	0	0	0
**S3-C-CTRL**	0	0	0	0	0	3.05	0	0.32	0	0	0	0	0	0	0	0
**S3-A-PPC**	0	0	0	0	0	1.91	0	0.05	0	0	0	0	0	0	0	0
**S3-B-PPC**	0	0	0	0	0	1.63	0.03	0.05	0	0	0	0	0	0	0	0.04
**S3-C-PPC**	0	0	0	0	0	2.3	0	0.04	0	0	0	0	0	0	0	0.03
**S3-A-PPI**	0	0	0	0	0	6.24	0	0.14	0	0	0	0	0	0	0	0
**S3-B-PPI**	0	0	0	0	0	8.77	0.22	0.09	0	0	0	0	0	0	0	0
**S3-C-PPI**	0	0	0	0	0	6.51	0	0.16	0	0	0	0	0	0	0	0
**S4-A-CTRL**	0	0	0	0	0	0	0	0	0	0	0	0	0	0	0	0
**S4-B-CTRL**	0	0	0	0	0	0	0	0	0	0	0	0	0	0	0	0
**S4-C-CTRL**	0	0	0	0	0	0	0	0	0	0	0	0	0	0	0	0
**S4-A-PPC**	0	0	0	0	0	0	0	0	0	0	0	0	0	0	0	0
**S4-B-PPC**	0	0	0	0	0	0	0	0	0	0	0	0	0	0	0	0
**S4-C-PPC**	0	0	0	0	0	0	0	0	0	0	0	0	0	0	0	0
**S4-A-PPI**	0	0	0	0	0	0	0	0	0	0	0	0	0	0	0	0
**S4-B-PPI**	0	0	0	0	0	0	0	0	0	0	0	0	0	0	0	0
**S4-C-PPI**	0	0	0	0	0	0	0	0	0	0	0	0	0	0	0	0
**S5-A-CTRL**	0	0	0	0	0	0	0	0	0	0	0	0	0	0	0	0
**S5-B-CTRL**	0	0	0	0	0	0	0	0	0	0	0	0	0	0	0	0
**S5-C-CTRL**	0	0	0	0	0	0	0	0	0	0	0	0	0	0	0	0
**S5-A-PPC**	0	0	0	0	0	0	0	0	0	0	0	0	0	0	0	0
**S5-B-PPC**	0	0	0	0	0	0	0	0	0	0	0	0	0	0	0	0
**S5-C-PPC**	0	0	0	0	0	0	0	0	0	0	0	0	0	0	0	0
**S5-A-PPI**	0	0	0	0	0	0	0	0	0	0	0	0	0	0	0	0
**S5-B-PPI**	0	0	0	0	0	0	0	0	0	0	0	0	0	0	0	0
**S5-C-PPI**	0	0	0	0	0	0	0	0	0	0	0	0	0	0	0	0
**S6-A-CTRL**	0	0	0	0	0	0	0	0	0	0	0	6.97	0.43	0	0	0
**S6-B-CTRL**	0	0	0	0	0	0	0	0	0	0	0	6.22	0.34	0	0	0
**S6-C-CTRL**	0	0	0	0	0	0	0	0	0	0	0	9.97	0.72	0	0	0
**S6-A-PPC**	0	0	0	0	0	0	0	0	0	0	0	43.61	2.23	0.52	0.76	0
**S6-B-PPC**	0	0	0	0	0	0	0	0	0	0	0	40.54	2.15	0.38	0.67	0
**S6-C-PPC**	0	0	0	0	0	0	0	0	0	0	0	33.13	1.87	0.39	0.53	0
**S6-A-PPI**	0	0	0	0	0	0	0	0	0	0	0	17.53	1.01	0.2	0.41	0
**S6-B-PPI**	0	0	0	0	0	0	0	0	0	0	0	4.87	0.25	0.1	0.09	0
**S6-C-PPI**	0	0	0	0	0	0	0	0	0	0	0	8.25	0.36	0.1	0.16	0

**Bacterial species**	Limosilactobacillus fermentum	Listeria below species threshold	Luxibacter below species threshold	Maccoya below species threshold	Maccoya intestinihominis	Magnetospirillum below species threshold	Mailhella below species threshold	Maliibacterium below species threshold	Marasmitruncus below species threshold	Marvinbryantia below species threshold	Massiliimalia below species threshold	Massilistercora below species threshold	Massilistercora timonensis	Mediterranea below species threshold	Mediterranea massiliensis	Mediterraneibacter below species threshold
**S1-A-CTRL**	0	0	0	0	0	0	0	0	0	0	0	0	0	0	0	0
**S1-B-CTRL**	0	0	0	0	0	0	0	0	0	0	0	0	0	0	0	0
**S1-C-CTRL**	0	0	0	0	0	0	0	0	0	0	0	0	0	0	0	0
**S1-A-PPC**	0	0	0	0	0	0	0	0	0	0	0	0	0	0	0	0
**S1-B-PPC**	0	0	0	0	0	0	0	0	0	0	0	0	0	0	0	0.16
**S1-C-PPC**	0	0	0	0	0	0	0	0	0	0	0	0	0	0	0	0.16
**S1-A-PPI**	0	0	0	0	0	0	0	0	0	0	0	0	0	0	0	0
**S1-B-PPI**	0	0	0	0	0	0	0	0	0	0	0	0	0	0	0	0
**S1-C-PPI**	0	0	0	0	0	0	0	0	0	0	0	0	0	0	0	0.12
**S2-A-CTRL**	0	0	0	0	0	0	0	0	0	0	0	0	0	0	0	0
**S2-B-CTRL**	0	0	0	0	0	0	0	0	0	0	0	0	0	0	0	0
**S2-C-CTRL**	0	0	0	0	0	0	0	0	0	0	0	0	0	0	0	0
**S2-A-PPC**	0	0	0	0	0	0	0	0	0	0	0	0	0	0	0	0.02
**S2-B-PPC**	0	0	0	0	0	0	0	0	0	0	0	0	0	0	0	0
**S2-C-PPC**	0	0	0	0	0	0	0	0	0	0	0	0	0	0	0	0
**S2-A-PPI**	0	0	0	0	0	0	0	0	0	0	0	0	0	0	0	0.03
**S2-B-PPI**	0	0	0	0	0	0	0	0	0	0	0	0	0	0	0	0
**S2-C-PPI**	0	0	0	0	0	0	0	0	0	0	0	0	0	0	0	0
**S3-A-CTRL**	0	0	0	0	0	0	0	0	0	0	0	0	0	0.17	0	0
**S3-B-CTRL**	0	0	0	0	0	0	0	0	0	0	0	0	0	0	0	0
**S3-C-CTRL**	0	0	0	0	0	0	0	0	0	0	0	0	0	0	0	0
**S3-A-PPC**	0	0	0	0	0	0	0	0	0	0	0	0	0.03	0	0	0.02
**S3-B-PPC**	0	0	0	0	0	0	0	0	0	0	0	0	0	0	0	0
**S3-C-PPC**	0	0	0	0	0	0	0	0	0	0	0	0	0	0	0	0
**S3-A-PPI**	0	0	0	0	0	0	0	0	0	0	0	0	0	0	0	0
**S3-B-PPI**	0	0	0	0	0	0	0	0	0	0	0	0	0	0	0	0
**S3-C-PPI**	0	0	0	0	0	0	0	0	0	0	0	0	0	0	0	0
**S4-A-CTRL**	0	0	0	0	0	0	0	0	0	0	0	0	0	0	0	0
**S4-B-CTRL**	0	0	0	0	0	0	0	0	0	0	0	0	0	0	0	0
**S4-C-CTRL**	0	0	0	0	0	0	0	0	0	0	0	0	0	0	0	0
**S4-A-PPC**	0	0	0	0	0	0	0	0	0	0	0	0	0	0	0	0
**S4-B-PPC**	0	0	0	0	0	0	0	0	0	0	0	0	0	0	0	0
**S4-C-PPC**	0	0	0	0	0	0	0	0	0	0	0	0	0	0	0	0
**S4-A-PPI**	0	0	0	0	0	0	0	0	0	0	0	0	0	0	0	0
**S4-B-PPI**	0	0	0	0	0	0	0	0	0	0	0	0	0	0	0	0
**S4-C-PPI**	0	0	0	0	0	0	0	0	0	0	0	0	0	0	0	0
**S5-A-CTRL**	0	0	0	0	0	0	0	0	0	0	0	0	0	0	0	0
**S5-B-CTRL**	0	0	0	0	0	0	0	0	0	0	0	0	0	0	0	0
**S5-C-CTRL**	0	0	0	0	0	0	0	0	0	0	0	0	0	0	0	0
**S5-A-PPC**	0	0	0	0	0	0	0	0	0	0	0	0	0	0	0	0
**S5-B-PPC**	0	0	0	0	0	0	0	0	0	0	0	0	0	0	0	0
**S5-C-PPC**	0	0	0	0	0	0	0	0	0	0	0	0	0	0	0	0
**S5-A-PPI**	0	0	0	0	0	0	0	0	0	0	0	0	0	0	0	0
**S5-B-PPI**	0	0	0	0	0	0	0	0	0	0	0	0	0	0	0	0
**S5-C-PPI**	0	0	0	0	0	0	0	0	0	0	0	0	0	0	0	0
**S6-A-CTRL**	0	0	0	0	0	0	0	0	0	0	0	0	0	0	0	0
**S6-B-CTRL**	0	0	0	0	0	0	0	0	0	0	0	0	0	0	0	0
**S6-C-CTRL**	0	0	0	0	0	0	0	0	0	0	0	0	0	0	0	0
**S6-A-PPC**	0.39	0	0	0	0	0	0	0	0	0	0	0	0	0	0	0
**S6-B-PPC**	0.33	0	0	0	0	0	0	0	0	0	0	0	0	0	0	0
**S6-C-PPC**	0.27	0	0	0	0	0	0	0	0	0	0	0	0	0	0	0
**S6-A-PPI**	0.76	0	0	0	0	0	0	0	0	0	0	0	0	0	0	0
**S6-B-PPI**	1.35	0	0	0	0	0	0	0	0	0	0	0	0	0	0	0
**S6-C-PPI**	1.42	0	0	0	0	0	0	0	0	0	0	0	0	0	0	0

**Bacterial species**	Mediterraneibacter faecis	Mediterraneibacter gnavus	Mediterraneibacter multihit species	Megasphaera below species threshold	Megasphaera elsdenii	Megasphaera massiliensis	Merdimmobilis below species threshold	Merdimmobilis hominis	Merdimonas below species threshold	Merdimonas faecis	Mesosutterella below species threshold	Mesosutterella multiformis	Millionella below species threshold	Mitsuokella below species threshold	Mitsuokella jalaludinii	Mitsuokella multacida
**S1-A-CTRL**	0	1.76	0	0	0	0	0	0	0	0	0	0	0	0	0	0
**S1-B-CTRL**	0	1.98	0	0	0	0	0	0	0	0	0	0	0	0	0	0
**S1-C-CTRL**	0	2.48	0	0	0	0	0	0	0	0	0	0	0	0	0	0
**S1-A-PPC**	0	1.45	0	0	0	0	0	0	0	0	0	0	0	0	0	0
**S1-B-PPC**	0	1.11	0	0	0	0	0	0	0	0	0	0	0	0	0	0
**S1-C-PPC**	0	1.2	0	0	0	0	0	0	0	0	0	0	0	0	0	0
**S1-A-PPI**	0	2.31	0	0	0	0	0	0	0	0	0	0	0	0	0	0
**S1-B-PPI**	0	3.09	0	0	0	0	0	0	0	0	0	0	0	0	0	0
**S1-C-PPI**	0	2.5	0	0	0	0	0	0	0	0	0	0	0	0	0	0
**S2-A-CTRL**	0.02	0	0	0	0	0	0	0	0	0	0	0	0	0	0	0
**S2-B-CTRL**	0	0	0	0	0	0	0	0	0	0	0	0	0	0	0	0
**S2-C-CTRL**	0.02	0	0	0	0	0	0	0	0	0	0	0	0	0	0	0
**S2-A-PPC**	0	0	0	0	0	0	0	0	0	0	0	0	0	0	0	0
**S2-B-PPC**	0.03	0	0	0	0	0	0	0	0	0	0	0	0	0	0	0
**S2-C-PPC**	0	0	0	0	0	0	0	0	0	0	0	0	0	0	0	0
**S2-A-PPI**	0.03	0	0	0	0	0	0	0	0	0	0	0	0	0	0	0
**S2-B-PPI**	0.04	0	0	0	0	0	0	0	0	0	0	0	0	0	0	0
**S2-C-PPI**	0.05	0	0	0	0	0	0	0	0	0	0	0	0	0	0	0
**S3-A-CTRL**	0	0	0	0	0	0	0	0	0	0	0	2.45	0	0	0	0
**S3-B-CTRL**	0	0	0	0	0	0	0	0	0	0	0	7.26	0	0	0	0
**S3-C-CTRL**	0	0	0	0	0	0	0	0	0	0	0.45	6.14	0	0	0	0
**S3-A-PPC**	0.03	0	0	0	0	0.02	0	0	0	0.04	0	0.15	0	0	0	0
**S3-B-PPC**	0	0	0	0	0	0	0	0	0	0.03	0	0.09	0	0	0	0
**S3-C-PPC**	0.02	0	0	0	0	0	0	0	0	0.02	0	0.13	0	0	0	0
**S3-A-PPI**	0	0	0	0	0	0.46	0	0	0	0.16	0.28	7.44	0	0	0	0
**S3-B-PPI**	0	0	0	0	0	0.12	0	0	0	0.12	0	0.28	0	0	0	0
**S3-C-PPI**	0	0	0	0	0	0	0	0	0	0	0	3.14	0	0	0	0
**S4-A-CTRL**	0	0	0	0	0	0	0	0	0	0	0	1.22	0	0	0	0
**S4-B-CTRL**	0	0	0	0	0	0	0	0	0	0	0	1.69	0	0	0	0
**S4-C-CTRL**	0	0	0	0	0	0	0	0	0	0	0.11	4.15	0	0	0	0
**S4-A-PPC**	0	0	0	0	0	0	0	0	0	0	0	9.8	0	0	0	0
**S4-B-PPC**	0	0	0	0	0	0	0	0	0	0	0	11.24	0	0	0	0
**S4-C-PPC**	0	0	0	0	0	0	0	0	0	0	0.58	15.86	0	0	0	0
**S4-A-PPI**	0	0	0	0	0	0	0	0	0	0	0.26	7.1	0	0	0	0
**S4-B-PPI**	0	0	0	0	0	0	0	0	0	0	0.53	13.07	0	0	0	0
**S4-C-PPI**	0	0	0	0	0	0	0	0	0	0	0.11	2.5	0	0	0	0
**S5-A-CTRL**	0	0	0	0	0	0	0	0	0	0	0	0	0	0	0	0
**S5-B-CTRL**	0	0	0	0	0	0	0	0	0	0	0	0	0	0	0	0
**S5-C-CTRL**	0	0	0	0	0	0	0	0	0	0	0	0	0	0	0	0
**S5-A-PPC**	0	0	0	0	0	0	0	0	0	0	0	0	0	0	0	0
**S5-B-PPC**	0	0	0	0	0	0	0	0	0	0	0	0	0	0	0	0
**S5-C-PPC**	0	0	0	0	0	0	0	0	0	0	0	0	0	0	0	0
**S5-A-PPI**	0	0	0	0	0	0	0	0	0	0	0	0	0	0	0	0
**S5-B-PPI**	0	0	0	0	0	0	0	0	0	0	0	0	0	0	0	0
**S5-C-PPI**	0	0	0	0	0	0	0	0	0	0	0	0	0	0	0	0
**S6-A-CTRL**	0	0	0	0.28	0	0	0	0	0	0	0	0	0	3.78	2.35	0.35
**S6-B-CTRL**	0	0	0	0	0	0	0	0	0	0	0	0	0	3.1	0.96	0.29
**S6-C-CTRL**	0	0	0	0.25	0	0	0	0	0	0	0	0	0	1.21	0.23	0
**S6-A-PPC**	0.03	0	0	0.03	0	0	0	0	0	0	0	0	0	0.06	0.05	0
**S6-B-PPC**	0	0	0	0.05	0.05	0	0	0	0	0	0	0	0	0.09	0.07	0
**S6-C-PPC**	0	0	0	0.07	0.04	0	0	0	0	0	0	0	0	0.1	0.07	0
**S6-A-PPI**	0	0	0	0.2	0.1	0	0	0	0	0	0	0	0	1.05	0.74	0.1
**S6-B-PPI**	0	0	0	0.12	0.12	0	0	0	0	0	0	0	0	2.12	1.79	0.19
**S6-C-PPI**	0	0	0	0.14	0.12	0	0	0	0	0	0	0	0	1.48	0.77	0.16

**Bacterial species**	Mitsuokella multihit species	Mobilibacterium below species threshold	Moryella below species threshold	Murdochiella vaginalis	Muribaculum below species threshold	Murimonas below species threshold	Muriventricola aceti	Muriventricola below species threshold	Negativibacillus below species threshold	Negativibacillus massiliensis	Neglectibacter below species threshold	Neobittarella below species threshold	Neopoerus below species threshold	Neopoerus faecalis	Nitratidesulfovibrio below species threshold	Nocardioides below species threshold
**S1-A-CTRL**	0	0	0	0	0	0	0	0	0	0	0	0	0	0	0	0
**S1-B-CTRL**	0	0	0	0	0	0	0	0	0	0	0	0	0	0	0	0
**S1-C-CTRL**	0	0	0	0	0	0	0	0	0	0	0	0	0	0	0	0
**S1-A-PPC**	0	0	0	0	0	0	0	0	0	0	0	0	0	0	0	0
**S1-B-PPC**	0	0	0	0	0	0	0	0	0	0	0	0	0	0	0	0
**S1-C-PPC**	0	0	0	0	0	0	0	0	0	0	0	0	0	0	0	0
**S1-A-PPI**	0	0	0	0	0	0	0	0	0	0	0	0	0	0	0	0
**S1-B-PPI**	0	0	0	0	0	0	0	0	0	0	0	0	0	0	0	0
**S1-C-PPI**	0	0	0	0	0	0	0	0	0	0	0	0	0	0	0	0
**S2-A-CTRL**	0	0	0	0	0	0	0	0	0	0	0	0	0	0.04	0	0
**S2-B-CTRL**	0	0	0	0	0	0	0	0	0	0	0	0	0	0.02	0	0
**S2-C-CTRL**	0	0	0	0	0	0	0	0	0	0	0	0	0	0.04	0	0
**S2-A-PPC**	0	0	0	0	0	0	0	0	0	0	0	0	0	0.06	0	0
**S2-B-PPC**	0	0	0	0	0	0	0	0	0	0	0	0	0	0.04	0	0
**S2-C-PPC**	0	0	0	0	0	0	0	0	0	0	0	0	0	0.02	0	0
**S2-A-PPI**	0	0	0	0	0	0	0	0	0	0	0	0	0	0.1	0	0
**S2-B-PPI**	0	0	0	0	0	0	0	0	0	0	0	0	0	0.05	0	0
**S2-C-PPI**	0	0	0	0	0	0	0	0	0	0	0	0	0	0.07	0	0
**S3-A-CTRL**	0	0	0	0	0	0	0	0	0	0	0	0	0	0	0	0
**S3-B-CTRL**	0	0	0	0	0	0	0	0	0	0	0	0	0	0	0	0
**S3-C-CTRL**	0	0	0	0	0	0	0	0	0	0	0	0	0	0	0	0
**S3-A-PPC**	0	0	0	0.1	0	0	0	0	0	0	0	0	0.02	0	0	0
**S3-B-PPC**	0	0	0	0.12	0	0	0	0	0	0	0	0	0	0	0	0
**S3-C-PPC**	0	0	0	0.15	0	0	0	0	0	0	0	0	0	0	0	0
**S3-A-PPI**	0	0	0	0	0	0	0	0	0	0	0	0	0	0	0	0
**S3-B-PPI**	0	0	0	0	0	0	0	0	0	0	0	0	0	0	0	0
**S3-C-PPI**	0	0	0	0	0	0	0	0	0	0	0	0	0	0	0	0
**S4-A-CTRL**	0	0	0	0	0	0	0	0	0	0	0	0	0	0	0	0
**S4-B-CTRL**	0	0	0	0	0.07	0	0	0	0	0	0	0	0	0	0	0
**S4-C-CTRL**	0	0	0	0	0	0	0	0	0	0	0	0	0	0	0	0
**S4-A-PPC**	0	0	0	0	0	0	0	0	0	0	0	0	0	0	0	0
**S4-B-PPC**	0	0	0	0	0	0	0	0	0	0	0	0	0	0	0	0
**S4-C-PPC**	0	0	0	0	0	0	0	0	0	0	0	0	0	0	0	0
**S4-A-PPI**	0	0	0	0	0	0	0	0	0	0	0	0	0	0	0	0
**S4-B-PPI**	0	0	0	0	0	0	0	0	0	0	0	0	0	0	0	0
**S4-C-PPI**	0	0	0	0	0	0	0	0	0	0	0	0	0	0	0	0
**S5-A-CTRL**	0	0	0	0	0	0	0	0	0	0	0	0	0	0	0	0
**S5-B-CTRL**	0	0	0	0	0	0	0	0	0	0	0	0	0	0	0	0.24
**S5-C-CTRL**	0	0	0	0	0	0	0	0	0	0	0	0	0	0	0	0
**S5-A-PPC**	0	0	0	0	0	0	0	0	0	0	0	0	0	0	0	0
**S5-B-PPC**	0	0	0	0	0	0	0	0	0	0	0	0	0	0	0	0
**S5-C-PPC**	0	0	0	0	0	0	0	0	0	0	0	0	0	0	0	0
**S5-A-PPI**	0	0	0	0	0	0	0	0	0	0	0	0	0	0	0	0
**S5-B-PPI**	0	0	0	0	0	0	0	0	0	0	0	0	0	0	0	0
**S5-C-PPI**	0	0	0	0	0	0	0	0	0	0	0	0	0	0	0	0
**S6-A-CTRL**	0	0	0	0	0	0	0	0	0	0	0	0	0	0	0	0
**S6-B-CTRL**	0	0	0	0	0	0	0	0	0	0	0	0	0	0	0	0
**S6-C-CTRL**	0	0	0	0	0	0	0	0	0	0	0	0	0	0	0	0
**S6-A-PPC**	0	0	0	0	0	0	0	0	0	0	0	0	0	0	0	0
**S6-B-PPC**	0	0	0	0	0	0	0	0	0	0	0	0	0	0	0	0
**S6-C-PPC**	0	0	0	0	0	0	0	0	0	0	0	0	0	0	0	0
**S6-A-PPI**	0	0	0	0	0	0	0	0	0	0	0	0	0	0	0	0
**S6-B-PPI**	0	0	0	0	0	0	0	0	0	0	0	0	0.08	0	0	0
**S6-C-PPI**	0	0	0	0	0	0	0	0	0	0	0	0	0	0	0	0

Bacterial species	Novisyntrophococcus below species threshold	Odoribacter below species threshold	Odoribacter splanchnicus	Oliverpabstia below species threshold	Oliverpabstia intestinalis	Oscillibacter acetigenes	Oscillibacter below species threshold	Oscillibacter hominis	Oxalobacter aliiformigenes	Oxalobacter below species threshold	Paenibacillus below species threshold	Palleniella below species threshold	Parabacteroides below species threshold	Parabacteroides distasonis	Parabacteroides faecalis	Parabacteroides faecis
**S1-A-CTRL**	0	0	0.19	0	0	0	0	0	0	0	0	0	0.34	7.12	0	0
**S1-B-CTRL**	0	0	0	0	0	0	0	0	0	0	0	0	0.23	7.04	0	0
**S1-C-CTRL**	0	0	0	0	0	0	0	0	0	0	0	0	0.27	6.09	0	0
**S1-A-PPC**	0	0	0.11	0	0	0	0	0	0	0	0	0	0.08	0.93	0	0
**S1-B-PPC**	0	0	0.13	0	0	0	0	0	0	0	0	0	0.12	0.96	0	0
**S1-C-PPC**	0	0	0.1	0	0	0	0	0	0	0	0	0	0	0.89	0	0
**S1-A-PPI**	0	0	0	0	0	0	0	0	0	0	0	0	0.19	1.74	0	0
**S1-B-PPI**	0	0	0	0	0	0	0	0	0	0	0	0	0.21	2.33	0	0
**S1-C-PPI**	0	0	0	0	0	0	0	0	0	0	0	0	0	2.43	0	0
**S2-A-CTRL**	0	0	0.03	0	0	0	0	0	0	0	0	0	0	0	0	0
**S2-B-CTRL**	0	0	0.02	0	0	0	0	0	0	0	0	0	0	0	0	0
**S2-C-CTRL**	0	0	0.03	0	0	0	0	0	0	0	0	0	0	0	0	0
**S2-A-PPC**	0	0	0.05	0	0	0	0	0	0	0	0	0	0	0	0	0
**S2-B-PPC**	0	0	0.05	0	0	0	0	0	0	0	0	0	0	0	0	0
**S2-C-PPC**	0	0	0.02	0	0	0	0	0	0	0	0	0	0	0	0	0
**S2-A-PPI**	0	0	0.02	0	0	0	0	0	0	0	0	0	0	0	0	0
**S2-B-PPI**	0	0	0.04	0	0	0	0	0	0	0	0	0	0	0	0	0
**S2-C-PPI**	0	0	0.03	0	0	0	0.02	0	0	0	0	0	0	0	0	0
**S3-A-CTRL**	0	0	0	0	0	0	0	0	0	0	0	0	0	0.44	0	0
**S3-B-CTRL**	0	0	0	0	0	0	0	0	0	0	0	0	0	0.22	0	0
**S3-C-CTRL**	0	0	0	0	0	0	0	0	0	0	0	0	0	0.33	0	0
**S3-A-PPC**	0	0	0	0	0	0	0	0	0	0	0	0	0	0.01	0	0
**S3-B-PPC**	0	0	0	0	0	0	0	0	0	0	0	0	0	0.02	0	0
**S3-C-PPC**	0	0	0	0	0	0	0	0	0	0	0	0	0	0.02	0	0
**S3-A-PPI**	0	0	0	0	0	0	0	0	0	0	0	0	0	0.14	0	0
**S3-B-PPI**	0	0	0	0	0	0	0	0	0	0	0	0	0.05	0.13	0	0
**S3-C-PPI**	0	0	0	0	0	0	0.12	0	0	0	0	0	0	0.1	0	0
**S4-A-CTRL**	0	0	0.09	0	0	0	0	0	0	0	0	0	0.28	3.55	0	0
**S4-B-CTRL**	0	0	0.12	0	0	0	0	0	0	0	0	0	0.27	3.64	0	0
**S4-C-CTRL**	0	0	0.15	0	0	0	0	0	0	0	0	0	0.2	2.77	0	0
**S4-A-PPC**	0	0	0.53	0	0	0	0	0	0	0	0	0	0	0.67	0	0
**S4-B-PPC**	0	0	0.43	0	0	0	0	0	0	0	0	0	0	0.66	0	0
**S4-C-PPC**	0	0	0.5	0	0	0	0	0	0	0	0	0	0	0.74	0	0
**S4-A-PPI**	0	0	0.54	0	0	0	0	0	0	0	0	0	0.17	2.41	0	0.06
**S4-B-PPI**	0	0	0.32	0	0	0	0	0	0	0	0	0	0.19	2.33	0	0
**S4-C-PPI**	0	0	0.33	0	0	0	0	0	0	0	0	0	0.16	2.18	0	0
**S5-A-CTRL**	0	0	0	0	0	0	0	0	0	0	0	0	0.18	1.38	0	0
**S5-B-CTRL**	0	0	0	0	0	0	0	0	0	0	0.16	0	0	1.58	0	0
**S5-C-CTRL**	0	0	0	0	0	0	0	0	0	0	0	0	0	1.42	0	0
**S5-A-PPC**	0	0	0	0	0	0	0	0	0	0	0	0	0	0.25	0	0
**S5-B-PPC**	0	0	0	0	0	0	0	0	0	0	0	0	0	0.34	0	0
**S5-C-PPC**	0	0	0	0	0	0	0	0	0	0	0	0	0	0.41	0	0
**S5-A-PPI**	0	0	0	0	0	0	0	0	0	0	0	0	0	1.53	0	0
**S5-B-PPI**	0	0	0	0	0	0	0	0	0	0	0	0	0	1.03	0	0
**S5-C-PPI**	0	0	0	0	0	0	0	0	0	0	0	0	0	0.96	0	0
**S6-A-CTRL**	0	0	0	0	0	0	0	0	0	0	0	0	0	0.29	0	0
**S6-B-CTRL**	0	0	0	0	0	0	0	0	0	0	0	0	0	0.19	0	0
**S6-C-CTRL**	0	0	0	0	0	0	0	0	0	0	0	0	0	0.21	0	0
**S6-A-PPC**	0	0	0	0	0	0	0	0	0	0	0	0	0	0.02	0	0
**S6-B-PPC**	0	0	0	0	0	0	0	0	0	0	0	0	0	0.02	0	0
**S6-C-PPC**	0	0	0	0	0	0	0	0	0	0	0	0	0	0	0	0
**S6-A-PPI**	0	0	0	0	0	0	0.09	0	0	0	0	0	0	0.05	0	0
**S6-B-PPI**	0	0	0	0	0	0	0	0	0	0	0	0	0	0.07	0	0
**S6-C-PPI**	0	0	0	0	0	0	0.06	0	0	0	0	0	0	0.07	0	0

Bacterial species	Parabacteroides goldsteinii	Parabacteroides gordonii	Parabacteroides johnsonii	Parabacteroides leei	Parabacteroides merdae	Parabacteroides multihit species	Paraclostridium bifermentans	Paraclostridium multihit species	Paramuribaculum below species threshold	Paramuribaculum intestinale	Paraprevotella below species threshold	Paraprevotella clara	Parasutterella below species threshold	Parasutterella excrementihominis	Pedobacter below species threshold	Petrimonas below species threshold
**S1-A-CTRL**	0	0	0	0	1.03	0	0	0	0	0	0	0	0	0	0	0
**S1-B-CTRL**	0	0	0	0	0.68	0	0	0	0	0	0	0	0	0	0	0
**S1-C-CTRL**	0	0	0	0	0.75	0	0	0	0	0	0	0	0	0	0	0
**S1-A-PPC**	0	0	0	0	0.24	0	0	0	0	0	0	0	0	0	0	0
**S1-B-PPC**	0	0	0	0	0.3	0	0	0	0	0	0	0	0	0	0	0
**S1-C-PPC**	0	0	0	0	0.24	0	0	0	0	0	0	0	0	0	0	0
**S1-A-PPI**	0	0	0	0	0.15	0	0	0	0	0	0	0	0	0	0	0
**S1-B-PPI**	0	0	0	0	0.29	0	0	0	0	0	0	0	0	0	0	0
**S1-C-PPI**	0	0	0	0	0.24	0	0	0	0	0	0	0	0	0	0	0
**S2-A-CTRL**	0	0	0	0	0	0	0	0	0	0	0	0	0	0.04	0	0
**S2-B-CTRL**	0	0	0	0	0	0	0	0	0	0	0	0	0	0.05	0	0
**S2-C-CTRL**	0	0	0	0	0	0	0	0	0	0	0	0	0	0.05	0	0
**S2-A-PPC**	0	0	0	0	0	0	0	0	0	0	0	0	0	0.07	0	0
**S2-B-PPC**	0	0	0	0	0	0	0	0	0	0	0	0	0	0.08	0	0
**S2-C-PPC**	0	0	0	0	0	0	0	0	0	0	0	0	0	0.07	0	0
**S2-A-PPI**	0	0	0	0	0	0	0	0	0	0	0	0	0	0.06	0	0
**S2-B-PPI**	0	0	0	0	0	0	0	0	0	0	0	0	0	0.08	0	0
**S2-C-PPI**	0	0	0	0	0	0	0	0	0	0	0	0	0	0.05	0	0
**S3-A-CTRL**	0	0	0	0	0.38	0	0	0	0	0	0	0	0	0	0	0
**S3-B-CTRL**	0	0	0	0	0	0	0	0	0	0	0	0	0	0	0	0
**S3-C-CTRL**	0	0	0	0	0.16	0	0	0	0	0	0	0	0	0	0	0
**S3-A-PPC**	0	0	0	0	0	0	0	0	0	0	0	0	0	0	0	0
**S3-B-PPC**	0	0	0	0	0	0	0	0	0	0	0	0	0	0	0	0
**S3-C-PPC**	0	0	0	0	0	0	0	0	0	0	0	0	0	0	0	0
**S3-A-PPI**	0	0	0	0	0	0	0	0	0	0	0	0	0	0	0	0
**S3-B-PPI**	0	0	0	0	0	0	0	0	0	0	0	0	0	0	0	0
**S3-C-PPI**	0	0	0	0	0	0	0	0	0	0	0	0	0	0	0	0
**S4-A-CTRL**	0.05	0	0.12	0	16.05	0.69	0	0	0	0	0	0	0	0	0	0
**S4-B-CTRL**	0.04	0	0.15	0	15.93	0.69	0	0	0	0	0	0	0	0	0	0
**S4-C-CTRL**	0.06	0	0.1	0	12.69	0.54	0	0	0	0	0	0	0	0	0	0
**S4-A-PPC**	0	0	0	0	2.98	0	0	0	0	0	0	0	0	0	0	0
**S4-B-PPC**	0	0	0	0	1.98	0	0	0	0	0	0	0	0	0	0	0
**S4-C-PPC**	0	0	0	0	2.97	0	0.83	0.3	0	0	0	0	0	0	0	0
**S4-A-PPI**	0	0	0.09	0	9.8	0.38	1.14	0.49	0	0	0	0	0	0	0	0
**S4-B-PPI**	0	0	0.07	0	8.67	0.3	0.73	0.39	0	0	0	0	0	0	0	0
**S4-C-PPI**	0	0	0.07	0	7.81	0.31	0.81	0.3	0	0	0	0	0	0	0	0
**S5-A-CTRL**	0	0	0	0	0.81	0.27	0	0	0	0	0	0	0	0	0	0
**S5-B-CTRL**	0	0	0	0	0.89	0	0	0	0	0	0	0	0	0	0	0
**S5-C-CTRL**	0	0	0	0	1.21	0	0	0	0	0	0	0	0	0	0	0
**S5-A-PPC**	0	0	0	0	0	0	0	0	0	0	0	0	0	0.4	0	0
**S5-B-PPC**	0	0	0	0	0	0	0	0	0	0	0	0	0	1.08	0	0
**S5-C-PPC**	0	0	0	0	0	0	0	0	0	0	0	0	0	0.53	0	0
**S5-A-PPI**	0	0	0	0	0.93	0	0	0	0	0	0	0	0	0.82	0	0
**S5-B-PPI**	0	0	0	0	0.37	0	0	0	0	0	0	0	0	0	0	0
**S5-C-PPI**	0	0	0	0	0.57	0	0	0	0	0	0	0	0	0.51	0	0
**S6-A-CTRL**	0	0	0	0	0	0	0	0	0	0	0	0	0	0	0	0
**S6-B-CTRL**	0	0	0	0	0	0	0	0	0	0	0	0	0	0	0	0
**S6-C-CTRL**	0	0	0	0	0.11	0	0	0	0	0	0	0	0	0	0	0
**S6-A-PPC**	0	0	0	0	0	0	0	0	0	0	0	0	0	0	0	0
**S6-B-PPC**	0	0	0	0	0	0	0	0	0	0	0	0	0	0	0	0
**S6-C-PPC**	0	0	0	0	0.02	0	0	0	0	0	0	0	0	0	0	0
**S6-A-PPI**	0	0	0	0	0	0	0	0	0	0	0	0	0	0	0	0
**S6-B-PPI**	0	0	0	0	0	0	0	0	0	0	0	0	0	0	0	0
**S6-C-PPI**	0	0	0	0	0	0	0	0	0	0	0	0	0	0	0	0

**Bacterial species**	Phascolarctobacterium below species threshold	Phascolarctobacterium faecium	Phocaeicola barnesiae	Phocaeicola below species threshold	Phocaeicola coprocola	Phocaeicola dorei	Phocaeicola faecalis	Phocaeicola faecicola	Phocaeicola faecium	Phocaeicola fibrisolvens	Phocaeicola massiliensis	Phocaeicola multihit species	Phocaeicola plebeius	Phocaeicola salanitronis	Phocaeicola sartorii	Phocaeicola vulgatus
**S1-A-CTRL**	0	3.18	0	0.31	0	0.17	0	0	0	0	5.47	7.8	0	0	0	2.87
**S1-B-CTRL**	0	4.34	0	0	0	0	0	0	0	0	2.95	5.83	0	0	0	2
**S1-C-CTRL**	0	2.16	0	0.43	0	0.27	0	0	0	0	5.37	8.26	0	0	0	3.15
**S1-A-PPC**	0	0.78	0	0.1	0	0.14	0	0	0	0	1.74	3.28	0	0	0	1.03
**S1-B-PPC**	0	0.91	0	0.15	0	0	0	0	0	0	1.96	3.48	0	0	0	1.24
**S1-C-PPC**	0	0.68	0	0.11	0	0.11	0	0	0	0	1.43	2.82	0	0	0	0.99
**S1-A-PPI**	0	0.8	0	0.23	0	0.21	0	0	0	0	6.22	5.2	0	0	0	2.36
**S1-B-PPI**	0	1.11	0	0.27	0	0	0	0	0	0	5.22	4.8	0	0	0	1.85
**S1-C-PPI**	0	0.55	0	0.2	0	0.1	0	0	0	0	8.36	4.37	0	0	0	1.69
**S2-A-CTRL**	0	0	0	0	0	0.01	0	0	0	0	0	0.36	0	0	0	0.09
**S2-B-CTRL**	0	0	0	0	0	0	0	0	0	0	0	0.3	0	0	0	0.1
**S2-C-CTRL**	0	0	0	0	0	0	0	0	0	0	0	0.29	0	0	0	0.1
**S2-A-PPC**	0	0	0	0	0	0.01	0	0	0	0	0	0.33	0	0	0	0.1
**S2-B-PPC**	0	0	0	0	0	0	0	0	0	0	0	0.29	0	0	0	0.1
**S2-C-PPC**	0	0	0	0	0	0.01	0	0	0	0	0	0.24	0	0	0	0.08
**S2-A-PPI**	0	0	0	0	0	0	0	0	0	0	0	0.35	0	0	0	0.12
**S2-B-PPI**	0	0	0	0	0	0.01	0	0	0	0	0	0.4	0	0	0	0.12
**S2-C-PPI**	0	0	0	0	0	0	0	0	0	0	0	0.42	0	0	0	0.13
**S3-A-CTRL**	0	0	0	0.35	0	0	0	0	0	0	0.65	1.71	0	0	0	0.31
**S3-B-CTRL**	0	0	0	0.68	0	0	0	0	0	0	0.64	0.94	0	0	0	0.26
**S3-C-CTRL**	0	0	0	0.58	0	0	0	0	0	0	0.62	1.12	0	0	0	0.21
**S3-A-PPC**	0	0	0	0.12	0	0	0	0	0	0	0.04	0.06	0	0	0	0
**S3-B-PPC**	0	0	0	0.14	0	0	0	0	0	0	0.03	0.06	0	0	0	0
**S3-C-PPC**	0	0	0	0.11	0	0	0	0	0	0	0.04	0.05	0	0	0	0.01
**S3-A-PPI**	0	0	0	0.2	0	0	0	0	0	0	0.78	0.97	0	0	0	0.26
**S3-B-PPI**	0	0	0	0.21	0	0	0	0	0	0	0.31	0.29	0	0	0	0
**S3-C-PPI**	0	0	0	0.2	0	0	0	0	0	0	0.39	0.5	0	0	0	0.18
**S4-A-CTRL**	0	0	0	0.23	0	4.79	0	0	0	0	0.05	9.29	0	0	0.07	1.7
**S4-B-CTRL**	0	0	0	0.25	0	4.75	0	0	0	0	0.06	9.17	0	0	0.12	1.8
**S4-C-CTRL**	0	0	0	0.31	0	4.23	0	0	0	0	0.07	11.49	0	0	0.06	2.37
**S4-A-PPC**	0	0	0	0	0	1.17	0	0	0	0	0	3.38	0	0	0	0.73
**S4-B-PPC**	0	0	0	0	0	1.28	0	0	0	0	0	4.13	0	0	0	0.84
**S4-C-PPC**	0	0	0	0	0	1.53	0	0	0	0	0	5.95	0	0	0	1.07
**S4-A-PPI**	0	0	0	0.27	0	5.36	0	0	0	0	0	13.47	0	0	0.1	2.76
**S4-B-PPI**	0	0	0	0.29	0	5.66	0	0	0	0	0.04	14.09	0	0	0.08	2.9
**S4-C-PPI**	0	0	0	0.18	0	3.61	0	0	0	0	0.04	11.63	0	0	0.1	2.16
**S5-A-CTRL**	0	0	0	0	0	1.47	0	0	0	0	0.29	16.82	0	0	0.18	5.12
**S5-B-CTRL**	0	0	0	0	0	1.48	0	0	0	0	0	16.12	0	0	0	4.37
**S5-C-CTRL**	0	0	0	0	0	1.22	0	0	0	0	0	15.83	0	0	0	4.26
**S5-A-PPC**	0	0	0	0	0	0	0	0	0	0	0	0.77	0	0	0	0.37
**S5-B-PPC**	0	0	0	0	0	0	0	0	0	0	0	0.74	0	0	0	0.3
**S5-C-PPC**	0	0	0	0	0	0.22	0	0	0	0	0	0.86	0	0	0	0.29
**S5-A-PPI**	0	0	0	0	0	1.33	0	0	0	0	0	5.91	0	0	0	1.37
**S5-B-PPI**	0	0	0	0	0	1.31	0	0	0	0	0	5.39	0	0	0	1.66
**S5-C-PPI**	0	0	0	0.16	0	0.86	0	0	0	0	0	7.82	0	0	0	2.14
**S6-A-CTRL**	0	0	0	0	0	0	0	0	0	0	0	0.72	0	0	0	0.34
**S6-B-CTRL**	0	0	0	0	0	0	0	0	0	0	0	0.59	0	0	0	0.26
**S6-C-CTRL**	0	0	0	0	0	0	0	0	0	0	0	0.67	0	0	0	0.27
**S6-A-PPC**	0	0	0	0	0	0	0	0	0	0	0.03	0.04	0	0	0	0.03
**S6-B-PPC**	0	0	0	0	0	0	0	0	0	0	0.03	0.02	0	0	0	0
**S6-C-PPC**	0	0	0	0	0	0	0	0	0	0	0.03	0.03	0	0	0	0.02
**S6-A-PPI**	0	0	0	0	0	0	0	0	0	0	0.1	0.23	0	0	0	0.07
**S6-B-PPI**	0	0	0	0	0	0	0	0	0	0	0.11	0.19	0	0	0	0.08
**S6-C-PPI**	0	0	0	0	0	0	0	0	0	0	0.15	0.21	0	0	0	0.07

Bacterial species	Photobacterium below species threshold	Pilosibacter below species threshold	Pilosibacter fragilis	Pontibacter below species threshold	Porcipelethomonas ammonilytica	Porcipelethomonas below species threshold	Porphyromonas below species threshold	Prevotella below species threshold	Prevotella communis	Prevotella corporis	Prevotella disiens	Prevotella lacticifex	Prevotella marseillensis	Prevotella merdae	Prevotella multihit species	Prevotella pectinovora
**S1-A-CTRL**	0	0	0	0	0	0	0	0	0	0	0	0	0	0	0	0
**S1-B-CTRL**	0	0	0	0	0	0	0	0	0	0	0	0	0	0	0	0
**S1-C-CTRL**	0	0	0	0	0	0	0	0	0	0	0	0	0	0	0	0
**S1-A-PPC**	0.1	0.12	0	0	0	0	0	0	0	0	0	0	0	0	0	0
**S1-B-PPC**	0	0.14	0	0	0	0	0	0	0	0	0	0	0	0	0	0
**S1-C-PPC**	0	0	0	0	0	0	0	0	0	0	0	0	0	0	0	0
**S1-A-PPI**	0	0	0	0	0	0	0	0	0	0	0	0	0	0	0	0
**S1-B-PPI**	0	0	0	0	0	0	0	0	0	0	0	0	0	0	0	0
**S1-C-PPI**	0	0	0	0	0	0	0	0	0	0	0	0	0	0	0	0
**S2-A-CTRL**	0	0.03	0.02	0	0	0	0	0	0	0	0	0	0	0	0	0
**S2-B-CTRL**	0	0.02	0.01	0	0	0	0	0	0	0	0	0	0	0	0	0
**S2-C-CTRL**	0	0.01	0	0	0	0	0	0	0	0	0	0	0	0	0	0
**S2-A-PPC**	0	0.04	0.04	0	0	0	0	0	0	0	0	0	0	0	0	0
**S2-B-PPC**	0	0.02	0.02	0	0	0	0	0	0	0	0	0	0	0	0	0
**S2-C-PPC**	0	0.04	0.03	0	0	0	0	0	0	0	0	0	0	0	0	0
**S2-A-PPI**	0	0.05	0	0	0	0	0	0	0	0	0	0	0	0	0	0
**S2-B-PPI**	0	0.03	0	0	0	0	0	0	0	0	0	0	0	0	0	0
**S2-C-PPI**	0	0.03	0.03	0	0	0	0	0	0	0	0	0	0	0	0	0
**S3-A-CTRL**	0	0	0	0	0	0	0	0.48	0	0	0	0	0	0	0	0
**S3-B-CTRL**	0	0	0	0	0	0	0	0.73	0	0	0	0	0	0	0	0
**S3-C-CTRL**	0	0	0	0	0	0	0	0.91	0	0	0	0	0	0	0	0
**S3-A-PPC**	0	0	0	0	0	0	0	0.16	0	0	0	0	0	0	0	0
**S3-B-PPC**	0	0	0	0	0	0	0	0.15	0	0	0	0	0	0	0	0
**S3-C-PPC**	0	0	0	0	0	0	0	0.08	0	0	0	0	0	0	0	0
**S3-A-PPI**	0	0	0	0	0	0	0	0.5	0	0	0	0	0	0	0	0
**S3-B-PPI**	0	0	0	0	0	0	0	0.44	0	0	0	0	0	0	0	0
**S3-C-PPI**	0	0	0	0	0	0	0	0.45	0	0	0	0	0	0	0	0
**S4-A-CTRL**	0	0	0	0	0	0	0	0	0	0	0	0	0	0	0	0
**S4-B-CTRL**	0	0	0	0	0	0	0	0	0	0	0	0	0	0	0	0
**S4-C-CTRL**	0	0	0	0	0	0	0	0	0	0	0	0	0	0	0	0
**S4-A-PPC**	0	0	0	0	0	0	0	0	0	0	0	0	0	0	0	0
**S4-B-PPC**	0	0	0	0	0	0	0	0	0	0	0	0	0	0	0	0
**S4-C-PPC**	0	0	0	0	0	0	0	0	0	0	0	0	0	0	0	0
**S4-A-PPI**	0	0	0	0	0	0	0	0	0	0	0	0	0	0	0	0
**S4-B-PPI**	0	0	0	0	0	0	0	0	0	0	0	0	0	0	0	0
**S4-C-PPI**	0	0	0	0	0	0	0	0	0	0	0	0	0	0	0	0
**S5-A-CTRL**	0	0	0	0	0	0	0	0.93	0	0	0	0	0	0	0	0
**S5-B-CTRL**	0	0	0	0	0	0	0	0	0	0	0	0	0	0	0	0
**S5-C-CTRL**	0	0	0	0	0	0	0	0	0	0	0	0	0	0	0	0
**S5-A-PPC**	0	0	0	0	0	0	0	0	0	0	0	0	0	0	0	0
**S5-B-PPC**	0	0	0	0	0	0	0	0	0	0	0	0	0	0	0	0
**S5-C-PPC**	0	0	0	0	0	0	0	0	0	0	0	0	0	0	0	0
**S5-A-PPI**	0	0	0	0	0	0	0	0	0	0	0	0	0	0	0	0
**S5-B-PPI**	0	0	0	0	0	0	0	0.22	0	0	0	0	0	0	0	0
**S5-C-PPI**	0	0	0	0	0	0	0	0	0	0	0	0	0	0	0	0
**S6-A-CTRL**	0	0	0	0	0	0	0	0	0	0	0	0	0	0	0	0
**S6-B-CTRL**	0	0	0	0	0	0	0	0	0	0	0	0	0	0	0	0
**S6-C-CTRL**	0	0	0	0	0	0	0	0	0	0	0	0	0	0	0	0
**S6-A-PPC**	0	0	0	0	0	0	0	0.03	0	0	0	0	0	0	0	0
**S6-B-PPC**	0	0	0	0	0	0	0	0.03	0	0	0	0	0	0	0	0
**S6-C-PPC**	0	0	0	0	0	0	0	0.05	0	0	0	0	0	0	0	0
**S6-A-PPI**	0	0	0	0	0	0	0	0	0	0	0	0	0	0	0	0
**S6-B-PPI**	0	0	0	0	0	0	0	0.07	0	0	0	0	0	0	0	0
**S6-C-PPI**	0	0	0	0	0	0	0	0.11	0	0	0	0	0	0	0	0

**Bacterial species**	Prevotellamassilia below species threshold	Prevotellamassilia timonensis	Proteus mirabilis	Pseudoduganella below species threshold	Pseudoflavonifractor below species threshold	Pseudoflavonifractor capillosus	Pseudomonas below species threshold	Pseudoprevotella below species threshold	Pseudoprevotella muciniphila	Pseudoruminococcus below species threshold	Pseudoruminococcus massiliensis	Pusillibacter below species threshold	Pusillibacter faecalis	Raoultella below species threshold	Raoultella planticola	Rikenella below species threshold
**S1-A-CTRL**	0	0	0	0	0	0	0	0	0	0	0	0	0	0	0	0
**S1-B-CTRL**	0	0	0	0	0	0	0	0	0	0	0	0	0	0	0	0
**S1-C-CTRL**	0	0	0	0	0	0	0	0	0	0	0	0	0	0	0	0
**S1-A-PPC**	0	0	0.18	0	0	0	0	0	0	0	0	0	0	0	0	0
**S1-B-PPC**	0	0	0.12	0	0	0	0	0	0	0	0	0	0	0	0	0
**S1-C-PPC**	0	0	0.09	0	0	0	0	0	0	0	0	0	0	0	0	0
**S1-A-PPI**	0	0	0	0	0	0	0	0	0	0	0	0	0	0	0	0
**S1-B-PPI**	0	0	0	0	0	0	0	0	0	0	0	0	0	0	0	0
**S1-C-PPI**	0	0	0	0	0	0	0	0	0	0	0	0	0	0	0	0
**S2-A-CTRL**	0	0	0	0	0.02	0	0	0	0	0	0	0	0	0.01	0	0
**S2-B-CTRL**	0	0	0	0	0	0	0	0	0	0	0	0	0	0.01	0	0
**S2-C-CTRL**	0	0	0	0	0.01	0.01	0	0	0	0	0	0	0	0.01	0	0
**S2-A-PPC**	0	0	0	0	0.06	0	0	0	0	0	0	0	0	0	0	0
**S2-B-PPC**	0	0	0	0	0.02	0	0	0	0	0	0	0	0	0	0	0
**S2-C-PPC**	0	0	0	0	0.02	0	0	0	0	0	0	0	0	0	0	0
**S2-A-PPI**	0	0	0	0	0	0	0	0	0	0	0	0	0	0.02	0	0
**S2-B-PPI**	0	0	0	0	0.04	0	0	0	0	0	0	0	0	0.01	0	0
**S2-C-PPI**	0	0	0	0	0.03	0	0	0	0	0	0	0	0	0.01	0.01	0
**S3-A-CTRL**	0	0	0	0	0	0	0	0	0	0	0	0	0	0	0	0
**S3-B-CTRL**	0	0	0	0	0	0	0	0	0	0	0	0	0	0	0	0
**S3-C-CTRL**	0	0	0	0	0	0	0	0	0	0	0	0	0	0	0	0
**S3-A-PPC**	0	0	0	0	0.03	0	0	0	0	0	0	0	0	0	0	0
**S3-B-PPC**	0	0	0	0	0	0	0	0	0	0	0	0	0	0	0	0
**S3-C-PPC**	0	0	0	0	0	0	0	0	0	0	0	0	0	0	0	0
**S3-A-PPI**	0	0	0	0	0	0	0	0	0	0	0	0	0	0	0	0
**S3-B-PPI**	0	0	0	0	0	0	0	0	0	0	0	0	0	0	0	0
**S3-C-PPI**	0	0	0	0	0	0	0	0	0	0	0	0	0	0	0	0
**S4-A-CTRL**	0	0	0	0	0	0	0	0	0	0	0	0	0	0	0	0
**S4-B-CTRL**	0	0	0	0	0	0	0	0	0	0	0	0	0	0	0	0
**S4-C-CTRL**	0	0	0	0	0	0	0	0	0	0	0	0	0	0	0	0
**S4-A-PPC**	0	0	0	0	0	0	0	0	0	0	0	0	0	0	0	0
**S4-B-PPC**	0	0	0	0	0	0	0	0	0	0	0	0	0	0	0	0
**S4-C-PPC**	0	0	0	0	0	0	0	0	0	0	0	0	0	0	0	0
**S4-A-PPI**	0	0	0	0	0	0	0	0	0	0	0	0	0	0	0	0
**S4-B-PPI**	0	0	0	0	0	0	0	0	0	0	0	0	0	0	0	0
**S4-C-PPI**	0	0	0	0	0	0	0	0	0	0	0	0	0	0	0	0
**S5-A-CTRL**	0	0	0	0	0	0	0	0	0	0	0	0	0	0	0	0
**S5-B-CTRL**	0	0	0	0	0	0	0	0	0	0	0	0	0	0	0	0
**S5-C-CTRL**	0	0	0	0	0	0	0	0	0	0	0	0	0	0	0	0
**S5-A-PPC**	0	0	0	0	0	0	0	0	0	0	0	0	0	0	0	0
**S5-B-PPC**	0	0	0	0	0	0	0	0	0	0	0	0	0	0	0	0
**S5-C-PPC**	0	0	0	0	0	0	0	0	0	0	0	0	0	0	0	0
**S5-A-PPI**	0	0	0	0	0	0	0	0	0	0	0	0	0	0	0	0
**S5-B-PPI**	0	0	0	0	0	0	0	0	0	0	0	0	0	0	0	0
**S5-C-PPI**	0	0	0	0	0	0	0	0	0	0	0	0	0	0	0	0
**S6-A-CTRL**	0	0	0	0	0.17	0	0	0	0	0	0	0	0	0	0	0
**S6-B-CTRL**	0	0	0	0	0	0	0	0	0	0	0	0	0	0	0	0
**S6-C-CTRL**	0	0	0	0	0	0	0	0	0	0	0	0	0	0	0	0
**S6-A-PPC**	0	0	0	0	0	0	0	0	0	0	0	0	0	0	0	0
**S6-B-PPC**	0	0	0	0	0.04	0	0	0	0	0	0	0	0	0	0	0
**S6-C-PPC**	0.03	0	0	0	0.03	0	0	0	0	0	0	0	0	0	0	0
**S6-A-PPI**	0.06	0	0	0	0	0	0	0	0	0	0	0	0	0	0	0
**S6-B-PPI**	0	0	0	0	0.06	0	0	0	0	0	0	0	0	0	0	0
**S6-C-PPI**	0.07	0	0	0	0.06	0	0	0	0	0	0	0	0	0	0	0

**Bacterial species**	Rikenella microfusus	Robinsoniella below species threshold	Roseburia amylophila	Roseburia below species threshold	Roseburia faecis	Roseburia hominis	Roseburia intestinalis	Roseburia inulinivorans	Roseburia lenta	Roseburia multihit species	Roseburia rectibacter	Roseburia yibonii	Ruminococcoides below species threshold	Ruminococcoides intestinale	Ruminococcoides intestinihominis	Ruminococcus below species threshold
**S1-A-CTRL**	0	0	0	0	0	0	0	0	0	0	0	0	0	0	0	0
**S1-B-CTRL**	0	0	0	0	0	0	0	0	0	0	0	0	0	0	0	0
**S1-C-CTRL**	0	0	0	0	0	0	0	0	0	0	0	0	0	0	0	0
**S1-A-PPC**	0	0	0	0	0	0	0	0	0	0	0	0	0	0	0	0
**S1-B-PPC**	0	0	0	0	0	0	0	0	0	0	0	0	0	0	0	0
**S1-C-PPC**	0	0	0	0	0	0	0	0	0	0	0	0	0	0	0	0
**S1-A-PPI**	0	0	0	0	0	0	0	0	0	0	0	0	0	0	0	0
**S1-B-PPI**	0	0	0	0	0	0	0	0	0	0	0	0	0	0	0	0
**S1-C-PPI**	0	0	0	0	0	0	0	0	0	0	0	0	0	0	0	0
**S2-A-CTRL**	0	0	0	0.02	0.32	0	0	0.02	0	0.02	0	0	0	0.58	0	0.04
**S2-B-CTRL**	0	0	0	0.02	0.31	0	0	0.05	0	0.02	0	0	0	0.35	0	0.03
**S2-C-CTRL**	0	0	0	0	0.35	0	0	0.03	0	0	0	0	0	0.49	0	0.07
**S2-A-PPC**	0	0	0	0.1	1.48	0.05	0.02	0.25	0	0.09	0	0	0.06	2	0	0.09
**S2-B-PPC**	0	0	0	0.06	1.16	0.05	0	0.25	0	0.07	0	0	0.09	2.16	0.02	0.07
**S2-C-PPC**	0	0	0	0.06	1.17	0.07	0.01	0.28	0	0.08	0	0	0.05	1.28	0	0.06
**S2-A-PPI**	0	0	0	0.07	1.13	0.05	0	0.18	0	0.07	0	0	0.04	1.03	0	0.06
**S2-B-PPI**	0	0	0	0.05	1.31	0.04	0	0.25	0	0.07	0	0	0	0.66	0	0.07
**S2-C-PPI**	0	0	0	0.06	1.15	0.04	0	0.18	0	0.07	0	0.02	0.04	0.99	0	0.1
**S3-A-CTRL**	0	0	0	0	0.94	0	0	0	0	0	0	0	0	0	0	0
**S3-B-CTRL**	0	0	0	0	0.73	0	0	0	0	0	0	0	0	0	0	0
**S3-C-CTRL**	0	0	0	0	1.01	0	0	0	0	0	0	0	0	0	0	0
**S3-A-PPC**	0	0	0	0.02	0.3	0.03	0	0	0	0	0	0	0	0.27	0	0.11
**S3-B-PPC**	0	0	0	0	0.23	0	0	0	0	0	0	0	0	0.19	0	0.11
**S3-C-PPC**	0	0	0	0	0.24	0.02	0	0	0	0	0	0	0	0.23	0	0.12
**S3-A-PPI**	0	0	0	0	0.83	0	0	0	0	0	0	0	0	0.23	0	0.32
**S3-B-PPI**	0	0	0	0	0.74	0	0	0	0	0	0	0	0	0.25	0	0.41
**S3-C-PPI**	0	0	0	0	0.79	0	0	0	0	0	0	0	0	0	0	0.3
**S4-A-CTRL**	0	0	0	0	0	0	0	0	0	0	0	0	0	0	0	0
**S4-B-CTRL**	0	0	0	0	0	0	0	0	0	0	0	0	0	0.09	0	0
**S4-C-CTRL**	0	0	0	0	0.04	0	0	0	0	0	0	0	0	0	0	0
**S4-A-PPC**	0	0	0	0	3.05	0	0	0	0	0	0	0	0	0.44	0	0.7
**S4-B-PPC**	0	0	0	0.3	2.36	0	0	0.27	0	0	0	0	0	0	0	0.53
**S4-C-PPC**	0	0	0	0	1.72	0	0	0	0	0	0	0	0	0.36	0	0.44
**S4-A-PPI**	0	0	0	0	0.19	0	0	0.05	0	0	0	0	0	0.19	0	0.17
**S4-B-PPI**	0	0	0	0	0.24	0	0	0	0	0	0	0	0	0	0	0.09
**S4-C-PPI**	0	0	0	0	0.19	0	0	0	0	0	0	0	0	0.07	0	0.06
**S5-A-CTRL**	0	0	0	0	0	0	0	0	0	0	0	0	0	0	0	0.28
**S5-B-CTRL**	0	0	0	0	0	0	0	0	0	0	0	0	0	0	0	0
**S5-C-CTRL**	0	0	0	0	0	0	0	0	0	0	0	0	0	0	0	0
**S5-A-PPC**	0	0	0	0	0	0	0	0	0	0	0	0	0	0	0	1.04
**S5-B-PPC**	0	0	0	0	0	0	0	0	0	0	0	0	0	0	0	0.83
**S5-C-PPC**	0	0	0	0	0	0	0	0	0	0	0	0	0	0	0	0.92
**S5-A-PPI**	0	0	0	0	0	0	0	0	0	0	0	0	0	0	0	0.33
**S5-B-PPI**	0	0	0	0	0	0	0	0	0	0	0	0	0	0	0	0.65
**S5-C-PPI**	0	0	0	0	0	0	0	0	0	0	0	0	0	0	0	0.37
**S6-A-CTRL**	0	0	0	0	3.28	0	0	0	0	0	0	0	0	1.49	0	0
**S6-B-CTRL**	0	0	0	0	3.07	0	0	0	0	0	0	0	0	1.17	0	0
**S6-C-CTRL**	0	0	0	0	3.49	0	0	0	0	0	0	0	0	1.21	0	0
**S6-A-PPC**	0	0	0.09	0.03	1.38	0	0.02	0.02	0	0.04	0	0	0.04	0.46	0	0.06
**S6-B-PPC**	0	0	0.03	0.03	1.4	0	0	0	0	0.04	0	0	0	0.4	0	0.04
**S6-C-PPC**	0	0	0.08	0.04	1.63	0	0	0.03	0	0.04	0	0	0	0.67	0	0.05
**S6-A-PPI**	0	0	0.17	0.18	5.51	0	0.05	0	0	0.16	0	0	0	1.37	0	0
**S6-B-PPI**	0	0	0.17	0.13	5.44	0	0	0.09	0	0.17	0	0	0	1.16	0	0.15
**S6-C-PPI**	0	0	0.14	0.13	5.43	0	0.08	0.12	0	0.18	0	0	0.08	1.24	0	0.1

Bacterial species	[Ruminococcus] below species threshold	Ruminococcus bromii	Ruminococcus callidus	Ruminococcus intestinalis	Ruminococcus lactaris	Ruminococcus torques	Ruthenibacterium below species threshold	Ruthenibacterium lactatiformans	Salmonella below species threshold	Salmonella enterica	Schaedlerella below species threshold	Segatella albensis	Segatella below species threshold	Segatella bryantii	Segatella copri	Segatella hominis
**S1-A-CTRL**	0	0	0	0	0	0.6	0	0	0	0	0	0	0	0	0	0
**S1-B-CTRL**	0	0	0	0	0	0.48	0	0	0	0	0	0	0	0	0	0
**S1-C-CTRL**	0	0	0	0	0	1.15	0	0	0	0	0	0	0	0	0	0
**S1-A-PPC**	0	0	0	0	0	1.4	0	0	0	0	0	0	0	0	0	0
**S1-B-PPC**	0	0	0	0	0	1.46	0	0	0.14	0	0	0	0	0	0	0
**S1-C-PPC**	0	0	0	0	0	1.42	0	0	0	0	0	0	0	0	0	0
**S1-A-PPI**	0	0	0	0	0	0.85	0	0	0	0	0	0	0	0	0	0
**S1-B-PPI**	0	0	0	0	0	1.69	0	0	0	0	0	0	0	0	0	0
**S1-C-PPI**	0	0	0	0	0	1.11	0	0	0	0	0	0	0	0	0	0
**S2-A-CTRL**	0	0.06	0	0	0	0	0	0.01	0	0.01	0	0	0	0	0	0
**S2-B-CTRL**	0	0.03	0	0	0	0	0	0.02	0	0.02	0	0	0	0	0	0
**S2-C-CTRL**	0	0.04	0	0	0	0	0	0.01	0.01	0.01	0	0	0	0	0	0
**S2-A-PPC**	0	0.16	0	0	0	0	0	0.02	0	0	0	0	0	0	0	0
**S2-B-PPC**	0	0.16	0	0	0	0	0	0.02	0	0.02	0	0	0	0	0	0
**S2-C-PPC**	0	0.1	0	0	0	0	0	0	0	0	0	0	0	0	0	0
**S2-A-PPI**	0	0.05	0	0	0	0	0	0	0	0	0	0	0	0	0	0
**S2-B-PPI**	0	0.06	0	0	0	0	0.02	0.03	0	0.02	0	0	0	0	0	0
**S2-C-PPI**	0	0.1	0	0	0	0	0.02	0.02	0	0.01	0	0	0	0	0	0
**S3-A-CTRL**	0	0	0	0	0	0	0	0	0	0	0	0	0.19	0	0	0
**S3-B-CTRL**	0	0	0	0	0	0.39	0	0	0	0	0	0	0.22	0	0	0
**S3-C-CTRL**	0	0	0	0	0	0.32	0	0	0	0	0	0	0.28	0	0	0
**S3-A-PPC**	0	0.03	0.08	0	0	0.15	0	0	0	0	0	0	0.05	0	0	0
**S3-B-PPC**	0	0	0.05	0	0	0.14	0	0	0	0	0	0	0.05	0	0	0
**S3-C-PPC**	0	0	0.05	0	0	0.1	0	0	0	0	0	0	0.04	0	0	0
**S3-A-PPI**	0	0	0.2	0	0	0.3	0	0	0	0	0	0	0.13	0	0	0
**S3-B-PPI**	0	0	0.12	0	0	0.47	0	0	0	0.07	0	0	0.2	0	0	0
**S3-C-PPI**	0	0	0	0	0	0.37	0	0	0	0	0	0	0	0	0	0
**S4-A-CTRL**	0	0	0	0	0.14	0.18	0	0	0	0.08	0	0	0	0	0	0
**S4-B-CTRL**	0	0	0	0	0.17	0.17	0	0.06	0	0.07	0	0	0	0	0	0
**S4-C-CTRL**	0	0	0	0	0.3	0.26	0	0.04	0	0.06	0	0	0	0	0	0
**S4-A-PPC**	0	0	0	0	2.39	2.79	0	0.25	0	0.41	0	0	0	0	0	0
**S4-B-PPC**	0	0	0	0	2.7	3.05	0	0.37	0	0.58	0	0	0	0	0	0
**S4-C-PPC**	0	0	0	0	2.57	3.06	0.21	0	0	0.4	0	0	0	0	0	0
**S4-A-PPI**	0	0	0	0	0.67	0.74	0	0.07	0	0.09	0	0	0	0	0	0
**S4-B-PPI**	0	0	0	0	0.4	0.47	0.04	0	0	0.08	0	0	0	0	0	0
**S4-C-PPI**	0	0	0	0	0.55	0.67	0	0.05	0	0.06	0	0	0	0	0	0
**S5-A-CTRL**	0	0	0	0	0	0	0	0	0	0	0	0	0	0	0	0
**S5-B-CTRL**	0	0	0	0	0	0	0	0	0	0	0	0	0	0	0	0
**S5-C-CTRL**	0	0	0	0	0	0	0	0	0	0	0	0	0	0	0	0
**S5-A-PPC**	0	0	0	0	0	0	0	0	0	0	0	0	0	0	0	0
**S5-B-PPC**	0	0	0	0	0	0	0	0	0	0	0	0	0	0	0	0
**S5-C-PPC**	0	0	0	0	0	0	0	0	0	0	0	0	0	0	0	0
**S5-A-PPI**	0	0	0	0	0	0	0	0	0	0.13	0	0	0	0	0	0
**S5-B-PPI**	0	0	0	0	0	0	0	0	0	0	0	0	0	0	0	0
**S5-C-PPI**	0	0	0	0	0	0.17	0	0	0	0	0	0	0	0	0	0
**S6-A-CTRL**	0	0	0	0	0	0	0	0	0.64	0.27	0	0	0	0	0.53	0
**S6-B-CTRL**	0	0	0	0	0	0	0	0	0.35	0.17	0	0	0	0	0.56	0
**S6-C-CTRL**	0	0	0	0	0	0	0	0	0.32	0.3	0	0	0.23	0	0.82	0
**S6-A-PPC**	0	0.04	0	0	0	0	0	0	0.09	0.03	0	0	0.07	0	0.31	0
**S6-B-PPC**	0	0.06	0	0	0	0	0	0	0.14	0	0	0	0.06	0	0.27	0
**S6-C-PPC**	0	0.05	0	0	0	0	0	0	0.17	0	0	0	0.11	0	0.36	0
**S6-A-PPI**	0	0.09	0	0	0	0	0	0	0.19	0.08	0	0	0.07	0	0.64	0
**S6-B-PPI**	0	0	0	0	0	0	0	0	0.23	0.09	0	0	0.14	0	0.66	0
**S6-C-PPI**	0	0.15	0	0	0	0	0	0	0.19	0.06	0	0	0.22	0	0.54	0

**Bacterial species**	Segatella multihit species	Selenomonas below species threshold	Selenomonas bovis	Selenomonas montiformis	Sellimonas below species threshold	Sellimonas intestinalis	Serratia below species threshold	Serratia grimesii	Serratia marcescens	Serratia multihit species	Shigella below species threshold	Shigella boydii	Shigella dysenteriae	Shigella flexneri	Shigella multihit species	Shigella sonnei
**S1-A-CTRL**	0	0	0	0	0	0	0	0.26	0	0	0	0	0.28	0	0.28	0.31
**S1-B-CTRL**	0	0	0	0	0	0	0	0.38	0	0	0	0	0.29	0	0.31	0.25
**S1-C-CTRL**	0	0	0	0	0	0	0	0.23	0	0	0	0	0.14	0	0.33	0.25
**S1-A-PPC**	0	0	0	0	0	0	0	0.16	0	0	0	0.09	0.11	0.08	0.23	0.19
**S1-B-PPC**	0	0	0	0	0	0	0	0.11	0	0	0	0	0.1	0.1	0.14	0.22
**S1-C-PPC**	0	0	0	0	0	0	0	0.11	0	0	0	0	0.11	0	0.19	0.1
**S1-A-PPI**	0	0	0	0	0	0	0.12	0.18	0	0	0	0	0.28	0	0.2	0.27
**S1-B-PPI**	0	0	0	0	0	0	0	0.24	0	0	0	0	0.28	0	0.22	0.28
**S1-C-PPI**	0	0	0	0	0	0	0	0.16	0	0	0	0	0.1	0	0.14	0.21
**S2-A-CTRL**	0	0	0	0	0	0	0	0	0	0.06	0	0	0	0.01	0	0
**S2-B-CTRL**	0	0	0	0	0	0	0	0	0	0.11	0	0	0	0.01	0	0
**S2-C-CTRL**	0	0	0	0	0	0	0	0	0	0.09	0	0	0	0	0	0
**S2-A-PPC**	0	0	0	0	0	0	0	0	0	0.08	0	0	0	0	0	0
**S2-B-PPC**	0	0	0	0	0	0	0	0	0	0.08	0	0	0.02	0	0	0
**S2-C-PPC**	0	0	0	0	0	0	0	0	0	0.07	0	0	0	0	0	0
**S2-A-PPI**	0	0	0	0	0	0	0	0	0	0.1	0	0	0.01	0	0	0
**S2-B-PPI**	0	0	0	0	0	0	0	0	0	0.09	0	0	0	0	0	0
**S2-C-PPI**	0	0	0	0	0	0	0	0	0	0.09	0	0	0	0	0	0
**S3-A-CTRL**	0	0	0	0	0	0	0	0	0.11	0.13	0	0	0.13	6.85	0.95	0.14
**S3-B-CTRL**	0	0	0	0	0	0	0	0	0	0.28	0.15	0	0.28	9.83	1.17	0.2
**S3-C-CTRL**	0	0	0	0	0	0	0	0	0	0.2	0	0	0.15	8.91	1.12	0.18
**S3-A-PPC**	0	0	0	0	0	0	0	0	0.01	0.01	0	0	0.02	0.44	0.06	0
**S3-B-PPC**	0	0	0	0	0	0	0	0	0.01	0	0	0.02	0.01	0.44	0.05	0
**S3-C-PPC**	0	0	0	0	0	0	0	0	0	0	0	0	0.01	0.45	0.07	0
**S3-A-PPI**	0	0	0	0	0	0	0	0	0.09	0.08	0	0	0.08	4.12	0.41	0
**S3-B-PPI**	0	0	0	0	0	0	0	0	0	0.05	0	0	0.05	3.09	0.33	0.11
**S3-C-PPI**	0	0	0	0	0	0	0	0	0.09	0	0	0	0.12	4.11	0.46	0.07
**S4-A-CTRL**	0	0	0	0	0	0	0	0	0	0	0	0	0	0.09	0	0
**S4-B-CTRL**	0	0	0	0	0	0	0	0	0	0	0	0	0	0.06	0.04	0
**S4-C-CTRL**	0	0	0	0	0	0	0	0	0	0	0	0	0	0.04	0.04	0
**S4-A-PPC**	0	0	0	0	0	0	0	0	0	0	0	0	0	0.47	0.33	0
**S4-B-PPC**	0	0	0	0	0	0	0	0	0	0	0	0.2	0	0.62	0.31	0
**S4-C-PPC**	0	0	0	0	0	0	0	0	0	0	0	0	0	0.38	0	0
**S4-A-PPI**	0	0	0	0	0	0	0	0	0	0	0	0	0	0.04	0.05	0
**S4-B-PPI**	0	0	0	0	0	0	0	0	0	0	0	0	0	0.07	0	0
**S4-C-PPI**	0	0	0	0	0	0	0	0	0	0	0	0	0	0.05	0.04	0
**S5-A-CTRL**	0	0	0	0	0	0	0	0	0	0	0	0	0	0	0	0
**S5-B-CTRL**	0	0	0	0	0	0	0	0	0	0	0	0	0	0	0	0
**S5-C-CTRL**	0	0	0	0	0	0	0	0	0	0	0	0	0	0	0	0
**S5-A-PPC**	0	0	0	0	0	0	0	0	0	0	0	0	0.23	0	0	0
**S5-B-PPC**	0	0	0	0	0	0	0	0	0	0	0	0	0	0	0	0
**S5-C-PPC**	0	0	0	0	0	0	0	0	0	0	0	0	0	0	0	0
**S5-A-PPI**	0	0	0	0	0	0	0	0	0	0	0	0	0	0	0	0
**S5-B-PPI**	0	0	0	0	0	0	0	0	0	0	0	0	0	0	0	0
**S5-C-PPI**	0	0	0	0	0	0	0	0	0	0	0	0	0	0	0	0
**S6-A-CTRL**	0	0.24	0	0	0	0	0	0	0	0	0	0.11	0.46	4.05	0.61	0.11
**S6-B-CTRL**	0	0	0	0	0	0	0	0	0	0	0	0.13	0.56	3.48	0.59	0.23
**S6-C-CTRL**	0	0	0	0	0	0	0	0	0	0	0	0.12	0.59	3.6	0.51	0.16
**S6-A-PPC**	0	0	0	0	0	0	0	0	0	0	0	0	0.03	0.28	0.02	0
**S6-B-PPC**	0	0	0	0	0	0	0	0	0	0	0	0	0.03	0.24	0.02	0
**S6-C-PPC**	0	0	0	0	0	0	0	0	0	0	0	0	0.03	0.22	0.02	0
**S6-A-PPI**	0	0.11	0	0	0	0	0	0	0	0	0.05	0	0.15	0.75	0.11	0
**S6-B-PPI**	0	0.13	0	0	0	0	0	0	0	0	0	0	0.13	1.13	0.13	0.05
**S6-C-PPI**	0	0.12	0	0	0	0	0	0	0	0	0	0.04	0.13	0.86	0.13	0.05

Bacterial species	Simiaoa below species threshold	Simiaoa sunii	Slackia below species threshold	Sodaliphilus below species threshold	Sodaliphilus pleomorphus	Solibaculum below species threshold	Sphingobacterium below species threshold	Sphingomonas below species threshold	Sporobacter below species threshold	Streptococcus agalactiae	Streptococcus anginosus	Streptococcus australis	Streptococcus below species threshold	Streptococcus caecimuris	Streptococcus canis	Streptococcus constellatus
**S1-A-CTRL**	0	0	0	0	0	0	0	0	0	0	0	0	1.16	0	0	0
**S1-B-CTRL**	0	0	0	0	0	0	0	0	0	0	0	0	3.07	0	0	0
**S1-C-CTRL**	0	0	0	0	0	0	0	0	0	0	0	0	1.01	0	0	0
**S1-A-PPC**	0	0	0	0	0	0	0	0	0	0	0	0	3.92	0	0.51	0
**S1-B-PPC**	0	0	0	0	0	0	0	0	0	0	0	0	4.05	0	0.42	0
**S1-C-PPC**	0	0	0	0	0	0	0	0	0	0	0	0	3.61	0	0.48	0
**S1-A-PPI**	0	0	0	0	0	0	0	0	0	0	0	0	2.28	0	0	0
**S1-B-PPI**	0	0	0	0	0	0	0	0	0	0	0	0	3.55	0	0.45	0
**S1-C-PPI**	0	0	0	0	0	0	0	0	0	0	0	0	1.59	0	0.29	0
**S2-A-CTRL**	0	0	0	0	0	0	0	0	0	0	0	0.53	1.44	0.06	0	0
**S2-B-CTRL**	0	0	0	0	0	0	0	0	0	0	0	0.77	1.29	0.07	0	0
**S2-C-CTRL**	0	0	0	0	0	0	0	0	0	0	0	0.52	1.11	0.04	0	0
**S2-A-PPC**	0	0	0	0	0	0	0	0	0.02	0	0	0.34	4.05	0	0	0
**S2-B-PPC**	0	0	0	0	0	0	0	0	0	0	0	0.25	2.85	0	0	0
**S2-C-PPC**	0	0	0	0	0	0	0	0	0	0	0	0.28	3.74	0	0	0
**S2-A-PPI**	0	0.02	0	0	0	0	0	0	0	0	0	0.31	2.68	0.04	0	0
**S2-B-PPI**	0	0.02	0	0	0	0	0	0	0	0	0	0.38	3.14	0.04	0	0
**S2-C-PPI**	0	0.02	0	0	0	0	0	0	0	0	0	0.26	2.71	0.03	0	0
**S3-A-CTRL**	0	0	0	0	0	0	0	0	0	0	0	0	1.81	0	0	0
**S3-B-CTRL**	0	0	0	0	0	0	0	0	0	0	0	0	2.17	0	0	0
**S3-C-CTRL**	0	0	0	0	0	0	0	0	0	0	0	0	2.7	0	0	0
**S3-A-PPC**	0	0	0	0	0	0	0	0	0	0.13	0.04	0	4.77	0	0.11	0
**S3-B-PPC**	0	0	0	0	0	0	0	0	0	0.11	0.06	0	5.05	0	0.14	0.04
**S3-C-PPC**	0	0	0	0	0	0	0	0	0	0.07	0	0	4.83	0	0.16	0.04
**S3-A-PPI**	0	0	0	0	0	0	0	0	0	0	0	0	2.36	0	0	0
**S3-B-PPI**	0	0	0	0	0	0	0	0	0	0	0	0	2.31	0	0	0
**S3-C-PPI**	0	0	0	0	0	0	0	0	0	0	0	0	1.94	0	0	0
**S4-A-CTRL**	0	0	0	0	0	0	0	0	0	0	0.5	0	0.47	0	0	0
**S4-B-CTRL**	0	0	0	0	0	0	0	0	0	0	0.22	0	0.31	0	0	0
**S4-C-CTRL**	0	0	0	0	0	0	0	0	0	0	0.44	0	0.61	0	0	0
**S4-A-PPC**	0	0	0	0	0	0	0	0	0	0	0.96	0	1.72	0	0	0
**S4-B-PPC**	0	0	0	0	0	0	0	0	0	0	1.92	0	1.77	0	0	0
**S4-C-PPC**	0	0	0	0	0	0	0	0	0	0	1.08	0	1.55	0	0	0
**S4-A-PPI**	0	0	0	0	0	0	0	0	0	0	1.98	0	1.83	0	0	0.12
**S4-B-PPI**	0	0	0	0	0	0	0	0	0	0	1.18	0	1.11	0	0	0
**S4-C-PPI**	0	0	0	0	0	0	0	0	0	0	1.88	0	1.73	0	0.12	0.14
**S5-A-CTRL**	0	0	0	0	0	0	0	0	0	0	0.69	0	0	0	0	0
**S5-B-CTRL**	0	0	0	0	0	0	0	1.09	0	0	0.66	0	0	0	0	0
**S5-C-CTRL**	0	0	0	0	0	0	0	0.33	0	0	0.68	0	0	0	0	0
**S5-A-PPC**	0	0	0	0	0	0	0	0	0	0	3.99	0	2.02	0	0	0
**S5-B-PPC**	0	0	0	0	0	0	0	0	0	0	3.5	0	2.03	0	0	0
**S5-C-PPC**	0	0	0	0	0	0	0	0	0	0	3.69	0	2.09	0	0	0
**S5-A-PPI**	0	0	0	0	0	0	0	0	0	0	2.84	0	1.9	0	0	0
**S5-B-PPI**	0	0	0	0	0	0	0	0	0	0	4.09	0	1.97	0	0	0
**S5-C-PPI**	0	0	0	0	0	0	0	0	0	0	4.1	0	1.54	0	0	0
**S6-A-CTRL**	0	0	0	0	0	0	0	0	0	0	0	0	0	0	0	0
**S6-B-CTRL**	0	0	0	0	0	0	0	0	0	0	0	0	0	0	0	0
**S6-C-CTRL**	0	0	0	0	0	0	0	0	0	0	0	0	0	0	0	0
**S6-A-PPC**	0	0.04	0	0	0	0	0	0	0	0	0	0	0.14	0	0	0
**S6-B-PPC**	0	0	0	0	0.03	0	0	0	0	0	0	0	0.09	0	0	0
**S6-C-PPC**	0	0.03	0	0	0.03	0	0	0	0	0	0	0	0.09	0	0	0
**S6-A-PPI**	0	0.07	0	0	0	0	0	0	0	0	0	0	0	0	0	0
**S6-B-PPI**	0	0.07	0	0	0.07	0	0	0	0	0	0	0	0	0	0	0
**S6-C-PPI**	0	0.05	0	0	0	0	0	0	0	0	0	0	0.08	0	0	0

**Bacterial species**	Streptococcus dysgalactiae	Streptococcus equinus	Streptococcus gallolyticus	Streptococcus gordonii	Streptococcus henryi	Streptococcus humanilactis	Streptococcus infantarius	Streptococcus infantis	Streptococcus intermedius	Streptococcus koreensis	Streptococcus lutetiensis	Streptococcus mitis	Streptococcus multihit species	Streptococcus oralis	Streptococcus parasanguinis	Streptococcus pneumoniae
**S1-A-CTRL**	0	0	7.63	0	0	0	0	0	0	0	0	0	0.91	0	0	0
**S1-B-CTRL**	0	0	12.29	0	0	0	0	0	0	0	0	0	2.08	0	0	0
**S1-C-CTRL**	0	0	8.59	0	0	0	0	0	0	0	0	0	0.78	0	0	0
**S1-A-PPC**	0	0	49.77	0	0	0	0	0	0	0	0.22	0	2.62	0	0	0
**S1-B-PPC**	0	0	48.38	0	0	0	0	0	0	0	0	0	2.78	0	0	0
**S1-C-PPC**	0	0	48.58	0	0	0	0	0	0	0	0.28	0	2.67	0	0	0
**S1-A-PPI**	0	0	21.46	0	0	0	0	0	0	0	0	0	1.12	0	0	0
**S1-B-PPI**	0	0	37.75	0	0	0	0	0	0	0	0	0	2.14	0	0	0
**S1-C-PPI**	0	0	16.12	0	0	0	0	0	0	0	0	0	1.05	0	0	0
**S2-A-CTRL**	0	0	0	0.05	0	0	0	0.06	0	0	0	0.03	5.6	0.02	25.2	0
**S2-B-CTRL**	0	0	0	0.09	0	0	0	0.06	0	0.03	0	0.03	5.92	0.03	27.85	0
**S2-C-CTRL**	0	0	0	0.06	0	0	0	0.07	0	0	0	0.03	5.28	0.03	22.22	0
**S2-A-PPC**	0	0	0	0.03	0	0	0	0	0	0	0	0	8.2	0	11.42	0
**S2-B-PPC**	0	0	0	0	0	0	0	0	0	0.03	0	0	5.84	0	10.25	0
**S2-C-PPC**	0	0	0	0	0	0	0	0	0	0	0	0	7.76	0	11.87	0
**S2-A-PPI**	0	0	0	0	0	0	0	0	0	0	0	0.04	6.34	0	11.79	0
**S2-B-PPI**	0	0	0	0.03	0	0	0	0	0	0.03	0	0	6.94	0	14.17	0
**S2-C-PPI**	0	0	0	0	0	0	0	0.05	0	0	0	0	5.91	0.03	12.44	0
**S3-A-CTRL**	0	0	0.79	0	0	0	0	0	0	0	0	0	0	0	0	0
**S3-B-CTRL**	0	0	0.69	0	0	0	0	0	0	0	0	0	0	0	0	0
**S3-C-CTRL**	0	0	0.83	0	0	0	0	0	0	0	0.41	0	0	0	0	0
**S3-A-PPC**	0.04	0.22	38.11	0	0.03	0	0.12	0	0.34	0	1.37	0	2.75	0	0.03	0
**S3-B-PPC**	0.04	0.23	36.54	0	0.07	0	0.16	0	0.32	0	1.31	0	2.92	0	0.06	0.05
**S3-C-PPC**	0	0.15	37.92	0	0.05	0	0.11	0	0.38	0	1.58	0	2.78	0	0.04	0
**S3-A-PPI**	0	0.24	2.14	0	0	0	0	0	0	0	0.53	0	0	0	0	0
**S3-B-PPI**	0	0.31	4.66	0	0	0	0	0	0	0	0.43	0	0.25	0	0	0
**S3-C-PPI**	0	0.32	1.82	0	0	0	0	0	0	0	0.31	0	0.18	0	0	0
**S4-A-CTRL**	0	0	0	0	0	0	0	0	0	0	0	0	3.84	0	0	0
**S4-B-CTRL**	0	0	0	0	0	0	0	0	0	0	0	0	2.75	0	0	0
**S4-C-CTRL**	0	0	0	0	0	0	0	0	0	0	0	0	5.48	0.09	0	0
**S4-A-PPC**	0	0	0	0	0	0	0	0	0	0	0	0	15.33	0	0	0
**S4-B-PPC**	0	0	0	0	0	0	0	0	0	0	0	0	15.39	0	0	0
**S4-C-PPC**	0	0	0	0	0	0	0	0	0	0	0	0	15.23	0	0	0
**S4-A-PPI**	0	0	0	0	0	0	0	0	0	0	0	0.24	18.45	0.27	0	0
**S4-B-PPI**	0	0	0	0	0	0	0	0	0.08	0	0	0.08	13.8	0.23	0	0
**S4-C-PPI**	0	0	0	0	0	0	0	0	0	0	0	0.14	28.88	0.48	0	0.08
**S5-A-CTRL**	0	0	0	0	0	0	0	0	0	0	0	0	5.51	0	0	0
**S5-B-CTRL**	0	0	0	0	0	0	0	0	0	0	0	0	4.89	0	0	0
**S5-C-CTRL**	0	0	0	0	0	0	0	0	0	0	0	0	9.04	0	0	0
**S5-A-PPC**	0	0	0	0	0	0	0	0	0	0	0	0.62	31.69	0.53	0	0
**S5-B-PPC**	0	0	0	0	0	0	0	0	0	0	0	0	29.62	0.5	0	0
**S5-C-PPC**	0	0	0	0	0	0	0	0	0	0	0	0	33.32	0.41	0	0
**S5-A-PPI**	0	0	0	0	0	0	0	0	0	0	0	0	22.7	0	0	0
**S5-B-PPI**	0	0	0	0	0	0	0	0	0	0	0	0	33.45	0.44	0	0
**S5-C-PPI**	0	0	0	0	0	0	0	0	0.26	0	0	0	35.15	0.28	0	0
**S6-A-CTRL**	0	0	0	0	0	0	0	0	0	0	0	0	0	0	0	0
**S6-B-CTRL**	0	0	0	0	0	0	0	0	0	0	0	0	0	0	0	0
**S6-C-CTRL**	0	0	0	0	0	0	0	0	0	0	0	0	0	0	0	0
**S6-A-PPC**	0	0	0	0	0	0	0	0	0	0	0	0.28	0.1	0	0	0
**S6-B-PPC**	0	0	0	0	0	0.07	0	0	0	0	0	0.35	0.12	0	0	0
**S6-C-PPC**	0	0	0	0	0	0	0	0	0	0	0	0.38	0.14	0	0	0
**S6-A-PPI**	0	0	0	0	0	0.1	0	0	0	0	0	0.36	0.13	0	0	0
**S6-B-PPI**	0	0	0	0	0	0	0	0	0	0	0	0.3	0	0	0	0
**S6-C-PPI**	0	0	0	0	0	0	0	0	0	0	0	0.3	0.17	0	0	0

Bacterial species	Streptococcus pyogenes	Streptococcus raffinosi	Streptococcus rubneri	Streptococcus ruminicola	Streptococcus salivarius	Streptococcus sanguinis	Streptococcus suis	Streptococcus thermophilus	Streptococcus vaginalis	Streptococcus vestibularis	Streptococcus xiaochunlingii	Subdoligranulum below species threshold	Subdoligranulum variabile	Suilimivivens aceti	Suilimivivens below species threshold	Sutterella below species threshold
**S1-A-CTRL**	0	0	0	0	0	0	0	0	0	0	0	0	0	0	0	0
**S1-B-CTRL**	0	0	0	0	0	0	0	0	0	0	0	0	0	0	0	0
**S1-C-CTRL**	0	0	0	0	0	0	0	0	0	0	0	0	0	0	0	0
**S1-A-PPC**	0	0	0	0	0	0	0.24	0	0	0	0	0	0	0	0	0
**S1-B-PPC**	0	0	0	0	0	0	0.4	0	0	0	0	0	0	0	0	0
**S1-C-PPC**	0	0	0	0	0	0	0.33	0	0	0	0	0	0	0	0	0
**S1-A-PPI**	0	0	0	0	0	0	0	0	0	0	0	0	0	0	0	0
**S1-B-PPI**	0	0	0	0	0	0	0	0	0	0	0	0	0	0	0	0
**S1-C-PPI**	0	0	0	0	0	0	0.22	0	0	0	0	0	0	0	0	0
**S2-A-CTRL**	0	0	0.05	0	0.17	0.03	0	0	0	0	0.14	0.01	0	0	0	0
**S2-B-CTRL**	0	0	0.07	0	0.15	0	0	0	0	0.07	0.18	0	0	0	0	0
**S2-C-CTRL**	0	0	0.05	0	0.13	0.03	0	0	0	0.03	0.17	0	0	0	0	0
**S2-A-PPC**	0	0.06	0	0	0.41	0.04	0	0.05	0	0.11	0.07	0.02	0	0	0	0
**S2-B-PPC**	0	0.04	0.03	0	0.33	0.03	0	0	0	0.06	0.06	0	0	0	0	0
**S2-C-PPC**	0	0.05	0	0	0.41	0.04	0	0.04	0	0.1	0.06	0	0	0	0	0
**S2-A-PPI**	0	0	0	0	0.32	0.03	0	0	0	0.07	0.12	0.03	0	0	0	0
**S2-B-PPI**	0	0.05	0.03	0	0.37	0	0	0	0	0.11	0.1	0	0	0	0	0
**S2-C-PPI**	0	0.03	0.04	0	0.31	0	0	0.03	0	0.09	0.09	0	0	0	0	0
**S3-A-CTRL**	0	0	0	0	0	0	0	0	0	0	0	0	0	0	0	0.31
**S3-B-CTRL**	0	0	0	0	0	0	0	0	0	0	0	0	0	0	0	0
**S3-C-CTRL**	0	0	0	0	0	0	0	0	0	0	0	0	0	0	0	0
**S3-A-PPC**	0.03	0	0	0	0	0	0.35	0	0	0.71	0	0	0	0	0	0
**S3-B-PPC**	0	0	0	0.03	0	0	0.45	0	0	0.81	0	0	0	0	0	0
**S3-C-PPC**	0	0	0	0	0	0	0.4	0.04	0	0.72	0	0	0	0	0	0
**S3-A-PPI**	0	0	0	0	0	0	0	0	0	0	0	0	0	0	0	0
**S3-B-PPI**	0	0	0	0	0	0	0	0	0	0	0	0	0	0	0	0
**S3-C-PPI**	0	0	0	0	0	0	0	0	0	0	0	0	0	0	0	0
**S4-A-CTRL**	0	0	0	0	0	0	0	0	0.93	0	0	0	0	0	0	0
**S4-B-CTRL**	0	0	0	0	0	0	0	0	0.8	0	0	0	0	0	0	0
**S4-C-CTRL**	0	0	0	0	0	0	0	0	1.13	0.12	0	0	0	0	0	0
**S4-A-PPC**	0	0	0	0	0	0	0	0	2.35	0	0	0	0	0	0	0
**S4-B-PPC**	0	0	0	0	0	0	0	0	2.48	0	0	0	0	0	0	0
**S4-C-PPC**	0	0	0	0	0	0	0	0	2.37	0	0	0	0	0	0	0
**S4-A-PPI**	0	0	0	0	0	0	0.13	0	3.62	0.5	0	0	0	0	0	0
**S4-B-PPI**	0	0	0	0	0	0	0.09	0	2.37	0.32	0	0	0	0	0	0.06
**S4-C-PPI**	0	0	0	0	0	0	0.2	0	3.49	0.68	0	0	0	0	0	0
**S5-A-CTRL**	0	0	0	0	0	0	0	0	0	0	0	0	0	0	0	0
**S5-B-CTRL**	0	0	0	0	0	0	0	0	0	0	0	0	0	0	0	0
**S5-C-CTRL**	0	0	0	0	0	0	0	0	0	0	0	0	0	0	0	0
**S5-A-PPC**	0	0	0	0	0	0	0	0	2.48	0	0	0	0	0	0	0
**S5-B-PPC**	0	0	0	0	0	0	0	0	2.21	0	0	0	0	0	0	0
**S5-C-PPC**	0	0	0	0	0	0	0	0.57	2.43	0	0	0	0	0	0	0
**S5-A-PPI**	0	0	0	0	0	0	0	0	1.39	0	0	0	0	0	0	0
**S5-B-PPI**	0	0	0	0	0	0	0.25	0	2.41	0	0	0	0	0	0	0
**S5-C-PPI**	0	0	0	0	0	0	0	0	2.17	0	0	0	0	0	0	0
**S6-A-CTRL**	0	0	0	0	0	0	0	0	0	0	0	0	0	0	0	0
**S6-B-CTRL**	0	0	0	0	0	0	0	0	0	0	0	0	0	0	0	0
**S6-C-CTRL**	0	0	0	0	0	0	0	0	0	0	0	0	0	0	0	0
**S6-A-PPC**	0	0	0	0	0	0	0	0	0	0	0	0	0	0	0	0
**S6-B-PPC**	0	0	0	0	0	0	0	0	0	0	0	0	0	0	0	0
**S6-C-PPC**	0	0	0	0	0	0	0	0	0	0	0	0	0	0	0	0
**S6-A-PPI**	0	0	0	0	0	0	0	0	0	0	0	0	0	0	0	0
**S6-B-PPI**	0	0	0	0	0	0	0	0	0	0	0	0	0	0	0	0
**S6-C-PPI**	0	0	0	0	0	0	0	0	0	0	0	0	0	0	0	0

**Bacterial species**	Sutterella wadsworthensis	Thalassospira below species threshold	Thomasclavelia below species threshold	Treponema below species threshold	Turicibacter bilis	Turicibacter sanguinis	Veillonella below species threshold	Velocimicrobium below species threshold	Ventrimonas below species threshold	Ventrimonas faecis	Vermiculatibacterium below species threshold	Vescimonas below species threshold	Vescimonas coprocola	Vescimonas fastidiosa	Victivallis below species threshold	Victivallis lenta
**S1-A-CTRL**	3.9	0	0	0	0	0	0	0	0	0	0	0	0	0	0	0
**S1-B-CTRL**	1.07	0	0	0	0	0	0	0	0	0	0	0	0	0	0	0
**S1-C-CTRL**	2.59	0	0	0	0	0	0	0	0	0	0	0	0	0	0	0
**S1-A-PPC**	0.25	0	0	0	0	0	0	0	0	0	0	0	0	0	0	0
**S1-B-PPC**	0.26	0	0	0	0	0	0	0	0	0	0	0	0	0	0	0
**S1-C-PPC**	0.27	0	0	0	0	0	0	0	0	0	0	0	0	0	0	0
**S1-A-PPI**	0.46	0	0	0	0	0	0	0	0	0	0	0	0	0	0	0
**S1-B-PPI**	0.28	0	0	0	0	0	0	0	0	0	0	0	0	0	0	0
**S1-C-PPI**	0.61	0	0	0	0	0	0	0	0	0	0	0	0	0	0	0
**S2-A-CTRL**	0	0	0	0	0	0	0	0	0	0	0	0	0	0	0	0
**S2-B-CTRL**	0	0	0	0	0	0	0	0	0	0	0	0	0	0	0	0
**S2-C-CTRL**	0	0	0	0	0	0	0	0	0	0	0	0	0	0	0	0
**S2-A-PPC**	0	0	0	0	0	0	0	0	0	0	0	0	0	0	0	0
**S2-B-PPC**	0	0	0	0	0	0	0	0	0	0	0	0.04	0	0	0	0
**S2-C-PPC**	0	0	0	0	0	0	0	0	0	0	0	0.02	0	0	0	0
**S2-A-PPI**	0	0	0	0	0	0	0	0	0	0	0	0.04	0	0	0	0
**S2-B-PPI**	0	0	0	0	0	0	0	0	0	0	0	0.02	0	0	0	0
**S2-C-PPI**	0	0	0	0	0	0	0	0	0	0	0	0.04	0	0	0	0
**S3-A-CTRL**	23.61	0	0	0	0	0	0	0	0	0	0	0	0	0	0	0
**S3-B-CTRL**	7.28	0	0	0	0	0	0	0	0	0	0	0	0	0	0	0
**S3-C-CTRL**	6.62	0	0	0	0	0	0	0	0	0	0	0.45	0	0	0	0
**S3-A-PPC**	0.11	0	0	0	0	0	0	0	0	0	0	0.04	0	0	0	0
**S3-B-PPC**	0.13	0	0	0	0	0	0	0	0	0	0	0.03	0	0	0	0
**S3-C-PPC**	0.19	0	0	0	0	0	0	0	0	0	0	0.03	0	0	0	0
**S3-A-PPI**	1.05	0	0	0	0	0	0	0	0	0	0	0.18	0	0	0	0
**S3-B-PPI**	0.2	0	0	0	0	0	0	0	0	0	0	0.12	0	0	0	0
**S3-C-PPI**	0.43	0	0	0	0	0	0	0	0	0	0	0.19	0	0	0	0
**S4-A-CTRL**	0	0	0	0	0	0	0	0	0	0	0	0	0	0	0	0
**S4-B-CTRL**	0	0	0	0	0	0	0	0	0	0	0	0	0	0	0	0
**S4-C-CTRL**	0	0	0	0	0	0	0	0	0	0	0	0	0	0	0	0
**S4-A-PPC**	0	0	0	0	0	0	0	0	0	0	0	0	0	0	0	0
**S4-B-PPC**	0	0	0	0	0	0	0	0	0	0	0	0	0	0	0	0
**S4-C-PPC**	0	0	0	0	0	0	0	0	0	0	0	0	0	0	0	0
**S4-A-PPI**	0	0	0	0	0	0.08	0	0	0	0	0	0	0	0	0	0
**S4-B-PPI**	0	0	0	0	0	0.05	0	0	0	0	0	0	0	0	0	0
**S4-C-PPI**	0	0	0	0	0	0.05	0	0	0	0	0	0	0	0	0	0
**S5-A-CTRL**	0	0	0	0	0	0	0	0	0	0	0	0	0	0	0	0
**S5-B-CTRL**	0	0	0	0	0	0	0	0	0	0	0	0	0	0	0	0
**S5-C-CTRL**	0	0	0	0	0	0	0	0	0	0	0	0	0	0	0	0
**S5-A-PPC**	0	0	0	0	0	0	0	0	0	0	0	0	0	0	0	0
**S5-B-PPC**	0	0	0	0	0	0	0	0	0	0	0	0	0	0	0	0
**S5-C-PPC**	0	0	0	0	0	0	0	0	0	0	0	0	0	0	0	0
**S5-A-PPI**	0	0	0	0	0	0	0	0	0	0	0	0	0	0	0	0
**S5-B-PPI**	0	0	0	0	0	0	0	0	0	0	0	0	0	0	0	0
**S5-C-PPI**	0	0	0	0	0	0	0	0	0	0	0	0	0	0	0	0
**S6-A-CTRL**	2.67	0	0	0	0	0	0	0	0	0	0	0.21	0	0	0	0
**S6-B-CTRL**	1.79	0	0	0	0	0	0	0	0	0	0	0.31	0	0	0	0
**S6-C-CTRL**	0.68	0	0	0	0	0	0	0	0	0	0	0.26	0	0	0	0
**S6-A-PPC**	0	0	0	0	0	0	0	0	0	0	0	0	0	0	0	0
**S6-B-PPC**	0	0	0	0	0	0	0	0	0	0	0	0.05	0	0	0	0
**S6-C-PPC**	0	0	0	0	0	0	0	0	0	0	0	0.04	0	0	0	0
**S6-A-PPI**	0	0	0	0	0	0	0	0	0	0	0	0.08	0	0	0	0
**S6-B-PPI**	0.07	0	0	0	0	0	0	0	0	0	0	0.21	0	0	0	0
**S6-C-PPI**	0.07	0	0	0	0	0	0	0	0	0	0	0.13	0	0	0	0

**Bacterial species**	Victivallis vadensis	Waltera acetigignens	Waltera below species threshold	Waltera intestinalis	Waltera multihit species	Wansuia below species threshold	Weissella below species threshold	Weissella cibaria	Weissella confusa	Weissella multihit species	Xanthomonas below species threshold	Xylanibacter below species threshold	Xylanibacter brevis	Yanshouia below species threshold	Yanshouia hominis	Yeguia below species threshold
**S1-A-CTRL**	0	0.43	0	0	0	0	0	0	0	0	0	0	0	0	0	0
**S1-B-CTRL**	0	0.24	0	0	0	0	0	0	0	0	0	0	0	0	0	0
**S1-C-CTRL**	0	0.22	0	0	0	0	0	0	0	0	0	0	0	0	0	0
**S1-A-PPC**	0	0	0	0	0	0	0	0	0	0	0	0	0	0	0	0
**S1-B-PPC**	0	0.18	0	0	0	0	0	0	0	0	0	0	0	0	0	0
**S1-C-PPC**	0	0.18	0	0	0	0	0	0	0	0	0	0	0	0	0	0
**S1-A-PPI**	0	0	0	0	0	0	0	0	0	0	0	0	0	0	0	0
**S1-B-PPI**	0	0	0	0	0	0	0	0	0	0	0	0	0	0	0	0
**S1-C-PPI**	0	0.2	0	0	0	0	0	0	0	0	0	0	0	0	0	0
**S2-A-CTRL**	0	0.02	0	0	0	0	0	0	0	0	0	0	0	0	0	0
**S2-B-CTRL**	0	0	0	0	0	0	0	0	0	0	0	0	0	0	0	0
**S2-C-CTRL**	0	0.02	0	0	0	0	0	0	0	0	0	0	0	0	0	0
**S2-A-PPC**	0	0.47	0.05	0	0	0	0	0	0	0	0	0	0	0	0	0
**S2-B-PPC**	0	0.41	0.02	0	0	0	0	0	0	0	0	0	0	0	0	0
**S2-C-PPC**	0	0.38	0.02	0	0	0	0	0	0	0	0	0	0	0	0	0
**S2-A-PPI**	0	0.44	0.02	0	0	0	0	0	0	0	0	0	0	0	0	0
**S2-B-PPI**	0	0.46	0.03	0	0	0	0	0	0	0	0	0	0	0	0	0
**S2-C-PPI**	0	0.4	0.02	0	0	0	0	0	0	0	0	0	0	0	0	0
**S3-A-CTRL**	0	0	0	0	0	0	0	0.78	0	0	0	0.58	0	0	0	0
**S3-B-CTRL**	0	0	0	0	0	0	0	1.09	0	0	0	0.8	0	0	0	0
**S3-C-CTRL**	0	0	0	0	0	0	0	0.89	0	0	0	0.82	0	0	0	0
**S3-A-PPC**	0	0.03	0	0	0	0	0.17	11.71	1.37	0.26	0	0.09	0	0	0	0
**S3-B-PPC**	0	0.02	0	0	0	0	0.2	12.25	1.27	0.22	0	0.12	0	0	0	0
**S3-C-PPC**	0	0.02	0	0	0	0	0.2	10.44	1.62	0.23	0	0.09	0	0	0	0
**S3-A-PPI**	0	0	0	0	0	0	0	3.89	0.62	0	0	0.33	0	0	0	0
**S3-B-PPI**	0	0	0	0	0	0	0.17	8.34	1.29	0.23	0	0.39	0	0	0	0
**S3-C-PPI**	0	0	0	0	0	0	0	6.1	1.32	0.2	0	0.3	0	0	0	0
**S4-A-CTRL**	0	0	0	0	0	0	0	0	0	0	0	0	0	0	0	0
**S4-B-CTRL**	0	0	0	0	0	0	0	0	0	0	0	0	0	0	0	0
**S4-C-CTRL**	0	0	0	0	0	0	0	0	0	0	0	0	0	0	0	0
**S4-A-PPC**	0	0	0	0	0	0	0	0	0	0	0	0	0	0	0	0
**S4-B-PPC**	0	0	0	0	0	0	0	0	0	0	0	0	0	0	0	0
**S4-C-PPC**	0	0	0	0	0	0	0	0	0	0	0	0	0	0	0	0
**S4-A-PPI**	0	0	0	0	0	0	0	0	0	0	0	0	0	0	0	0
**S4-B-PPI**	0	0	0	0	0	0	0	0	0	0	0	0	0	0	0.05	0
**S4-C-PPI**	0	0	0	0	0	0	0	0	0	0	0	0	0	0	0	0
**S5-A-CTRL**	0	0	0	0	0	0	0	0	0	0	0	0	0	0	0	0
**S5-B-CTRL**	0	0	0	0	0	0	0	0	0	0	0	0	0	0	0	0
**S5-C-CTRL**	0	0	0	0	0	0	0	0	0	0	0	0	0	0	0	0
**S5-A-PPC**	0	0	0	0	0	0	0	0	0	0	0	0	0	0	0	0
**S5-B-PPC**	0	0	0	0	0	0	0	0	0	0	0	0	0	0	0	0
**S5-C-PPC**	0	0	0	0	0	0	0	0	0	0	0	0	0	0	0	0
**S5-A-PPI**	0	0	0	0	0	0	0	0	0	0	0	0	0	0	0	0
**S5-B-PPI**	0	0	0	0	0	0	0	0	0	0	0	0	0	0	0	0
**S5-C-PPI**	0	0	0	0	0	0	0	0	0	0	0	0	0	0	0	0
**S6-A-CTRL**	0	0	0	0	0	0	0	0	0	0	0	0	0	0	0	0
**S6-B-CTRL**	0	0	0	0	0	0	0	0	0	0	0	0	0	0	0	0
**S6-C-CTRL**	0	0	0	0	0	0	0	0	0	0	0	0	0	0	0	0
**S6-A-PPC**	0	0.08	0	0	0	0	0	0	0	0	0	0	0	0	0	0
**S6-B-PPC**	0	0.08	0	0	0	0	0	0	0	0	0	0	0	0	0	0
**S6-C-PPC**	0	0.08	0	0	0	0	0	0	0	0	0	0	0	0	0	0
**S6-A-PPI**	0	0.11	0	0	0	0	0	0	0	0	0	0	0	0	0	0
**S6-B-PPI**	0	0.17	0	0	0	0	0	0	0	0	0	0	0	0	0	0
**S6-C-PPI**	0	0.15	0	0.07	0	0	0	0	0	0	0	0	0	0	0	0

**Bacterial species**	Yeguia hominis	Yersinia rohdei	Youxingia below species threshold	Zongyangia below species threshold
**S1-A-CTRL**	0	0	0	0
**S1-B-CTRL**	0	0.31	0	0
**S1-C-CTRL**	0	0.17	0	0
**S1-A-PPC**	0	0	0	0
**S1-B-PPC**	0	0.13	0	0
**S1-C-PPC**	0	0	0	0
**S1-A-PPI**	0	0.21	0	0
**S1-B-PPI**	0	0	0	0
**S1-C-PPI**	0	0.1	0	0
**S2-A-CTRL**	0	0	0	0
**S2-B-CTRL**	0	0	0	0
**S2-C-CTRL**	0	0	0	0
**S2-A-PPC**	0	0	0	0
**S2-B-PPC**	0	0	0	0
**S2-C-PPC**	0	0	0	0
**S2-A-PPI**	0	0	0	0
**S2-B-PPI**	0	0	0	0
**S2-C-PPI**	0	0	0	0
**S3-A-CTRL**	0	0	0	0
**S3-B-CTRL**	0	0	0	0
**S3-C-CTRL**	0	0	0	0
**S3-A-PPC**	0	0	0	0
**S3-B-PPC**	0	0	0	0
**S3-C-PPC**	0	0	0	0
**S3-A-PPI**	0	0	0	0
**S3-B-PPI**	0	0	0	0
**S3-C-PPI**	0	0	0	0
**S4-A-CTRL**	0	0	0	0
**S4-B-CTRL**	0	0	0	0
**S4-C-CTRL**	0	0	0	0
**S4-A-PPC**	0	0	0	0
**S4-B-PPC**	0	0	0	0
**S4-C-PPC**	0	0	0	0
**S4-A-PPI**	0	0	0	0
**S4-B-PPI**	0	0	0	0
**S4-C-PPI**	0	0	0	0
**S5-A-CTRL**	0	0	0	0
**S5-B-CTRL**	0	0	0	0
**S5-C-CTRL**	0	0	0	0
**S5-A-PPC**	0	0	0	0
**S5-B-PPC**	0	0	0	0
**S5-C-PPC**	0	0	0	0
**S5-A-PPI**	0	0	0	0
**S5-B-PPI**	0	0	0	0
**S5-C-PPI**	0	0	0	0
**S6-A-CTRL**	0	0	0	0
**S6-B-CTRL**	0	0	0	0
**S6-C-CTRL**	0	0	0	0
**S6-A-PPC**	0	0	0	0
**S6-B-PPC**	0	0	0	0
**S6-C-PPC**	0	0	0	0
**S6-A-PPI**	0	0	0	0
**S6-B-PPI**	0	0	0	0
**S6-C-PPI**	0	0	0	0

Supplementary Table 6. Spearman correlation coefficients between nutritional parameters (g/100 g; protein, fat, fiber, carbohydrates, sugars, salt) and species-level relative abundances across the six microbial communities. *P*-values were adjusted per species using the Benjamini-Hochberg method

Nutritional parameters	Bacterial species	Correlation	Spearman correlation coefficients	P-value adjusted BH
Carbohydrates	*Bacteroides* multihit species	-0.4729	0.0003	0.0032
Carbohydrates	*Bacteroides ovatus*	-0.4423	0.0008	0.0076
Carbohydrates	*Bacteroides xylanisolvens*	-0.4367	0.0010	0.0088
Carbohydrates	*Phocaeicola* multihit species	-0.4118	0.0020	0.0163
Carbohydrates	*Phocaeicola vulgatus*	-0.3937	0.0032	0.0248
Carbohydrates	*Bacteroides faecium*	-0.3906	0.0035	0.0266
Carbohydrates	*Bacteroides vicugnae*	-0.3632	0.0070	0.0470
Carbohydrates	*Streptococcus suis*	0.3640	0.0068	0.0463
Carbohydrates	*Streptococcus canis*	0.3654	0.0066	0.0451
Carbohydrates	*Eubacterium* below species threshold	0.3699	0.0059	0.0411
Carbohydrates	*Enterococcus casseliflavus*	0.3720	0.0056	0.0394
Carbohydrates	*Dorea* multihit species	0.3846	0.0041	0.0302
Carbohydrates	*Faecalibacterium duncaniae*	0.4278	0.0013	0.0111
Carbohydrates	*Streptococcus* below species threshold	0.5029	0.0001	0.0013
Carbohydrates	*Ruminococcus* below species threshold	0.5190	0.0001	0.0008
Carbohydrates	*Bifidobacterium longum*	0.5850	0.0000	0.0001
Fat	*Bacteroides faecium*	-0.3906	0.0035	0.0266
Fat	*Faecalibacterium duncaniae*	0.3749	0.0052	0.0371
Fat	*Simiaoa sunii*	0.3815	0.0044	0.0323
Fat	*Lactococcus* below species threshold	0.3833	0.0042	0.0311
Fat	*Gemmiger* below species threshold	0.3878	0.0038	0.0282
Fat	*Clostridium* below species threshold	0.4247	0.0014	0.0119
Fat	*Ruminococcus* below species threshold	0.4755	0.0003	0.0030
Fat	*Dorea longicatena*	0.4780	0.0003	0.0028
Fiber	*Bacteroides* multihit species	-0.4729	0.0003	0.0032
Fiber	*Bacteroides ovatus*	-0.4423	0.0008	0.0076
Fiber	*Bacteroides xylanisolvens*	-0.4367	0.0010	0.0088
Fiber	*Phocaeicola* multihit species	-0.4118	0.0020	0.0163
Fiber	*Phocaeicola vulgatus*	-0.3937	0.0032	0.0248
Fiber	*Bacteroides faecium*	-0.3906	0.0035	0.0266
Fiber	*Bacteroides vicugnae*	-0.3632	0.0070	0.0470
Fiber	*Streptococcus suis*	0.3640	0.0068	0.0463
Fiber	*Streptococcus canis*	0.3654	0.0066	0.0451
Fiber	*Eubacterium* below species threshold	0.3699	0.0059	0.0411
Fiber	*Enterococcus casseliflavus*	0.3720	0.0056	0.0394
Fiber	*Dorea* multihit species	0.3846	0.0041	0.0302
Fiber	*Faecalibacterium duncaniae*	0.4278	0.0013	0.0111
Fiber	*Streptococcus* below species threshold	0.5029	0.0001	0.0013
Fiber	*Ruminococcus* below species threshold	0.5190	0.0001	0.0008
Fiber	*Bifidobacterium longum*	0.5850	0.0000	0.0001
Protein	*Bacteroides faecium*	-0.3906	0.0035	0.0266
Protein	*Faecalibacterium duncaniae*	0.3749	0.0052	0.0371
Protein	*Simiaoa sunii*	0.3815	0.0044	0.0323
Protein	*Lactococcus* below species threshold	0.3833	0.0042	0.0311
Protein	*Gemmiger* below species threshold	0.3878	0.0038	0.0282
Protein	*Clostridium* below species threshold	0.4247	0.0014	0.0119
Protein	*Ruminococcus* below species threshold	0.4755	0.0003	0.0030

Protein	*Dorea longicatena*	0.4780	0.0003	0.0028
Salt	*Clostridium* below species threshold	0.3604	0.0074	0.0497
Salt	*Dorea longicatena*	0.3619	0.0072	0.0482
Salt	*Simiaoa sunii*	0.3671	0.0063	0.0436
Salt	*Oscillibacter* below species threshold	0.3996	0.0028	0.0217
